# Unusual bonding situations in Th(iv) and U(iv)–Al(iii) pnictogen complexes

**DOI:** 10.1039/d5sc09143h

**Published:** 2026-03-18

**Authors:** Pritam Mahawar, Ganping Wang, Robert J. Ward, Steven P. Kelley, Laurent Maron, Justin R. Walensky

**Affiliations:** a Department of Chemistry, University of Missouri Columbia MO 65211 USA walenskyj@missouri.edu; b Université de Toulouse, INSA Toulouse, CNRS, LPCNO 31077 Toulouse France laurent.maron@irsamc.ups-tlse.fr

## Abstract

To enhance our understanding of the electronic structure of early actinides, a synthetic methodology of using an Al(i) precursor, [(C_5_Me_5_)Al]_4_, was applied to both thorium and uranium dipnictido complexes. The cleavage of the E–E bond in dipnictido actinide complexes, [(C_5_Me_5_)_2_An(*η*^2^-E_2_R_2_)], An = Th, U; E = N, P, As; R = Ph or 2,4,6-Me_3_C_6_H_2_ (Mes), with 0.25 equivalents of [Al(C_5_Me_5_)]_4,_ yields the heterobimetallic complexes, [(C_5_Me_5_)_2_An(*µ*_2_-ER)_2_Al(C_5_Me_5_)]. In the case of An = U, E = N, the U(vi) bis(imido) complex, [(C_5_Me_5_)_2_U(=NPh)_2_], is reduced by [(C_5_Me_5_)Al]. Based on the solid-state structures and DFT calculations, including Nucleus Independent Chemical Shift (NICS), all six complexes show aromaticity within the An–E–Al–E moiety. For Th, only σ-aromaticity is observed, but both σ + π aromaticity is observed in the U complexes.

## Abstract 2

To enhance our understanding of the electronic structure of early actinides, a synthetic methodology of using an Al(i) precursor, [(C_5_Me_5_)Al]_4_, was applied to both thorium and uranium dipnictido complexes.

## Graphical abstract

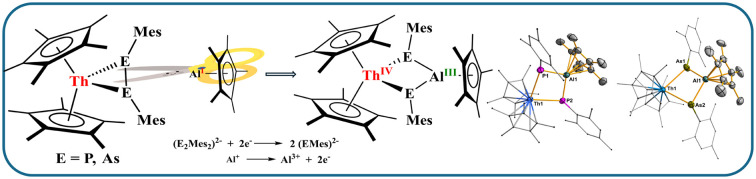

## Introduction

The coordination chemistry of the actinides seeks to further our understanding of their metal–ligand bonding and electronic structure.^1–6^ Comparisons in structure and bonding help to provide insight into the energy-driven covalency concept in which the 5f orbitals of the actinides become energetically degenerate with the *n*p ligand-based orbitals going down a group. However, a direct comparison of the bonding in structurally similar actinide complexes with nitrogen, phosphorus, and arsenic is rare.^7^

Low-valent group 13 chemistry continues to be an extensive area of exploration for the synthesis of unusual moieties and reactivity.^8–12^ Among these, aluminum(i) species, especially [Al(C_5_Me_5_)]_4_, have been used as strong Lewis bases to coordinate to transition metal and f elements, as well as activate bonds and small molecules.^13–19^ In the f elements, [Al(C_5_Me_5_)]_4_ has been primarily used to form metal–metal bonds,^20–24^ but our group has shown the reduction of U(vi) using Al(C_5_Me_5_) to produce a heterobimetallic, U(iv)/Al(iii), complex, [(C_5_Me_5_)_2_U{*µ*_2_-N(4-^i^PrOC_6_H_4_)}_2_Al(C_5_Me_5_)], and we sought to continue to investigate the reaction chemistry of actinide complexes with Al(C_5_Me_5_).^25^ To build upon this work, herein, we explore dipnictido complexes as we hypothesized that Al(C_5_Me_5_) would be a powerful enough reducing agent to break E–E bonds, E = N, P, As, in [(C_5_Me_5_)_2_An(*η*^2^-E_2_R_2_)], An = Th, U; R = Ph, Mes (2,4,6-Me_3_C_6_H_2_).^26*a*^ While the diphosphido and diarsenido complexes obtained *via* protonation of [(C_5_Me_5_)_2_An(Me_2_)], for both thorium and uranium, have been previously reported, the corresponding diamido (or hydrazinato) complexes have not been reported, and those reactions are included here for completeness. The combination of the solid-state structures and DFT calculations for each complex shows that σ aromaticity plays a role in the Th complex, while σ + π aromaticity is observed in the U case with two electrons coming from U(iv), a 5f^2^ electron configuration.

## Results and discussion

**Scheme 1:**
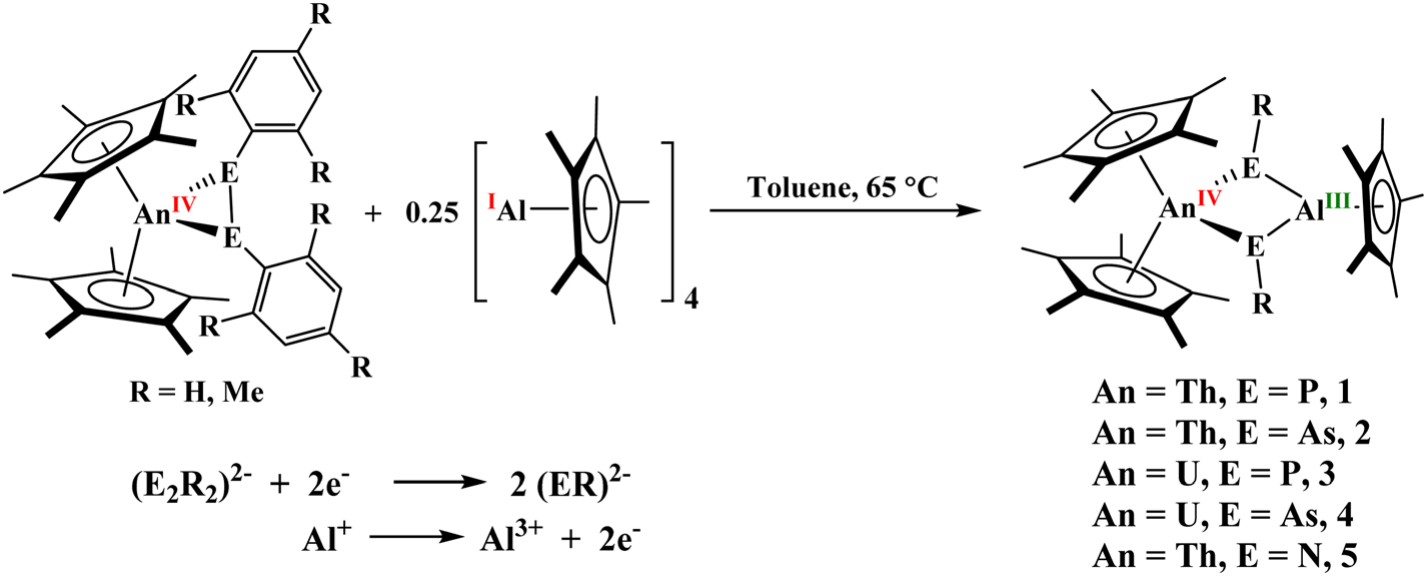
Synthesis of [(C_5_Me_5_)_2_An(*µ*_2_-ER)_2_Al(C_5_Me_5_)], An = Th, E = P, R = Mes, 1; An = Th, E = As, R = Mes, 2; An = U, E = P, R = Mes, 3; An = U, E = As, R = Mes, 4, An = Th, E = N, R = Ph, 5.

Treatment of [(C_5_Me_5_)_2_An(*η*^2^-E_2_R_2_)]^26*a*^ with 0.25 equivalents of [Al(C_5_Me_5_)]_4_ at 65 °C breaks the E–E bond to form the corresponding imide, phosphinidiide or arsinidiide complexes, [(C_5_Me_5_)_2_An(*µ*_2_-ER)_2_Al(C_5_Me_5_)], An = Th, E = P, R = Mes, 1; An = Th, E = As, R = Mes, 2; An = U, E = P, R = Mes, 3; An = U, E = As, R = Mes, 4, An = Th, E = N, R = Ph, 5, Scheme 1, Mes = 2,4,6-Me_3_C_6_H_2_. This redox reaction is summarized in Scheme 1, in which Al(i) is oxidized to Al(iii), while each (E_2_Mes_2_)^2−^ reduces to two [E(Mes)]^2−^. In each case, the actinide remains in the tetravalent oxidation state. The ^1^H NMR spectra of compounds 1 and 2 show resonances within the typical diamagnetic region, with the (C_5_Me_5_)^1−^ methyl signals appearing at 2.32 and 2.14 ppm. These values are slightly downfield compared to their respective analogous diphosphido and diarsenido complexes [(C_5_Me_5_)_2_An(*η*^2^-E_2_Mes_2_)], where E = P or As, both located at 1.90 ppm. This observed downfield shift is likely attributed to the incorporation of the Lewis acidic Al(iii) center within the heterobimetallic structure, which decreases the overall electron density at the thorium center relative to that in the parent diphosphido/diarsenido complexes. Further evidence for the change in electronic environment about the actinide metal center when Al(iii) is present was observed when a ∼1.0 V difference was observed in comparing the U(iv/iii) redox couple in the U(vi) compound, [(C_5_Me_5_)_2_U{=N(4-^i^PrOC_6_H_4_)}_2_], −2.30 V (*vs.* [(C_5_H_5_)_2_Fe^+/0^]) *versus* the U(iv)/Al(iii), [(C_5_Me_5_)_2_U{*µ*_2_-N(4-^i^PrOC_6_H_4_)}_2_Al(C_5_Me_5_)], −1.28 V (*vs.* [(C_5_H_5_)_2_Fe^+/0^]).^25^ A similar downfield shift in the resonance of the Cp^‡^ (Cp^‡^ = 1,4-di-*tert*-butylcyclopentadienyl) was seen in [Cp^‡^_2_ThCl(*µ*-H)_3_-AlC(SiMe_3_)_3_] upon incorporation of the Al(iii) center.^27^ The appearance of the singlet corresponding to 15 protons of (C_5_Me_5_)^1−^ associated with the Al(iii) center at 1.87 (for 1) and 1.77 (for 2) ppm confirms the presence of the AlCp* moiety in the heterobimetallic framework. The ^31^P NMR spectrum of 1 exhibits a single resonance at −6.83 ppm, upfield from that of the diphosphido complex (58.92 ppm).^26*a*^ In contrast to the diamagnetic complexes 1 and 2, the ^1^H NMR spectra of 3 and 4 showed paramagnetic behavior with (C_5_Me_5_)^1−^ resonances at 7.74 and 9.43 ppm, respectively, for uranium metallocene, while the (C_5_Me_5_)^1−^ resonances associated with the aluminum center appear at −7.25 and −6.71 ppm. The silent nature of compound 3 towards the ^31^P NMR indicates the direct connection of phosphorus to the paramagnetic U(iv) metal center. Owing to the significant paramagnetic broadening and extensive chemical shift dispersion caused by the unpaired electrons in these complexes, unambiguous observation and assignment of the signals to specific phosphorus and carbon atoms, respectively, proved challenging.

**Fig. 1:**
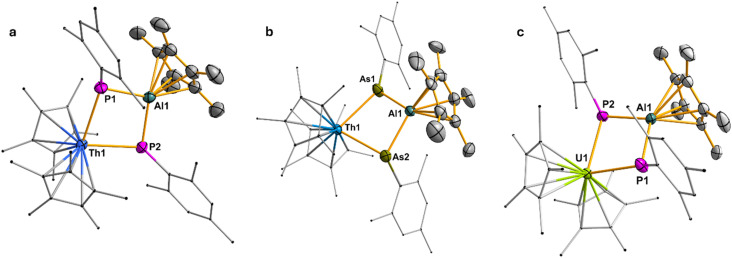
(a) Molecular structure of compound 1. All the hydrogen atoms are omitted for clarity. Displacement parameters are shown at 50% probability; selected interatomic distances [Å] and angles [°]: Th1–P1 2.8419(11), Th1–P2 2.7737(12), P1–Al1 2.3716(15), P2–Al1 2.3244(15), Th1–Al1 3.7140(9); P1–Th1–P2 77.67(4), Th1–P1–Al1 90.40(4), Th1–P2–Al1 93.10(4), P1–Al1–P2 97.17(5). (b) Molecular structure of compound 2. All the hydrogen atoms are omitted for clarity. Displacement parameters are shown at 50% probability; selected interatomic distances [Å] and angles [°]: Th1–As1 2.9815(10), Th1–As2 2.8462(11), As1–Al1 2.463(3), As2–Al1 2.376(3), Th1–Al1 3.822(9); As1–Th1–As2 77.61(3), Th1–As1–Al1 88.65(7), Th1–As2–Al1 93.67(4), As1–Al1–As2 98.03(10). (c) Molecular structure of compound 3. All the hydrogen atoms are omitted for clarity. Displacement parameters are shown at 50% probability; selected interatomic distances [Å] and angles [°]: U1–P1 2.7327(9), U1–P2 2.6952(9), P1–Al1 2.3290(13), P2–Al1 2.3124(13), U1–Al1 3.632(9); P1–U1–P2 78.37(3), U1–P1–Al1 91.33(4), U1–P2–Al1 92.65(4), P1–Al1–P2 95.27(5).

Compounds 1–4 are soluble in THF, diethyl ether, and toluene, and can be stored for prolonged periods at −20 °C under an inert nitrogen atmosphere. Notably, the phosphinidiide-bridged complexes 1 and 3 exhibit greater thermal stability compared to their arsinidiide analogs 2 and 4. This enhanced stability can be attributed to the comparatively harder nature of phosphorus relative to arsenic, which enables stronger interactions with the hard actinide centers, thereby affording more robust phosphinidiide-bridged complexes. The solid-state structures of complexes 1–3 were determined *via* single-crystal X-ray diffraction analysis (Fig. 1). All three complexes crystallize in the triclinic space group *P*1̄, with one molecule per asymmetric unit. In each case, the An(iv) metal center adopts a pseudo-tetrahedral geometry, coordinated by two Cp* ligands and two bridging phosphinidiide or arsinidiide ligands. The Al(iii) center is arranged in a trigonal planar fashion, and is bound to two bridging pnictinidiide ligands and one Cp* ligand coordinated in *η*^5^ fashion. In compound 3, significant shortening of the U–P bonds (∼0.071(12) Å) (U1–P1: 2.7327(9) Å; U1–P2: 2.6953(9) Å) is observed compared to the diphosphido precursor [(C_5_Me_5_)_2_U(*η*^2^-P_2_Mes_2_)], which shows U–P bond lengths of 2.7799(10) and 2.7903(10) Å (Table 1).^26*a*^ This contraction suggests a reduced electron density at the uranium center due to interaction with the Lewis acidic aluminum. In contrast, for the Th/Al systems in compounds 1 and 2, only one of the Th–E (*E* = P, As) bonds is shorter than those in the corresponding dipnictido precursors, while the other remains comparable. This indicates an uneven distribution of electron density across the Th–E–Al bridging units. In both complexes, one pnictogen center (P or As) exhibits a pyramidal geometry at the Th1–E1–Al1 linkage, with the total bond angles around P1 (320.45(18)°) and As1 (314.8(4)°) being close to 320° (Table 1). In contrast, the second pnictogen atom (E2) adopts an almost trigonal planar arrangement at the Th1–E2–Al1 unit, where the sum of bond angles around P2 and As2 is effectively 360°, indicating significant π-electron delocalization within the Th1–E2–Al1 fragment. This delocalization is supported by the shorter E2–Al1 and Th1–E2 bond lengths (P: 2.3244(15) and 2.7737(12) Å; As: 2.376(3) and 2.8462(11) Å) around the trigonal planar centre relative to those for the pyramidal centers (P: 2.3716(15) and 2.8419(11) Å; As: 2.463(3) and 2.9815(10) Å) (Table 1). In contrast, compound 3, featuring a U(iv) center, shows no evidence of such asymmetry. Both the phosphorus atoms P1 and P2 are symmetrically coordinated to U1, with U1–P1 and U1–P2 bond distances of 2.7327(9) and 2.6952(9) Å, respectively, leading to a balanced electron distribution around the U–P–Al–P moiety. Moreover, the sum of bond angles around the P1 and P2 centers also deviates slightly from the trigonal planarity, with 357.69(16)° and 343.51(17)°, respectively (Table 1).

**Table 1:** Selected bond lengths (Å) and angles (deg) of the An–E2–Al systems in 1, 2, and 3

	An1–E1	E1–Al1	An1–E2	E2–Al1	Σ_(bond angle)_(E1)	Σ_(bondangle)_(E2)
1	2.8419(11)	2.3716(15)	2.7737(12)	2.3244(15)	320.45(18)°	359.7(3)°
2	2.9815(10)	2.463(3)	2.8462(11)	2.376(3)	314.8(4)°	360.0(4)°
3	2.7327(9)	2.3290(13)	2.6952(9)	2.3124(13)	357.69(16)°	343.51(17)°

In compound 1, the Th1–P1 (2.8419(11) Å) bond length aligns well with Th–P single bonds observed in the Th(iv) diphosphido precursor [(C_5_Me_5_)_2_Th(*η*^2^-P_2_Mes_2_)] (2.8463(7) and 2.8322(6) Å).^26*a*^ In contrast, the noticeably shorter Th1–P2 bond (2.7737(12) Å) falls between the sums of covalent radii of a Th–P single bond (2.86 Å) and a double bond (2.45 Å), indicating significant delocalization of π electrons, contributing partial double bond character.^28^ This bond is comparable to that in the charge-separated Thorium(iv) complex [Th(Tren^TIPS^)(PH)][Na(12C4)_2_] (2.7584(18) Å), with Th=P double bond reported by Liddle and co-workers.^29^ A similar bond length disparity is seen in the P–Al distances: the P1–Al1 bond (2.3716(15) Å) is slightly longer than the P2–Al1 bond (2.3244(15) Å). These values are consistent with known P–Al single bonds in compounds such as [Trip_2_AlP(Ad)SiPh_3_] (2.342(2) Å),^30^ Trip = 2,4,6-^i^Pr_3_C_6_H_2_, Ad = adamantyl, and [MesTerP(^3*t*^CpAl)_2_] (2.3249(13) and 2.3304(14) Å), MesTer = 2,6-Mes_2_C_6_H_3_.^31,32^ In contrast, the uranium analog 3 shows symmetric P–Al bond lengths (P1–Al1: 2.3290(13) Å; P2–Al1: 2.3124(13) Å), reflecting a uniform electronic environment across the U–P_2_–Al core. In compound 2, the delocalization of π-electrons across one Th–As–Al bridge is even more pronounced. The Th1–As1 bond length (2.9815(10) Å) falls within the typical range for Th–As single bonds, as reported in several complexes including [{Th(*η*^5^-1,3-^*t*^Bu_2_C_5_H_3_)}_2_(*µ-η*^3^:*η*^3^-As_6_)] (2.913(2)–3.044(2) Å), [Th(*η*^5^-C_5_Me_5_)_2_(AsH-2,4,6-^i^Pr_3_C_6_H_2_)_2_] (3.0028(6) Å),^33^ [Th(Tren^TIPS^)(AsH_2_)] (3.065(3) Å),^34^ and [{Th(Tren^TIPS^)}_2_(*µ*-AsH)] (2.9619(6)/3.0286(6) Å).^35^ The As1–Al1 bond length of 2.463(3) Å closely matches those reported for Al–As single bonds, such as in (Mes*Al–AsPh)_3_ (2.430 Å),^36^ Mes* = 2,4,6-^*t*^Bu_3_C_6_H_2_, and [Et_2_AlAs(SiMe_3_)_2_]_2_ (2.539(2) Å).^37^

Interestingly, in the second Th1–As2–Al1 fragment of compound 2, where the arsenic atom adopts a trigonal planar geometry (with a sum of bond angles around E2 = 360.0(4)°), both Th1–As2 and As2–Al1 bond lengths are significantly shorter, 2.8462(11) Å and 2.376(3) Å, respectively (Table 1). These shorter distances imply substantial π-electron delocalization within this unit. The Th1–As2 bond lies between the covalent single bond sum for Th–As (2.96 Å) and the estimated double bond value (2.57 Å), and is marginally longer than the polarized Th=As double bond distance (2.8063(14) Å) observed in the dithorium arsenide complex [{Th(Tren^TIPS^)}_2_(*µ*-As)][K(15C5)_2_].^35^ Simultaneously, the As2–Al1 bond (2.376(3) Å) approaches the sum of covalent double bond radii for As=Al (2.27 Å),^38^ and is comparable to the experimentally observed Al=As bond in the arsaalumene [DippTerAs=AlCp*] (2.3084(4) Å), DippTer = 2,6-(2,6-^i^Pr_2_C_6_H_3_)C_6_H_3_. Moreover on comparison with the As–Al single bond length observed for the cyclic diarsadialanes [Cp*Al(*µ*-AsAr)]_2_ (Ar = Dipp (2.4106(8) and 2.4462(16) Å); Trip (2.4160(5) and 2.4445(5)Å)) by Hering-Junghans and workers the bond length is shorter, supporting the presence of partial multiple bond character.^32,37^

**Fig. 2:**
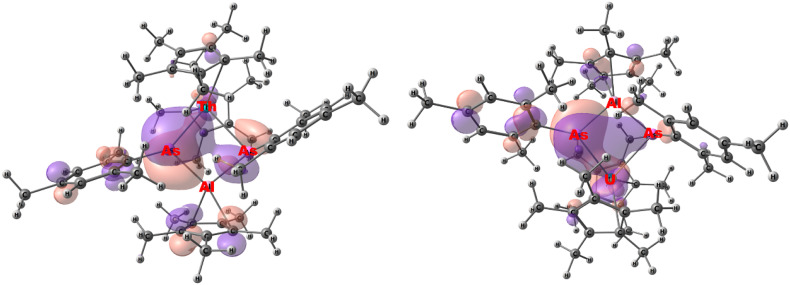
Molecular orbitals (HOMO − 1) of compounds 2 (Th–As–Al) and 4 (U–As–Al).

DFT calculations (B3PW91 functional including dispersion corrections) were undertaken on complexes 1–4 to analyze the bonding situation in those four complexes. Each complex was optimized, and the global minimum was confirmed by frequency calculations. The optimized geometries of complexes 1–3, for which a solid-state structure was obtained, compare well with the experimental ones (see Tables S3, S10, and S17 in the SI). In particular, the An–E distances with An = Th, U, and E = P, As, are well reproduced, as well as the unsymmetrical Th–E or symmetrical U–P coordination. The bonding in the four complexes was analyzed using Natural Bonding Orbitals (NBO) and Quantum Theory of Atoms in Molecule (QTAIM) methods, Tables 2 and 3. For the phosphorus-based complexes 1 and 3, NBO analysis indicates a difference in the bonding situation. Indeed, for the unsymmetrical coordination of Th, one Th=P double bond and one Th–P single bond with a lone pair (HOMO of the system) are found (see Table S8 in the SI), while two U=P double bonds, fully delocalized, are found in the symmetrical coordination case (see Table S22 in the SI). In both cases (Th and U), the bonds are strongly polarized toward P (76–84%) and implies df orbitals on either Th or U that participate in bonding (Fig. 2).

**Table 2:** Wiberg bond index (WBI) from NBO analysis, electron density, *ρ*(*r*), and Laplacian of the electron density, ∇^2^·*ρ*(*r*), from QTAIM analysis for complexes 1 and 2

	1, An = Th, E = P WBI	1, An = Th, E = P *ρ*(*r*)	1, An = Th, E = P ∇^2^·*ρ*(*r*)	2, An = Th, E = As WBI	2, An = Th, E = As *ρ*(*r*)	2, An = Th, E = As ∇^2^·*ρ*(*r*)
An–E1	1.26	0.101	0.211	1.29	0.054	0.052
An–E2	1.07	0.101	0.212	1.08	0.053	0.019
Al–E1	0.69	0.079	0.531	0.75	0.056	0.108
Al–E2	0.63	0.079	0.530	0.67	0.053	0.067

**Table 3:** Wiberg bond index (WBI) from NBO analysis, electron density, *ρ*(*r*), and Laplacian of the electron density, ∇^2^·*ρ*(*r*), from QTAIM analysis for complexes 3 and 4

	3, An = U, E = P WBI	3, An = U, E = P *ρ*(*r*)	3, An = U, E = P, ∇^2^·*ρ*(*r*)	4, An = U, E = As WBI	4, An = U, E = As *ρ*(*r*)	4, An = U, E = As ∇^2^·*ρ*(*r*)
An–E1	1.26	0.067	0.068	1.26	0.060	0.065
An–E2	1.04	0.060	0.046	0.82	0.049	0.022
Al–E1	0.71	0.056	0.140	0.79	0.055	0.100
Al–E2	0.71	0.059	0.120	0.75	0.053	0.069

Interestingly, the QTAIM analysis indicates in both cases two An–P Bond Critical Points (BCP), with similar electron density properties (see Tables S9 and S26 in SI) so that the bonding would be similar between the metal center and the two phosphorus. This can be explained by the presence of the Ring Critical Point (RCP) in the An–P–Al–P plane that indicates electron delocalization within the four-member ring. In both complexes, the P–Al bond is described as a single bond strongly polarized toward P (80%), which explains the Al–P WBI of 0.7. It is interesting to note that at the second-order donor–acceptor level, delocalization from the Al–P bond onto the empty df orbital is observed in the case of Th but not in the case of U, in line with the difference in bonding situation in complexes 1 and 3. The bonding situation appears to be similar in complexes 2 and 4 (see SI).

The Wiberg Bond Index (WBI) of 1.26/1.07 in 1 for the Th–P bonds is similar to 1.30 found for the bridging phosphinidiide complex, [(C_5_Me_5_)_2_Th{P(H)Tipp}(PTipp)K].^39^ Terminal thorium phosphinidene complexes have been shown to have WBI ∼1.67.^29^ Bridging arsinidene (arsinidiide) complexes of thorium have WBI of 1.30 in [(C_5_Me_5_)_2_Th{As(H)Tipp}(AsTipp)K] and 1.09 in [Th(Tren^TIPS^)(*µ*-AsH)K(15C5)], Tren^TIPS^ = N(CH_2_CH_2_NSi^i^Pr_3_)_3_,^35^ which compares well to the 1.29/1.08 observed in 2. In contrast, the WBI for the Al–P and Al–As bonds in 1 and 2 are significantly less than the 1.47 and 1.46 in (C_5_Me_5_)Al = E(Ter^Dipp^), E = P, As, respectively, Ter^Dipp^ = 2,6-(2,4,6-^i^Pr_3_C_6_H_2_)C_6_H_3_.^31,40^ In the QTAIM calculations, the electron density at the BCP, *ρ*(*r*), along with its Laplacian, ∇^2^·*ρ*(*r*), is a measure of the degree of covalent bonding and hence a larger BCP indicates increase in covalent bonding character.^41^ These BCPs are used to characterize the nature of the chemical interaction between atoms and can be interpreted in the following way: When *ρ*(*r*) > 0.2 a.u. with ∇^2^·*ρ*(*r*) < 0 is indicative of a covalent interaction, while *ρ*(*r*) < 0.1 a.u. with ∇^2^·*ρ*(*r*) > 0 represents an ionic or electrostatic interaction. The ellipticity parameter, *ε*(*r*), presents a spherical spread of electron density and therefore a *ε*(*r*) ∼0 indicates σ-bonding while *ε*(*r*) > 0 shows π-bonding with asymmetric electron density distribution. Examining the data in Table 2, the Th–P shows *ρ*(*r*) of 0.101 with a higher Laplacian of 0.211, thus this can be interpreted as a highly polarized bond with multiple bonding character. Shown in Table S9, the *ε*(*r*) for complex 1 are 0.223/0.222 for Th–P and 0.097/0.095 for Al–P, which can be compared to 0.40 in [Th(Tren^TIPS^)(*µ*-PH)]^1−^.^29^ while complex 2 has *ε*(*r*) values of 0.451/0.158 for Th–As and 0.133/0.101 for Al–As.

The WBI for the U–P, 3, and U–As, 4, bonds, Table 3, of 1.26/1.04 and 1.26/0.82 is less than that observed in terminal U=P and U=As bonds at 1.92 (or 1.61 for bridging phosphinidiide) and 1.62, respectively.^42,43^ However, 3 and 4 both have higher WBI than anticipated for U–E single bonds which are observed ∼0.84 and 0.69 for P and As, respectively. Similar to the Th compounds, the ellipticity parameter is greater than zero, indicating that there is multiple bonding character in both 3 and 4 as well.

**Scheme 2:**
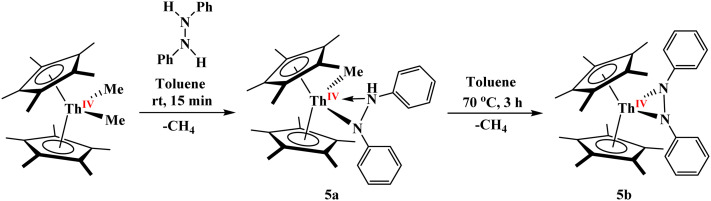
Synthesis of 5a and 5b.

Following the successful isolation of the previously unknown bridging phosphinidiide/arsinidiide heterobimetallic complexes 1–3, we sought to extend this synthetic methodology to access the analogous imido-bridged complexes of uranium/thorium and aluminum. Akin to the preparation of diphosphido complexes [(C_5_Me_5_)_2_An(*η*^2^-P_2_Mes_2_)] through the protonolysis of [(C_5_Me_5_)_2_AnMe_2_] using dimesityldiphosphane^26*a*^ the reactivity of [(C_5_Me_5_)_2_ThMe_2_] with diphenylhydrazine was investigated. Treatment of [(C_5_Me_5_)_2_ThMe_2_] with an equimolar amount of diphenylhydrazine in toluene at room temperature afforded the monoamido complex [(C_5_Me_5_)_2_Th(CH_3_)(*η*^2^-PhNN(H)Ph)], 5a, in 78% yield *via* selective protonolysis of a single methyl group, Scheme 2. Subsequent heating of 5a in toluene at 70 °C for 3 hours induced a second deprotonation, yielding the diamido complex [(C_5_Me_5_)_2_Th(*η*^2^-PhNNPh)(thf)], 5b, in 80% yield, with methane as a byproduct, Scheme 2. While the reaction is done in toluene, THF is added during crystallization as the unsolvated complex will not yield crystals suitable for single-crystal X-ray crystallographic analysis. Alternatively, compound 5b could be obtained directly by heating an equimolar mixture of [(C_5_Me_5_)_2_ThMe_2_] and diphenylhydrazine in toluene under the same thermal conditions. Such diamido complexes for group IV elements (Ti and Zr) are reported, but to the best of our knowledge, complex 5b represents the first reported example of a Th(iv) diamido species.^44–46^ Complex 5b reacts rapidly with water, yielding the previously reported thorium *µ*-oxo dimer, [{(C_5_Me_5_)_2_Th(*η*^2^-PhN(H)NPh)}_2_(*µ*-O)], which resists further deprotonation.^47^ Solution-state characterization of compounds 5a and 5b was performed using multinuclear NMR spectroscopy. In the case of 5a, the methyl protons of the Cp* ligands appear as two distinct singlets at 1.86 and 1.99 ppm, reflecting the asymmetric coordination environment around the thorium center. Conversely, the diamido complex 5b displays a single singlet at 1.77 ppm for the Cp* methyl protons, indicating a more symmetric ligand environment. The methyl and NH protons in 5a appear as a singlet and a broad singlet at −0.08 and 4.86 ppm, respectively. The asymmetry in 5a is further supported by the presence of five distinct aromatic signals from the diphenyl groups, in contrast to three signals observed for the symmetrically coordinated 5b.

**Fig. 3:**
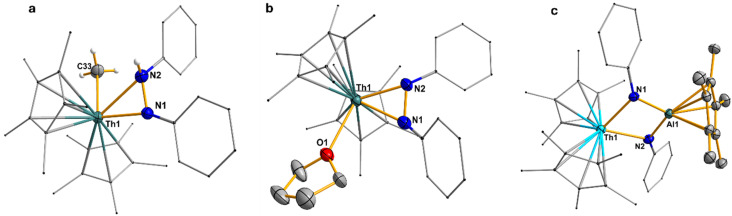
(a) Molecular structure of compound 5a. All the hydrogen atoms except for NH and methyl protons are omitted for clarity. Displacement parameters are shown at 50% probability; selected interatomic distances [Å] and angles [°]: Th1–C33: 2.491(3), Th1–N1: 2.348(3), Th11–N2: 2.594(3), N2–N1: 1.430(4); N1–Th1–N2: 33.16(8), N1–Th1–C33: 106.91(11), N2–Th1–C33: 78.18(11). (b) Molecular structure of compound 5b. All the hydrogen atoms are omitted for clarity. Displacement parameters are shown at 50% probability; selected interatomic distances [Å] and angles [°]: Th1–O1: 2.543(3), Th1–N1: 2.338(3), Th1–N2: 2.272(3), N2–N1: 1.469(4); N1–Th1–N2: 37.12(11), N1–Th1–O1: 86.48(10), N2–Th1–O1: 120.81(11). (c) Molecular structure of compound 5. All the hydrogen atoms are omitted for clarity. Displacement parameters are shown at 50% probability; selected interatomic distances [Å] and angles [°]: Th1–N1: 2.281(2), Th1–N2: 2.302(2), N1–Al1: 1.821(2), N2–Al1: 1.816(2); N1–Th1–N2: 69.72(8), Th1–N1–Al1: 88.65(7), Th1–N2–Al1: 98.45(9), N1–Al1–N2: 92.14(10).

The solid-state characterization of compounds 5a and 5b was performed using single-crystal X-ray diffraction (Fig. 3) and IR spectroscopy. Both compounds crystallize in the triclinic space group *P*1̄, with 5a containing one molecule and 5b containing two molecules per asymmetric unit, Fig. 3. In 5a, the thorium center is five-coordinate, bound to two nitrogen atoms from a diphenylhydrazide ligand, a methyl group, and two Cp* ligands. The diphenylhydrazide adopts an *η*^2^-binding mode through both nitrogen atoms, although the coordination is notably asymmetric. The deprotonated nitrogen features a nearly linear C–N–Th bond angle of 156.7(2)° and a relatively short Th–N bond length of 2.348(3) Å. Conversely, the protonated nitrogen atom displays a more typical trigonal planar geometry with a C–N–Th angle of 123.5(2)° and a longer Th–N bond of 2.594(3) Å, illustrating the structural impact of protonation. The N–H stretching band observed at 3320 cm^−1^ further confirms the existence of an N–H bond in compound 5a.

In compound 5b, the thorium center adopts a distorted tetrahedral geometry, coordinated by a hydrazide dianion and two Cp* ligands. The hydrazide binds in an *η*^2^-mode with unequal Th–N distances of 2.258(3) and 2.342(3) Å. Despite this asymmetry in the solid state, the NMR spectrum shows a single resonance at 1.77 ppm for the 30 protons of two Cp* ligands and three signals corresponding to the phenyl groups, indicating a symmetrical species in solution. The asymmetry observed in the crystal structure may be attributed to the coordination of a THF molecule. The N–N bond length in 5b is 1.468(5) Å, consistent with a single bond but longer than those reported for related titanium and zirconium hydrazide complexes, [Cp_2_M(N_2_Ph_2_)], where the N–N distances are 1.334 Å (Ti) and 1.434(4) Å (Zr).^44–46^ This elongation suggests a more polarized N–N bond, likely due to the strong electron-withdrawing effect of the highly Lewis acidic thorium center. We note that the original preparation of the bis(imido) complex, [(C_5_Me_5_)_2_U(=NPh)_2_], through reaction of [(C_5_Me_5_)_2_UMeCl] with [LiNPhN(H)Ph], was proposed to go through a hydrazide intermediate,^48^ but was not isolated. However, the formation of 5b shows that this is a potential intermediate, but due to the limited oxidation states of thorium, the N–N bond cannot be cleaved. In contrast, oxidation with U(iv) to U(vi) is possible, allowing the bis(imido) to form.

After isolating the hitherto unknown thorium(iv) diamido complex 5b containing an N–N bond, its reactivity with Al(i)Cp* in toluene was explored, leading to the formation of the heterobimetallic bridging imido complex [(C_5_Me_5_)_2_Th(*µ*_2_-NPh)_2_Al(C_5_Me_5_)] (5) in 85% yield, Scheme 1. Compound 5 is soluble in THF, toluene, and diethyl ether, and remains stable at room temperature under an inert atmosphere. The ^1^H NMR spectrum at room temperature indicates a diamagnetic species, showing Cp* resonances at 1.95 and 1.92 ppm corresponding to the thorium and aluminum centers, respectively. The molecular structure of 5 was elucidated *via* single-crystal X-ray diffraction, revealing that the complex crystallizes in the monoclinic space group *P*2_1_/*n* with one molecule per asymmetric unit, Fig. 3. Structurally, the complex features two bridging imido ligands connecting the Th(iv) and Al(iii) centers. The thorium center adopts a distorted tetrahedral geometry coordinated by two Cp* ligands and two nitrogen atoms from the bridging imidos, while the aluminum center is trigonal planar, coordinated by one Cp* ligand and the same two imido bridges. Interestingly, unlike the heavier bridging pnictogen analogs 1 and 2, compound 5, featuring a bridging imido ligand, exhibits greater electron delocalization across the Th–N1–Al1–N2 metallocycle. The Th–N bond lengths of 2.281(2) and 2.302(2) Å are comparable to those in the precursor complex 5b. Similarly, the Al1–N1 (1.821(2) Å) and Al1–N2 (1.816(2) Å) distances are consistent with those observed in the previously reported uranium analog [(C_5_Me_5_)_2_U[*µ*_2_-N(4-^i^PrOC_6_H_4_)]Al(C_5_Me_5_)] (1.838(4) and 1.827(4) Å).^25^ Consistent with the XRD findings, NBO and QTAIM analyses further support symmetrical coordination in imido-bridged complex 5, featuring a nearly planar four-membered An–N–Al–N ring with symmetrical bonding features (see SI). Building on this observation, we next turned our attention to synthesizing the analogous uranium(iv)/aluminum(iii) bridging imido complex.

**Scheme 3:**
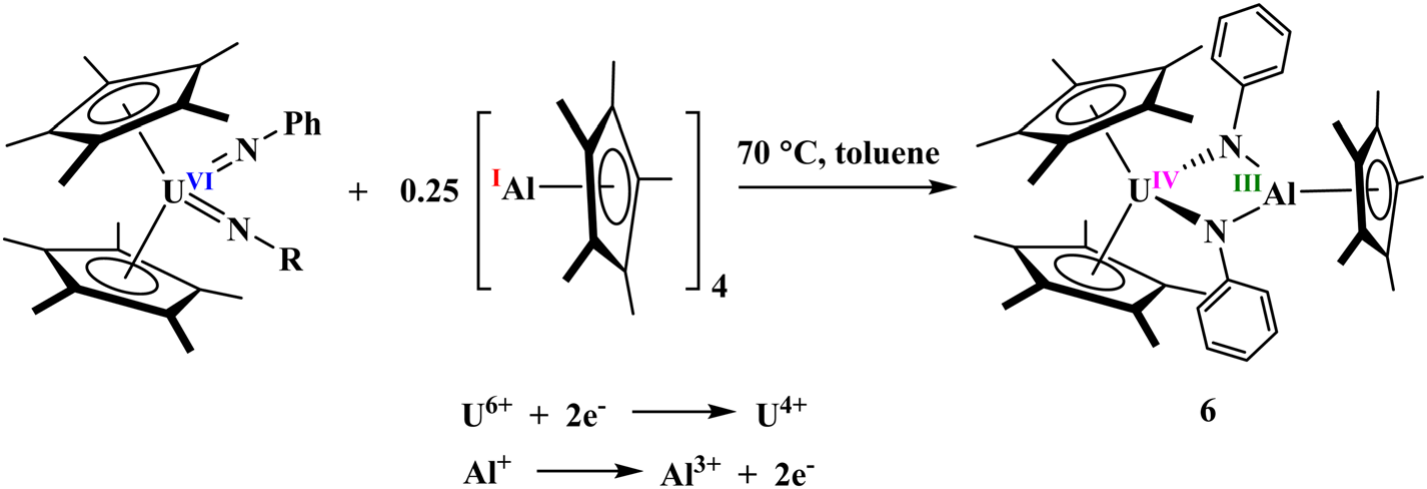
Synthesis of complex 6 from U(vi) precursors.

In contrast to the reaction of [(C_5_Me_5_)ThMe_2_] with diphenylhydrazine, which affords the monoamido complex [(C_5_Me_5_)_2_Th(CH_3_)(*η*^2^-PhNN(H)Ph)], the analogous uranium complex [(C_5_Me_5_)_2_UMe_2_] reacts with an equimolar amount of diphenyl- or mesitylhydrazine to yield the uranium(vi) bis(imido) species [(C_5_Me_5_)_2_U(=NR)_2_] (R = Ph, Mes). To this end, a stoichiometric reaction was carried out between the uranium(vi) bis(imido) complex [(C_5_Me_5_)_2_U(=NMes)_2_], bearing mesityl substituents on the nitrogen atoms and 0.25 equivalents of [Al(C_5_Me_5_)]_4_ in toluene at 70 °C for 15 minutes, aiming for a two-electron reduction of the U(vi) center. However, this attempt to generate the desired product was unsuccessful, and the preliminary XRD studies show evidence for the formation of a C–H activated product A (see SI for the XRD data). This transformation probably proceeds with the elimination of an equimolar amount of Cp*H and a 1,3-hydrogen shift from a mesityl methyl group to the adjacent imido nitrogen. This outcome is likely due to the spatial proximity between the bulky mesityl substituent and the AlCp* reagent within the U(vi) bis(imido) framework. C–H bond activation of a *tert*-butyl-substituted imido complex has been previously reported.^49^ Attempts to suppress this C–H activation by conducting the reaction at room temperature resulted in no observable reaction, presumably because the temperature above 70 °C is necessary to promote the dissociation of the (AlCp*)_4_ tetramer. Thus, we explored the use of a less bulky phenyl group in place of the mesityl substituent and did not fully characterize the complex. In this case, the reaction of the U(vi) bis(imido) complex [(C_5_Me_5_)_2_U(=NPh)_2_]^48,50^ with Al(i)Cp* under identical conditions (in toluene at 70 °C) led to the successful formation of the bridging imido U(iv)/Al(iii) heterobimetallic complex, [(C_5_Me_5_)_2_U(*µ*_2_-NPh)_2_Al(C_5_Me_5_)], 6, through a two-electron redox reaction involving U and Al centers, Scheme 3.

**Fig. 4:**
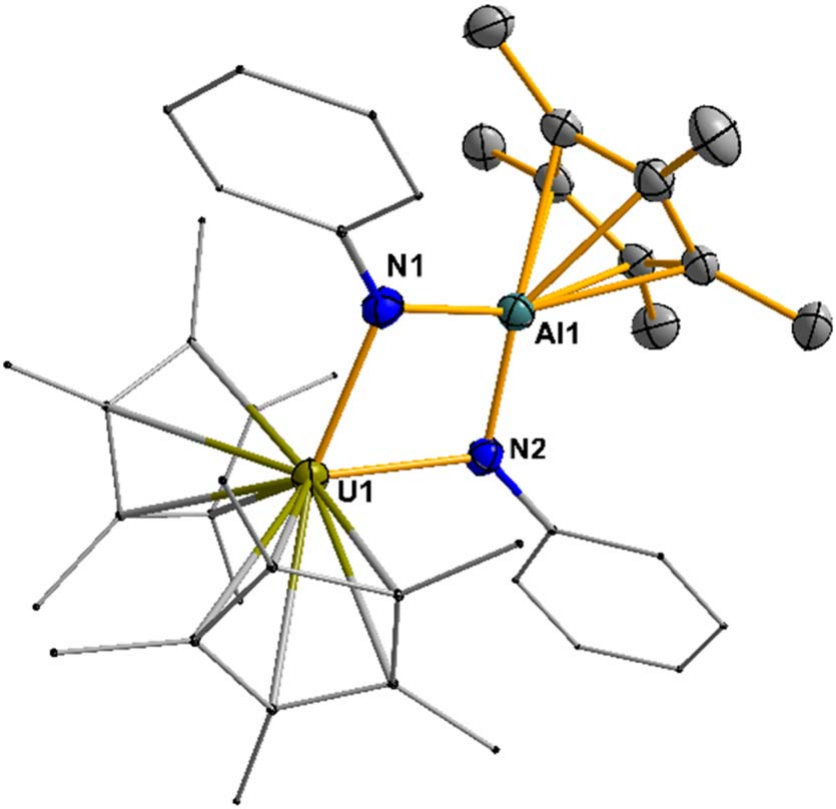
Molecular structure of compound 6. All the hydrogen atoms are omitted for clarity. Displacement parameters are shown at 50% probability; selected interatomic distances [Å] and angles [°]: U1–N1 2.244(3), U1–N2 2.225(2), N1–Al1 1.836(3), N2–Al1 1.833(3), N1–Al1 3.632(9); N1–U1–N2 71.75(9), U1–N1–Al1 97.80(11), U1–N2–Al1 98.57(11), N1–Al1–N2 91.07(12).

Compound 6 is soluble in common organic solvents such as toluene, THF, and diethyl ether, and remains stable at room temperature when stored under an inert dinitrogen atmosphere. In solution, 6 was characterized by multinuclear NMR spectroscopy, while solid-state characterization was carried out using single-crystal X-ray diffraction and infrared spectroscopy. The paramagnetic nature of compound 6 is evident in its ^1^H NMR spectrum, where the aromatic protons of the phenyl groups appear as three distinct singlets in the paramagnetic region (−2.25, −3.77, and −25.34 ppm). The methyl resonances corresponding to the U-Cp* and the Al-Cp* ligand appear as two singlets at 3.42 and 0.79 ppm, respectively. Single-crystal XRD analysis revealed that compound 6 crystallizes in the monoclinic space group *P*2_1_/*n* with one molecule per asymmetric unit. Structurally, the complex comprises edge-sharing pseudo-tetrahedral U(iv) and trigonal planar Al(iii) centers, bridged by two imido ligands, Fig. 4. The overall molecule exhibits non-crystallographic *C*_s_ symmetry, with a mirror plane passing through both metal centers and the Cp* ligands, symmetrically relating the two (NPh)^2−^ bridges. The U–N bond lengths (2.244(3) Å and 2.225(2) Å) are significantly longer than the U=N double bonds in the U(vi) bis(imido) precursor.^48^ Still, they are consistent with reported U(iv) with terminal amido ligands^49^ and other U(iv) bridging imido complexes.^50,51^ The Al–N bond length of 1.836(3) and 1.833(3) Å are comparable to other bridging aluminum(iii) imido and amido complexes. For example, the Al–N bond lengths in [(C_5_Me_5_)Al(NSi^*t*^Bu_3_)]_2_ are 1.836(2) and 1.840(2) Å,^13,52^ while those in [(C_5_H_2_^*t*^Bu_3_)Al(*µ*^2^-NPh)]_2_ are 1.809(2) and 1.827(2) Å.^53^ These findings highlight that the less bulky phenyl substituents on the imido ligands afford sufficient spatial accommodation to enable the formation of the bridging imido heterobimetallic complex directly from U(vi) bis(imido) precursors.

To better understand the origin of the difference in bonding between Th and U, calculations were carried out on the nitrogen analogs that were experimentally obtained with a phenyl substituent. In these two complexes, 5 (Th) and 6 (U), the coordination is symmetrical for both Th and U (see SI) with a planar four-member An–N–Al–N ring, and the NBO and QTAIM indicate symmetrical bonding features, Table 4. Therefore, the optimization of putative Th and U complexes with a mesityl substituent on the nitrogen was undertaken. In both cases, the An–N–Al–N ring is no longer planar, indicating that the four-member ring is too sterically constrained with the smaller nitrogen atoms to allow planarity, while it is possible with the phenyl. Thus, this provides a reasonable explanation for the unsuccessful formation of the mesityl derivative, where C–H bond activation is observed. In complexes 1–4, the larger P and As can accommodate the sterically bulkier mesityl groups. However, we note that the two bonds in the calculated mesityl-substituted bridging imido complexes are symmetric, indicating that the bonding situation is like that of the phenyl-substituted complexes that were isolated (5 and 6). This also indicates that the steric properties of the mesityl *versus* phenyl are responsible for the trigonal planar *versus* pyramidal geometry about phosphorus and arsenic in complexes 1 and 2.

**Table 4:** Wiberg bond index (WBI) from NBO analysis and electron density, *ρ*(*r*), from QTAIM analysis for complexes 5 and 6

	5, An = Th, WBI	5, *ρ*(*r*)	5, ∇^2^·*ρ*(*r*)	6, An = U, WBI	6, *ρ*(*r*)	6, ∇^2^·*ρ*(*r*)
An–N1	0.77	0.101	0.212	0.94	0.111	0.262
An–N2	0.77	0.101	0.211	0.94	0.111	0.262
Al–N1	0.35	0.079	0.530	0.35	0.079	0.522
Al–N2	0.35	0.079	0.531	0.35	0.079	0.522

To further study the electron delocalization in the An–X–Al–X moiety, Nucleus Independent Chemical Shift (NICS) calculations were carried out.^54^ The NICS(0), that accounts for σ + π aromaticity, are found to be small in the three thorium complexes with values of −3.7 ppm in As, −4.1 ppm in P, and −4.5 ppm in N, in line with small aromaticity (mainly σ). However, the values for the uranium complexes are much larger with values of −8.0 ppm in As, −18.7 ppm in P, and −25.6 ppm in N, in line with both σ + π aromaticity.^55^ These values can be compared to organic molecules such as benzene which has NISC(0) value of −24.5 ppm or pyridine of −25.0 ppm.^56^ This aromaticity arises due to the presence of six electrons in the An–X–Al–X moiety. In the case of Th, the pnictogen must donate all six electrons, hence the multiple bonding character observed as described earlier, however U(iv) is a 5f^2^ ground state electron configuration and hence these two electrons contribute to the π-system and thus the electron density is delocalized over U–X–Al–X. The NICS(0) values decrease from nitrogen to arsenic, indicating a decrease of the π delocalization for the heaviest pnictogen elements which is presumably a result of the bond distances in the An–X–Al–X moiety. Aromaticity in f element complexes is rare,^57–61^ and controversial,^61–63^ but has been previously observed in a similar manner where U(iv) contributes its 5f electrons to the overall aromatic bonding character.^64^ We must acknowledge that these computational results may simply be due to dispersion effects. Indeed, without dispersion correction, the asymmetry observed in the crystal structure is not observed. However, even without dispersion effects, the structure is more symmetrical, indicating that the aromaticity does hold in these cases.

## Conclusion

To summarize, the reactivity of dipnictido actinide complexes, [(C_5_Me_5_)_2_An(*η*^2^-E_2_R_2_)] (An = Th, U; E = P, As; R = 2,4,6-Me_3_C_6_H_2_); E = N, R = Ph, with 0.25 equiv. of [Al(C_5_Me_5_)]_4_ demonstrates selective E–E bond cleavage to afford the heterobimetallic complexes [(C_5_Me_5_)_2_An(*µ*_2_-ER)_2_Al(C_5_Me_5_)]. Solid-state structural analyses, complemented by DFT calculations, provide key insights into the bonding characteristics of these heterobimetallic systems. DFT calculations indicate that aromatic bonding is observed in both thorium and uranium complexes in all six compounds isolated. These results highlight the contrasting electronic and bonding features of thorium and uranium in cooperative E–E bond activation and underscore the potential of actinide–main group combinations for mediating complex bond cleavage and transformation processes, as well as the bonding situations in heavier main group elements are not the same as their first-row counterparts.

## Author contributions

PM and RJW were responsible for the experimental portion, SPK was responsible for the crystallography, and GW and LM were responsible for the computational parts. JRW supervised the project and was responsible for obtaining funding. All helped with writing the manuscript.

## Conflicts of interest

There are no conflicts to declare.

## Supplementary Material

SC-017-D5SC09143H-s001

SC-017-D5SC09143H-s002

## Data Availability

All supporting documents including experimental and computational details, spectra, and structural data are accessible free of charge. The data supporting this article have been included as part of the supplementary information (SI). Supplementary information: details of experimental methods, characterization data, and theoretical calculation details. See DOI: https://doi.org/10.1039/d5sc09143h. CCDC 2501693–2501700 contain the supplementary crystallographic data for this paper.^65*a*–*h*^

## References

[cit1] Nief F. (1998). Coord. Chem. Rev..

[cit2] Liddle S. T. (2015). Angew. Chem., Int. Ed..

[cit3] Natrajan L. S., Swinburne A. N., Andrews M. B., Randall S., Heath S. L. (2014). Coord. Chem. Rev..

[cit4] Jones M. B., Gaunt A. J. (2013). Chem. Rev..

[cit5] Alexander V. (1995). ChemInform.

[cit6] Ephritikhine M. (2013). Organometallics.

[cit7] Rookes T. M., Wildman E. P., Balázs G., Gardner B. M., Wooles A. J., Gregson M., Tuna F., Scheer M., Liddle S. T. (2018). Angew. Chem., Int. Ed..

[cit8] Dagorne S., Wehmschulte R. (2018). ChemCatChem.

[cit9] Allan C. J. and MacDonald C. L. B., Comprehensive Inorganic Chemistry II (Second Edition), From Elements to Applications, 2013, vol. 1, pp. 485–566

[cit10] Roesky P. W. (2009). Dalton Trans..

[cit11] Nagendran S., Roesky H. W. (2008). Organometallics.

[cit12] Hobson K., Carmalt C. J., Bakewell C. (2020). Chem. Sci..

[cit13] Schulz S., Voigt A., Roesky H. W., Häming L., Herbst-Irmer R. (1996). Organometallics.

[cit14] Gemel C., Steinke T., Cokoja M., Kempter A., Fischer R. A. (2004). Eur. J. Inorg. Chem..

[cit15] Schulz S., Roesky H. W., Koch H. J., Sheldrick G. M., Stalke D., Kuhn A. (1993). Angew. Chem., Int. Ed..

[cit16] Dohmeier C., Robl C., Tacke M., Schnöckel H. (1991). Angew. Chem., Int. Ed..

[cit17] Fischer R. A., Weiß J. (1999). Angew. Chem., Int. Ed..

[cit18] Liu Y., Li J., Ma X., Yang Z., Roesky H. W. (2018). Coord. Chem. Rev..

[cit19] Schulz S., Kuczkowski A., Schuchmann D., Flörke U., Nieger M. (2006). Organometallics.

[cit20] Minasian S. G., Krinsky J. L., Rinehart J. D., Copping R., Tyliszczak T., Janousch M., Shuh D. K., Arnold J. (2009). J. Am. Chem. Soc..

[cit21] Sugita K., Yamashita M. (2020). Chem. – Eur. J..

[cit22] Minasian S. G., Krinsky J. L., Williams V. A., Arnold J. (2008). J. Am. Chem. Soc..

[cit23] Krinsky J. L., Minasian S. G., Arnold J. (2011). Inorg. Chem..

[cit24] Gamer M. T., Roesky P. W., Konchenko S. N., Nava P., Ahlrichs R. (2006). Angew. Chem., Int. Ed..

[cit25] Ward R. J., Del Rosal I., Chirdon D. N., Kelley S. P., Tarlton M. L., Maron L., Walensky J. R. (2020). Inorg. Chem..

[cit26] Tarlton M. L., Fajen O. J., Kelley S. P., Kerridge A., Malcomson T., Morrison T. L., Shores M. P., Xhani X., Walensky J. R. (2021). Inorg. Chem..

[cit27] Altman A. B., Brown A. C., Rao G., Lohrey T. D., Britt R. D., Maron L., Minasian S. G., Shuh D. K., Arnold J. (2018). Chem. Sci..

[cit28] Pyykkö P. (2015). J. Phys. Chem. A.

[cit29] Wildman E. P., Balázs G., Wooles A. J., Scheer M., Liddle S. T. (2016). Nat. Commun..

[cit30] Wehmschulte R. J., Ruhlandt-Senge K., Power P. P. (1994). Inorg. Chem..

[cit31] Fischer M., Nees S., Kupfer T., Goettel J. T., Braunschweig H., Hering-Junghans C. (2021). J. Am. Chem. Soc..

[cit32] Nees S., Fantuzzi F., Wellnitz T., Fischer M., Siewert J., Goettel J. T., Hofmann A., Härterich M., Braunschweig H., Hering-Junghans C. (2021). Angew. Chem., Int. Ed..

[cit33] Scherer O. J., Schulze J., Wolmershker G. (1994). J. Organomet. Chem..

[cit34] Behrle A. C., Walensky J. R. (2016). Dalton Trans..

[cit35] Wildman E. P., Balázs G., Wooles A. J., Scheer M., Liddle S. T. (2017). Nat. Commun..

[cit36] Wehmschulte R. J., Power P. P. (1996). J. Am. Chem. Soc..

[cit37] Wells R. L., McPhail A. T., Speer T. M. (1992). Organometallics.

[cit38] Pyykkö P., Atsumi M. (2009). Chem. – Eur. J..

[cit39] Vilanova S. P., Alayoglu P., Heidarian M., Huang P., Walensky J. R. (2017). Chem. – Eur. J..

[cit40] Dankert F., Hering-Junghans C. (2022). Chem. Commun..

[cit41] Kerridge A. (2017). Chem. Commun..

[cit42] Gardner B. M., Balázs G., Scheer M., Tuna F., McInnes E. J. L., McMaster J., Lewis W., Blake A. J., Liddle S. T. (2015). Nat. Chem..

[cit43] Gardner B. M., Balázs G., Scheer M., Tuna F., McInnes E. J. L., McMaster J., Lewis W., Blake A. J., Liddle S. T. (2014). Angew. Chem., Int. Ed..

[cit44] Walsh P. J., Hollander F. J., Bergman R. G. (1990). J. Am. Chem. Soc..

[cit45] Walsh P. J., Hollander F. J., Bergman R. G. (1992). J. Organomet. Chem..

[cit46] Fochi G., Floriani C., Bart J. C. J., Giunchi G. (1983). J. Chem. Soc., Dalton Trans..

[cit47] Mahawar P., Kelley S. P., Walensky J. R. (2025). Dalton Trans..

[cit48] Arney D. S. J., Burns C. J., Smith D. C. (1992). J. Am. Chem. Soc..

[cit49] Berthet J. C., Ephritikhine M. (1998). Coord. Chem. Rev..

[cit50] Burns C. J., Smith W. H., Huffman J. C., Sattelberger A. P. (1990). J. Am. Chem. Soc..

[cit51] Broderick E. M., Gutzwiller N. P., Diaconescu P. L. (2010). Organometallics.

[cit52] Schulz S., Thomas F., Priesmann W. M., Nieger M. (2006). Organometallics.

[cit53] Hofmann A., Tröster T., Kupfer T., Braunschweig H. (2019). Chem. Sci..

[cit54] Chen Z., Wannere C. S., Corminboeuf C., Puchta R., Von P., Schleyer R. (2005). Chem. Rev..

[cit55] Furukawa S., Fujita M., Kanatomi Y., Minoura M., Hatanaka M., Morokuma K., Ishimura K., Saito M. (2018). Commun. Chem..

[cit56] Nees S., Kupfer T., Hofmann A., Braunschweig H. (2020). Angew. Chem., Int. Ed..

[cit57] Tomeček J., Liddle S. T., Kaltsoyannis N. (2023). ChemPhysChem.

[cit58] Sheng W., Rajeshkumar T., Zhao Y., Maron L., Zhu C. (2024). J. Am. Chem. Soc..

[cit59] Eulenstein A. R., Franzke Y. J., Lichtenberger N., Wilson R. J., Deubner H. L., Kraus F., Clérac R., Weigend F., Dehnen S. (2021). Nat. Chem..

[cit60] Lin X., Mo Y. (2022). Angew. Chem., Int. Ed.Angew. Chem., Int. Ed..

[cit61] Boronski J. T., Seed J. A., Hunger D., Woodward A. W., van Slageren J., Wooles A. J., Natrajan L. S., Kaltsoyannis N., Liddle S. T. (2021). Nature.

[cit62] Szczepanik D. W. (2023). RSC Adv..

[cit63] Szczepanik D. W. (2022). Angew. Chem., Int. Ed..

[cit64] Pagano J. K., Xie J., Erickson K. A., Cope S. K., Scott B. L., Wu R., Waterman R., Morris D. E., Yang P., Gagliardi L., Kiplinger J. L. (2020). Nature.

[cit65] (a) CCDC 2501693: Experimental Crystal Structure Determination, 2026, 10.5517/ccdc.csd.cc2pz6sp

