# Supplementary material for: Unusual bonding situations in Th(iv) and U(iv)–Al(iii) pnictogen complexes
Source: Chem Sci. 2026 Mar 18;17(18):9158–67. doi: 10.1039/d5sc09143h (PMC12997793; doi:10.1039/d5sc09143h)
Supplement: SC-017-D5SC09143H-s001 [file SC-017-D5SC09143H-s001.pdf]

Supplementary Information (SI) for Chemical Science.
This journal is © The Royal Society of Chemistry 2026

Unusual Bonding Situations in Th(IV) and U(IV)-Al(III) Pnictogen Complexes

Pritam Mahawar,[1] Ganping Wang,[2] Robert J. Ward,[1] Steven P. Kelley,[1] Laurent Maron,[2]* and Justin R. Walensky[1]*

[1] Department of Chemistry, University of Missouri, Columbia, MO 65211 USA

[2] Université de Toulouse, INSA Toulouse, CNRS, LPCNO, 31077 Toulouse, France

Contents

1. Experimental Characterization Data 1

Synthesis of [$(C_5Me_5)_2Th\{\mu_2\text{-}P(2,4,6\text{-}Me\text{-}C_6H_2)\}_2Al(C_5Me_5)$] (**1**). 2

Synthesis of [$(C_5Me_5)_2Th\{\mu_2\text{-}As(2,4,6\text{-}Me\text{-}C_6H_2)\}_2Al(C_5Me_5)$] (**2**). 5

Synthesis of [$(C_5Me_5)_2U\{\mu_2\text{-}P(2,4,6\text{-}Me\text{-}C_6H_2)\}_2Al(C_5Me_5)$] (**3**) 8

Synthesis of [$(C_5Me_5)_2U\{\mu_2\text{-}As(2,4,6\text{-}Me\text{-}C_6H_2)\}_2Al(C_5Me_5)$] (**4**) 10

Synthesis of [$(C_5Me_5)_2Th(CH_3)(\eta^2\text{-}N(H)(C_6H_5)N(C_6H_5)$] (**5a**). 11

Synthesis of [$(C_5Me_5)_2Th\{\eta^2\text{-}N(C_6H_5)\}_2(thf)$] (**5b**). 14

Synthesis of [$(C_5Me_5)_2Th\{\mu^2\text{-}N(C_6H_5)\}_2Al(C_5Me_5)$] (**5**). 16

Synthesis of [$(C_5Me_5)_2U\{\mu_2\text{-}N(C_6H_5)\}_2Al(C_5Me_5)$] (**6**). 19

2. Crystallographic Data Collection and Structure Determination. 21

3. UV-Visible spectrum of compounds **1**,**3**, **5**, and **6**. 28

4. Computational details 32

5. References 79

1. Experimental Characterization Data

General considerations. Unless specified, all reactions were performed under an inert atmosphere of nitrogen (N_2) using standard glovebox and Schlenk techniques. All solvents used in the synthesis were dried over molecular sieves and alumina in the MBRAUN solvent purification system. Toluene was further dried over sodium benzophenone ketyl radical and vacuum distillation. [$(C_5Me_5)_2ThMe_2$][2] and [$(C_5Me_5)_2UMe_2$],[2] [$(C_5Me_5)_2U(=NPh)_2$][3] dimestitylphosphane,[4] mesitylarsine,[5] [$(C_5Me_5)_2Th(\eta^2\text{-}P_2Mes_2)$],[6] [$(C_5Me_5)_2Th(\eta^2\text{-}As_2Mes_2)$],[6] [$(C_5Me_5)_2U(\eta^2\text{-}P_2Mes_2)$],[6] [$(C_5Me_5)_2U(\eta^2\text{-}As_2Mes_2)$],[6] and [$Al(C_5Me_5)]_4$,[7] were prepared following the reported literature procedure. 1,2-Diphenylhydrazine was used as received from Sigma Aldrich. All NMR spectra were recorded in deuterated benzene (C_6D_6) on either a 600

MHz, 500 MHz, or 400 MHz Bruker Advance spectrometer. ^{1}H, $^{13}C\{^{1}H\}$, and ^{31}P NMR shifts are reported in parts per million referenced internally to residue solvent impurities. Deuterated benzene was degassed with a three-cycle freeze-pump-thaw and dried over 4 Å molecular sieves for at least 48 hours before use. All infrared spectra were recorded as potassium bromide (KBr) pellets on a Nicolet Summit Pro FT-IR spectrometer.

Synthesis of $[(C_5Me_5)_2Th\{\mu_2\text{-}P(2,4,6\text{-}Me\text{-}C_6H_2)\}_2Al(C_5Me_5)]$ (1).

A J. Young NMR tube was charged with 50 mg (0.062 mmol) of $[(C_5Me_5)_2Th(\eta^2\text{-}P_2Mes_2)]$, 11.02 mg (0.017 mmol) $[(C_5Me_5)Al]_4$, and ca. 0.6 mL of benzene-d_6. The sealed NMR tube was shaken vigorously and heated at 70 °C for 1.5 h to observe a colour change from dark brown to orange. The NMR recorded after 1.5 h showed the complete consumption of $[(C_5Me_5)_2Th(\eta^2\text{-}P_2Mes_2)]$ with the formation of compound $(C_5Me_5)_2Th\{\mu_2\text{-}[P(2,4,6\text{-}Me\text{-}C_6H_2)]_2\}Al(C_5Me_5)$ (**1**). The J. Young NMR tube was then taken inside the glove box, filtered, and the filtrate was collected in a scintillation vial. All volatiles were removed under reduced pressure, and the resulting orangish crude powder was extracted with diethyl ether (2 × 5 mL) to obtain an orangish brown amorphous solid. Further purification of compound **1** was achieved through crystallization using a solvent mixture of ether and pentane at -20 °C, yielding 46 mg (0.047 mmol, 77%) of orangish crystals.

^{1}H NMR (C_6D_6, 600 MHz, 298 K): δ 7.04 (s, 4H, *m*-H), 2.99 (s, 12H, *o*-CH_3), 2.32 (s, 6H, *p*-CH_3), 2.14 (s, 30H, ThC_5Me_5), 1.87 (s, 15H, AlC_5Me_5) ppm.

^{13}C NMR (C_6D_6, 151 MHz, 298 K): 141.8, 133.8, 128.3, 127.9 (ArMes), 127.2 (AlC_5Me_5), 117.2 (ThC_5Me_5), 27.6 (o-Me), 20.9 (p-Me), 12.3 (ThC_5Me_5), 11.6 (AlC_5Me_5) ppm.

^{31}P NMR (C_6D_6, 101 MHz, 298 K): -6.83 ppm.

IR (KBr, cm^{-1}): 2935 (m), 2896 (s), 2853 (m), 2723 (s), 2093 (m), 1599 (w), 1549 (w), 1456 (s), 1375 (s), 1261 (w), 1173 (w), 1046 (w), 1021 (s), 845 (s), 804 (w), 704 (w), 602 (w), 543 (w).

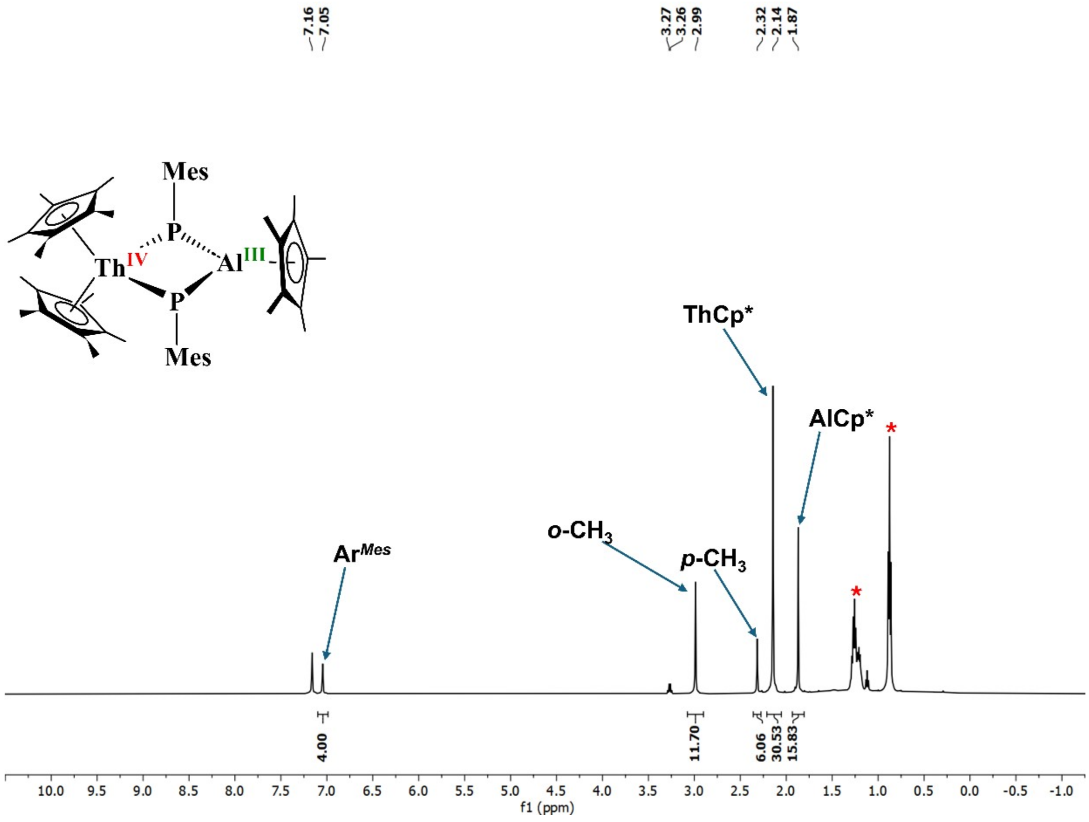

Figure S1. ^{1}H NMR (600 MHz, C_6D_6) of compound **1**. *n-pentane

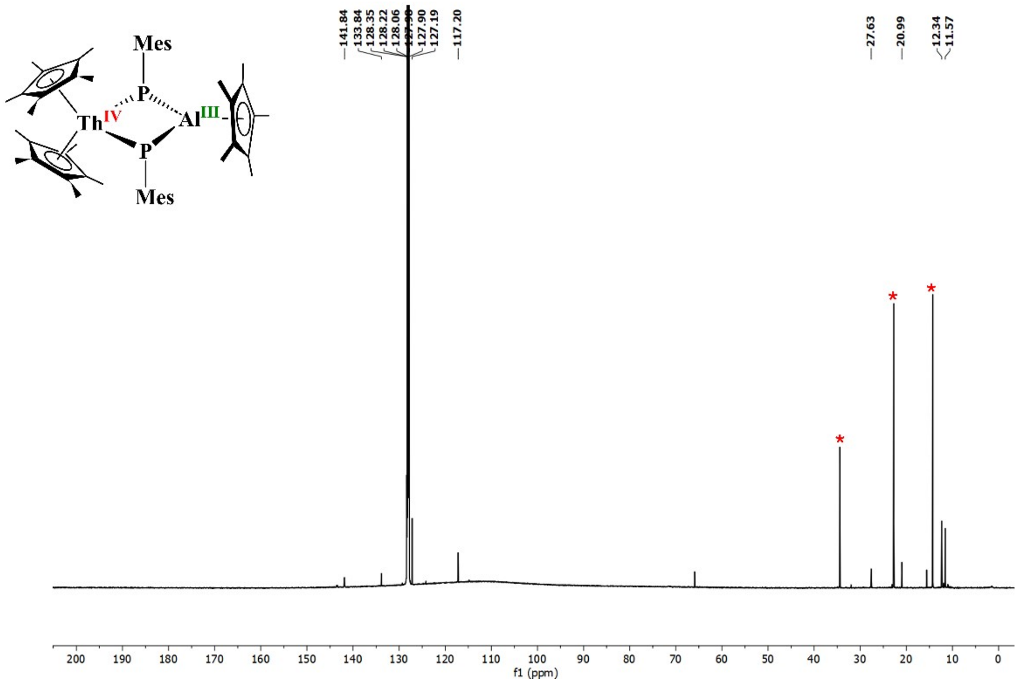

Figure S2. ^{13}C NMR (150 MHz, C_6D_6) of compound **1**. *n-pentane

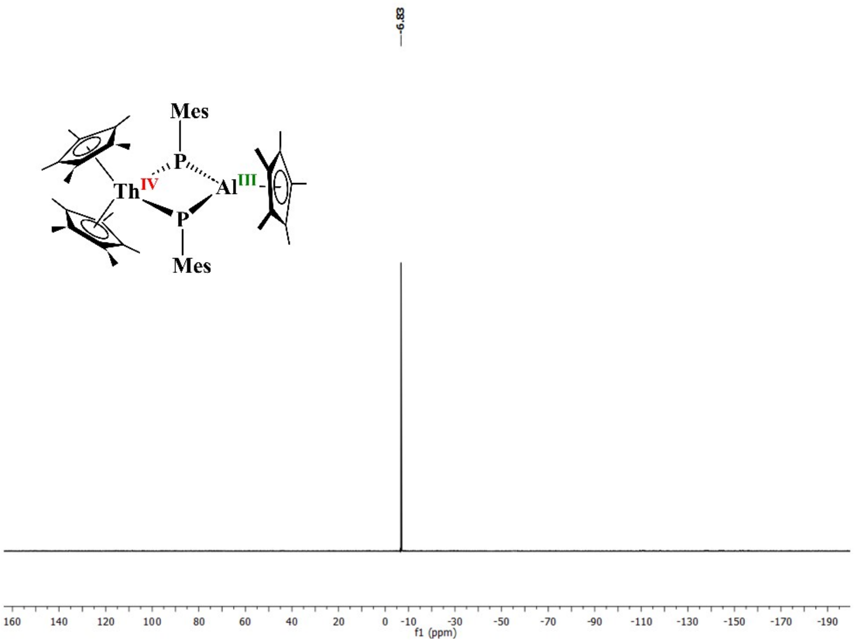

Figure S3. ^{31}P NMR (101 MHz, C_6D_6) of compound **1**.

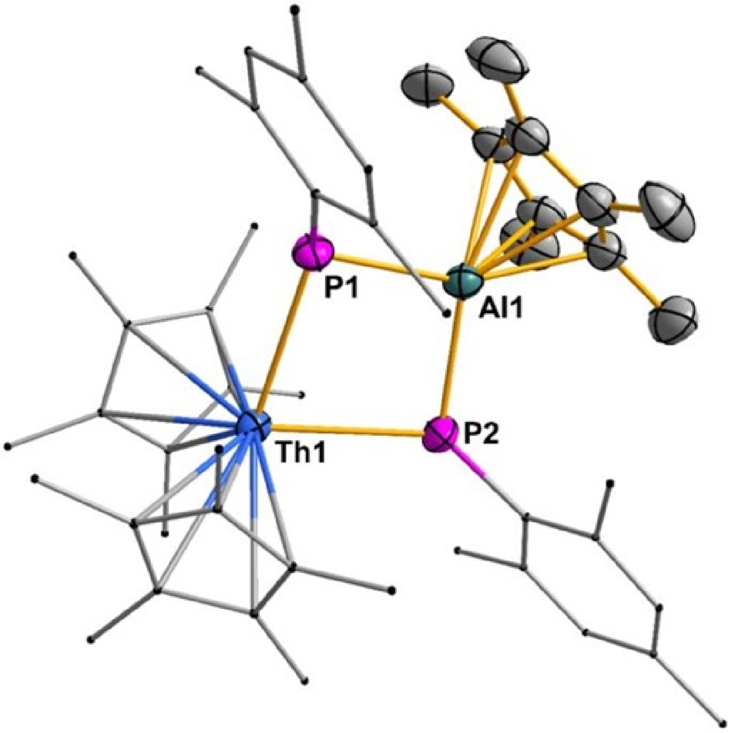

Figure S4. Molecular structure of compound **1**. All the hydrogen atoms are omitted for clarity. Displacement parameters are shown at 50% probability; Selected interatomic distances [Å] and angles [°]: Th1–P1: 2.8419(11), Th1–P2: 2.7737(12), P1–Al1: 2.3716(15), P2–Al1: 2.3244(15), Th1–Al1: 3.7140(9); P1–Th1–P2: 77.67(4), Th1–P1–Al1: 90.40(4), Th1–P2–Al1: 93.10(4), P1- Al1-P2: 97.17(5).

Synthesis of $[(C_5Me_5)_2Th\{\mu_2\text{-}As(2,4,6\text{-}Me\text{-}C_6H_2)\}_2Al(C_5Me_5)]$ (2).

A J. Young NMR tube was charged with 50 mg (0.056 mmol) of $[(C_5Me_5)_2Th(\eta^2\text{-}As_2Mes_2)]$, 10.00 mg (0.015 mmol) $[(C_5Me_5)Al]_4$, and ca. 0.6 mL of benzene-d_6. The sealed NMR tube was shaken vigorously and heated for 2 h at 70 °C to observe a colour change from greenish brown to orangish red. The NMR recorded after 1.5 h showed the complete consumption of $[(C_5Me_5)_2Th(\eta^2\text{-}As_2Mes_2)]$ with the formation of compound $[(C_5Me_5)_2Th\{\mu^2\text{-}As(2,4,6\text{-}Me\text{-}C_6H_2)\}_2Al(C_5Me_5)]$ (**2**). The J. Young NMR tube was then taken inside the glove box, filtered, and the filtrate was collected in a scintillation vial. All volatiles were removed under reduced pressure, and the resulting orangish-red crude powder was extracted with diethyl ether (2 × 5 mL) to obtain an orangish brown amorphous solid. Further purification of compound **2** was achieved through crystallization using a solvent mixture of ether and pentane at -20 °C, yielding 48 mg (0.045 mmol, 81%) of orangish red crystals.

^{1}H NMR (C_6D_6, 600 MHz, 298 K): δ 7.05 (s, 4H, m-H), 2.99 (s, 12H, o-CH_3), 2.81 (s, 6H, p- CH_3), 2.14 (s, 30H, ThC_5Me_5), 1.77 (s, 15H, AlC_5Me_5) ppm.

^{13}C NMR (C_6D_6, 151 MHz, 298 K): 141.7, 133.7, 127.9, 127.3 (*Ar*Mes), 127.0 (Al*C*$_5$Me$_5$), 117.0 (Th*C*$_5$Me$_5$), 27.5 (o-*Me*), 20.8 (p-*Me*), 12.7 (ThC$_5$*Me*$_5$), 11.4 (AlC$_5$*Me*$_5$) ppm.

IR (KBr, cm^{-1}): 2942 (s), 2916 (s), 2821 (s), 2722 (w), 2369 (w), 2308 (w), 2090 (w), 1627 (w), 1585 (w), 1456 (m), 1377 (m), 1252 (m), 1085 (m), 1010 (s), 846 (w), 835 (w), 815 (w), 640 (w).

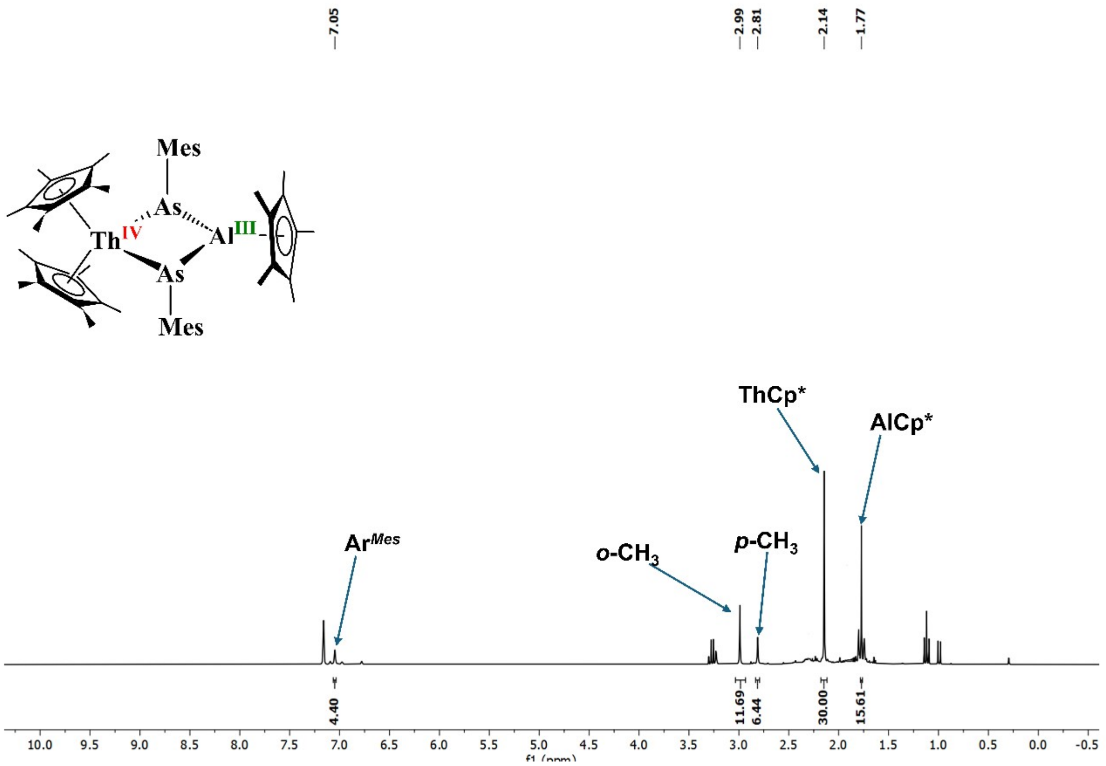

Figure S5. ^{1}H NMR (600 MHz, C_6D_6) of compound **2**.

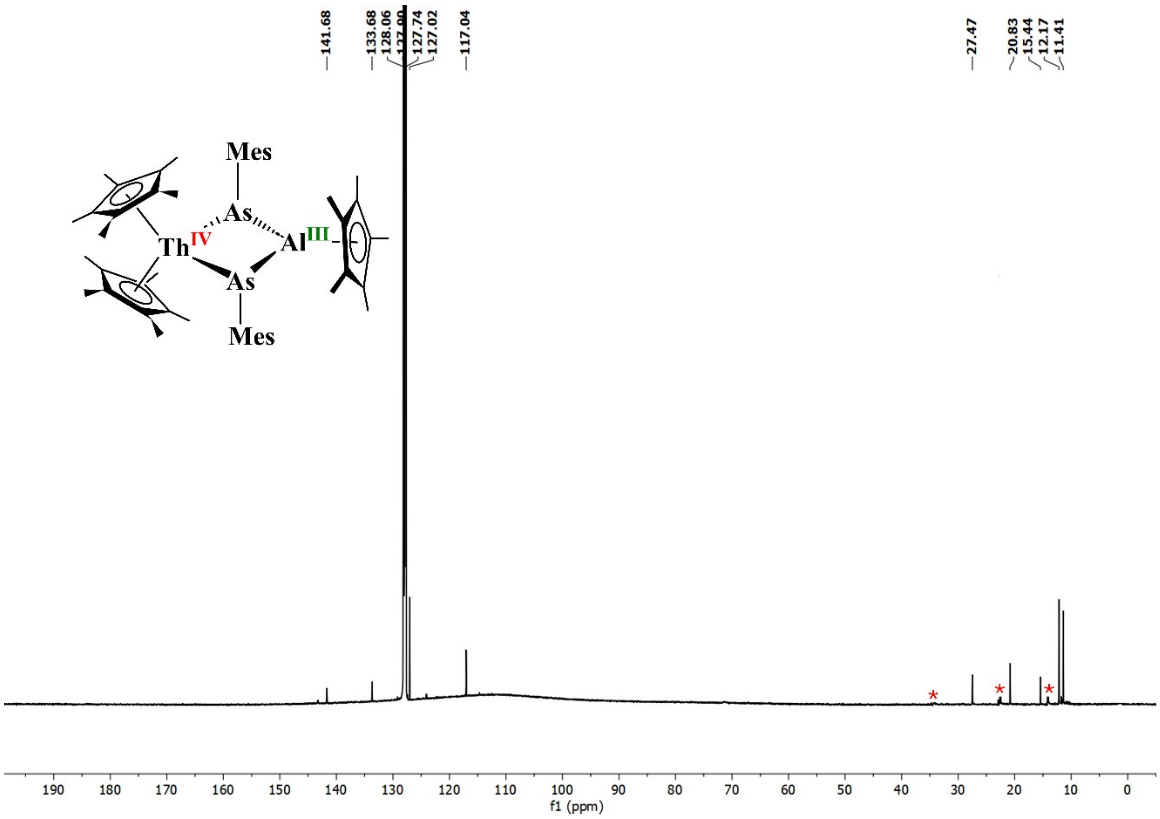

Figure S6. ^{13}C NMR (150 MHz, C_6D_6) of compound **2**. *n-pentane

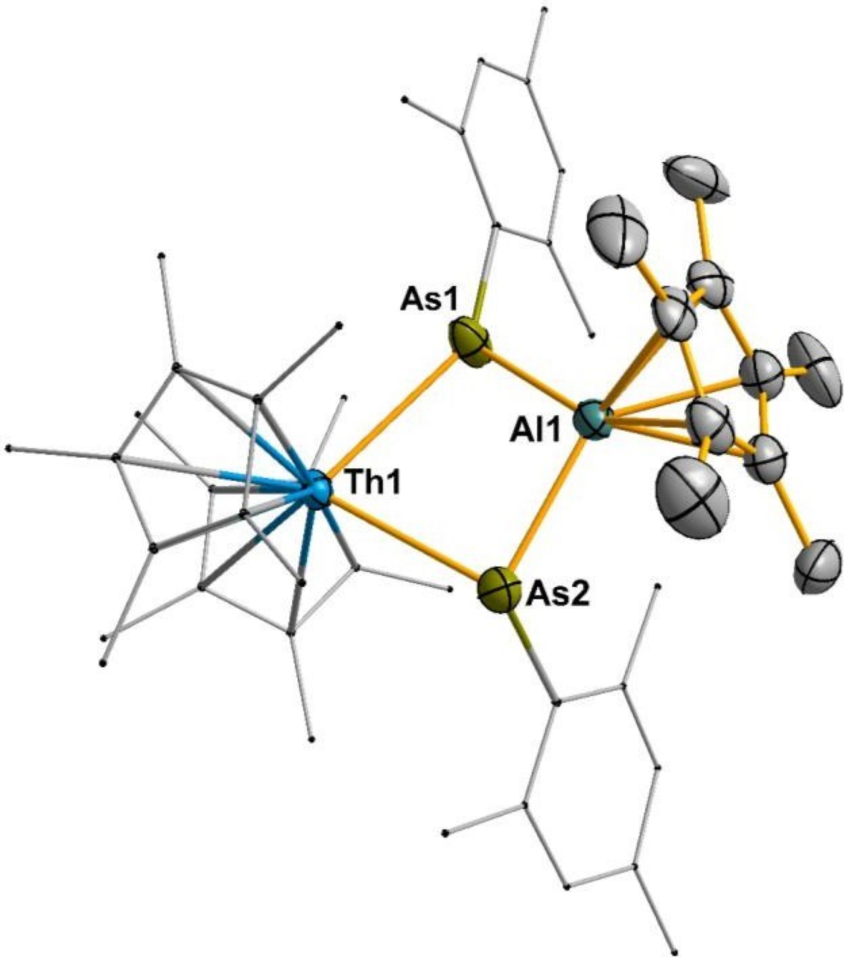

Figure S7. Molecular structure of compound **2**. All the hydrogen atoms are omitted for clarity. Displacement parameters are shown at 50% probability; Selected interatomic distances [Å] and angles [°]: Th1–As1: 2.9815(10), Th1–As2: 2.8462(11), As1–Al1: 2.463(3), As2–Al1: 2.376(3), Th1–Al1: 3.822(9); As1–Th1–As2: 77.61(3), Th1–As1–Al1: 88.65(7), Th1–As2–Al1: 93.67(4), As1-Al1-As2: 98.03(10).

Synthesis of [(C_5Me_5)$_2$U{μ_2-P(2,4,6-Me-C_6H_2)}$_2$Al(C_5Me_5)] (3).

A J. Young NMR tube was charged with 50 mg (0.061 mmol) of [(C_5Me_5)$_2$U(η^2-P_2Mes_2)], 11.02 mg (0.017 mmol) [(C_5Me_5)Al]$_4$, and ca. 0.6 mL of benzene-d_6. The sealed NMR tube was shaken vigorously and heated for 2 h at 70 °C to observe a colour change from black to brown. The NMR recorded after 2 h showed the complete consumption of [(C_5Me_5)$_2$U(η^2-P_2Mes_2)] with the formation of compound [(C_5Me_5)$_2$U{μ^2-P(2,4,6-Me-C_6H_2)}$_2$Al(C_5Me_5)] (**3**). The J. Young NMR tube was then taken inside the glove box, filtered, and the filtrate was collected in a scintillation vial. All the volatiles were removed under reduced pressure, and the resulting crude black powder was extracted with diethyl ether (2 x 5 mL) to obtain a brownish black powder. Further purification of compound **3** was achieved through crystallization using a solvent mixture of ether and pentane at -20 °C, yielding 50 mg (0.051 mmol, 83%) of brown crystalline material.

^{1}H NMR (C_6D_6, 600 MHz, 298 K): δ 7.74 (bs, 30H, UC_5*Me_5*), 6.80 (bs, 4H, m-*H*), 3.82 (s, 6H, p-C*H_3*), -7.25 (s, 15H, AlC_5*Me_5*).

IR (KBr, cm^{-1}): 2953 (s), 2900 (s), 2855 (s), 2725 (w), 2090 (m), 1712 (w), 1600 (w), 1550 (w), 1456 (s), 1375 (m), 1261 (m), 1091 (m), 1022 (s), 845 (s), 802 (m), 705 (w), 684 (w), 611 (w), 543 (w).

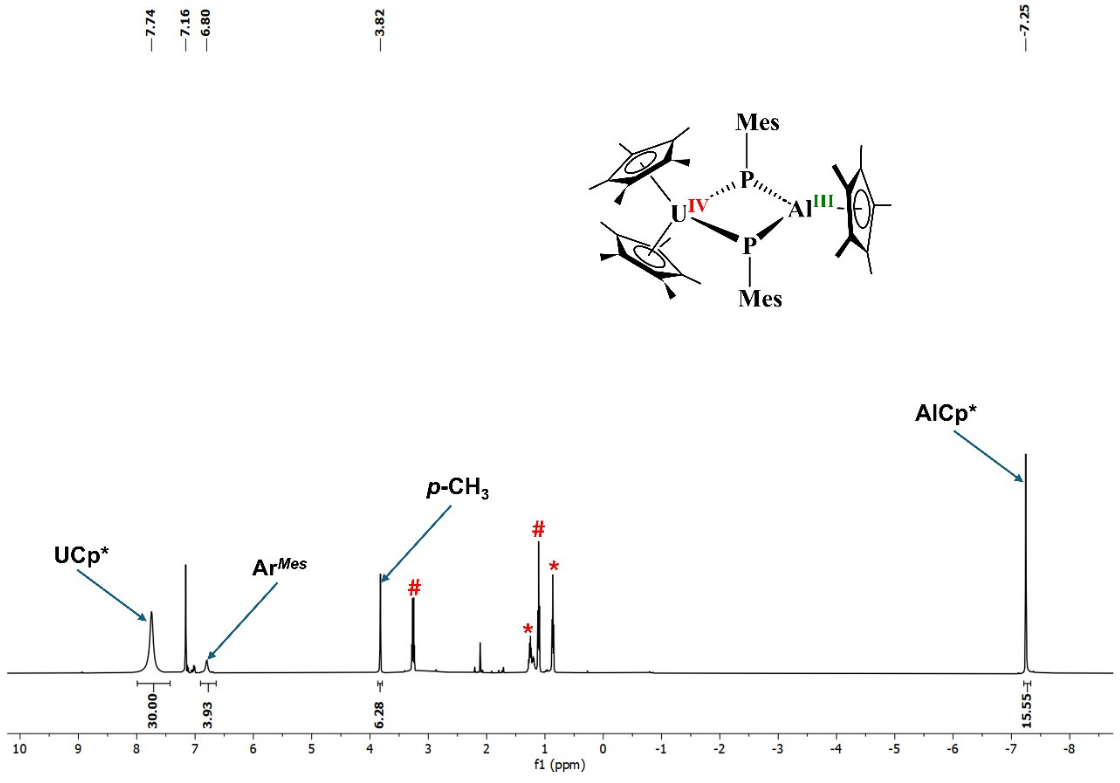

Figure S8. ^{1}H NMR (600 MHz, C6D6) of compound **3**. *n-pentane,#Diethylether

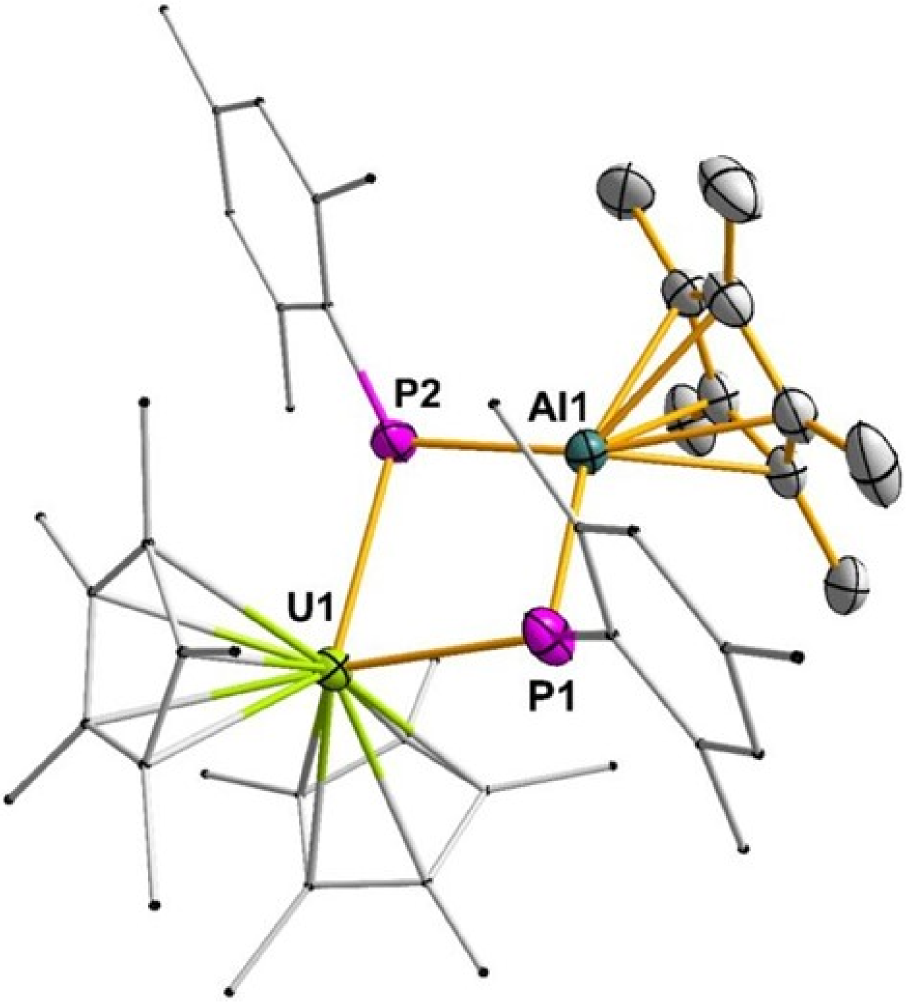

Figure S9. Molecular structure of compound **3**. All the hydrogen atoms are omitted for clarity. Displacement parameters are shown at 50% probability; Selected interatomic distances [Å] and angles [°]: U1–P1: 2.7327(9), U1–P2: 2.6952(9), P1–Al1: 2.3290(13), P2–Al1: 2.3124(13), U1–Al1: 3.632(9); P1–U1–P2: 78.37(3), U1–P1–Al1: 91.33(4), U1–P2–Al1: 92.65(4), P1-Al1-P2: 95.27(5).

Synthesis of [(C_5Me_5)$_2$U{μ_2-As(2,4,6-Me-C_6H_2)}$_2$Al(C_5Me_5)] (4).

A J. Young NMR tube was charged with 50 mg (0.055 mmol) of [(C_5Me_5)$_2$U(η^2-As_2Mes_2)], 9.07 mg (0.014 mmol) [(C_5Me_5)Al]$_4$, and ca. 0.5 mL of benzene-d_6. The sealed NMR tube was shaken vigorously and heated for 2 hours at 70 °C to observe a colour change from black to a greenish-brown colour. The NMR recorded after 2 h showed the complete consumption of [(C_5Me_5)$_2$U(η^2-As_2Mes_2)] with the formation of compound [(C_5Me_5)$_2$U{μ_2-As(2,4,6-Me-C_6H_2)}$_2$Al(C_5Me_5)] (**4**). The J. Young NMR tube was then placed in the glove box, filtered, and the filtrate collected into a scintillation vial. All volatiles were removed under reduced pressure, and the resulting greenish-brown solid was extracted with diethyl ether (2 × 3 mL) to yield 49 mg (0.046 mmol, 83%) of a dark green powder.

^{1}H NMR (C_6D_6, 600 MHz, 298 K): δ 10.40 (bs, 12H, o-C*H*$_3$), 9.43 (bs, 30H, UC_5*Me*$_5$), 5.99 (bs, 4H, m-*H*), 3.72 (s, 6H, p-C*H*$_3$), -6.71 (s, 15H, AlC_5*Me*$_5$).

IR (KBr, cm^{-1}): 2958 (s), 2909 (s), 2857 (s), 2722 (w), 2369 (w), 2308 (w), 2090 (w), 1627 (w), 1599 (w), 1447 (m), 1377 (m), 1261 (m), 1085 (m), 1018 (s), 846 (w), 803 (w), 803 (w), 617 (w).

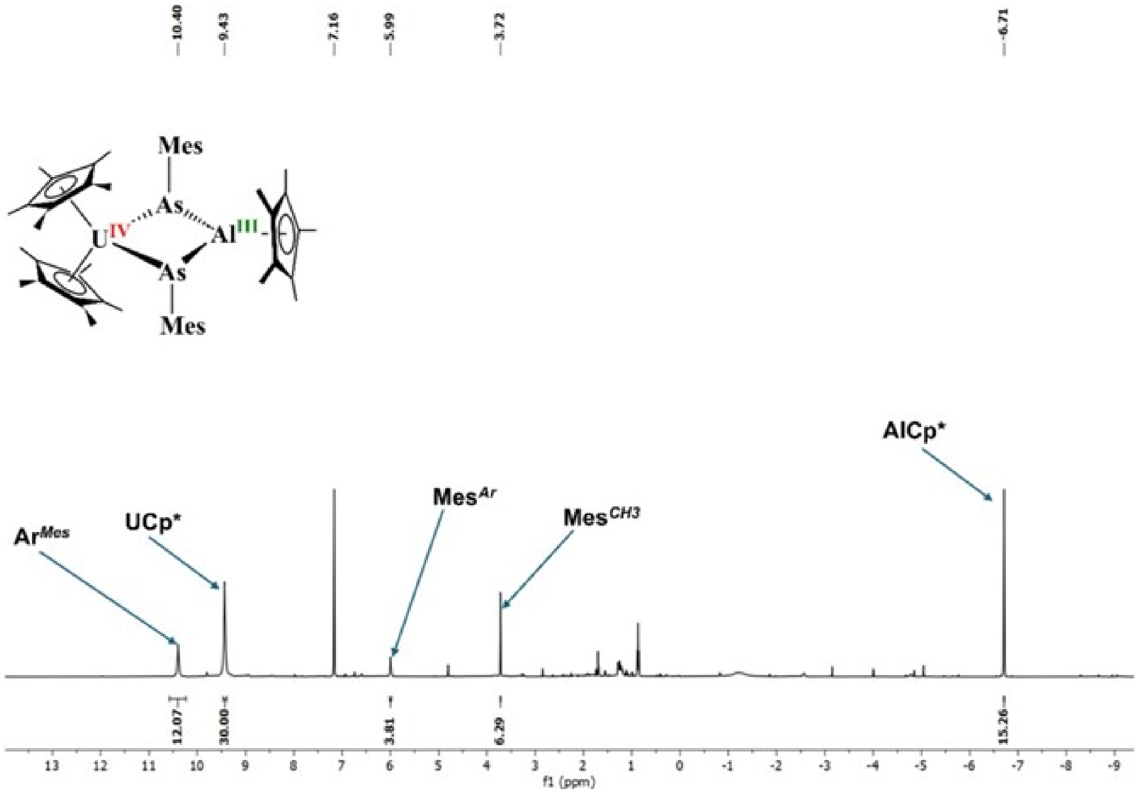

Figure S10. ^{1}H NMR (600 MHz, C_6D_6) of compound **4**.

Synthesis of [$(C_5Me_5)_2Th(CH_3)(\eta^2\text{-}N(H)(C_6H_5)N(C_6H_5)$] (5a).

To a J. Young NMR tube charged with 50 mg (0.093 mmol) of [$(C_5Me_5)_2Th(CH_3)_2$], 0.6 mL benzene-d_6 solution of PhN(H)N(H)Ph (17.3 mg, 0.093 mmol) was added at room temperature to witness the instant evolution of CH_4 gas. The NMR tube was sealed and allowed to stand at room temperature for 1 hour to observe a light, green-coloured solution. The NMR spectrum recorded after 1 h showed the complete consumption of [$(C_5Me_5)_2Th(CH_3)_2$], with the formation of **5a**. After 1 h, the J. Young NMR tube was brought back into a glovebox, and the filtrate was collected in a scintillation vial. All the volatiles were removed under reduced pressure to obtain a green colour crude solid. The crude product was extracted in 10 mL of Et_2O twice, filtered through diatomaceous earth, and concentrated to a green solid. Further purification of compound **5a** was achieved through crystallization using a solvent mixture of ether and pentane at -20 °C, yielding 55 mg (0.078 mmol, 85%) of green crystalline material.

^1H NMR (C_6D_6, 500 MHz, 298 K): δ 7.23 (t, $^3J_{H\text{-}H}$ = 7.83 Hz, 2H, *Ph*), 7.08 (t, $^3J_{H\text{-}H}$ = 7.63 Hz, 2H, *Ph*), 6.92 (d, $^3J_{H\text{-}H}$ = 8.07 Hz, 2H, *Ph*), 6.87 (t, $^3J_{H\text{-}H}$ = 7.05 Hz, 1H, *Ph*), 6.74 (d, $^3J_{H\text{-}H}$ = 7.41 Hz, 3H, *Ph*), 4.86 (s, 1H, N*H*), 1.99 (s, 15H, ThC_5Me_5), 1.86 (s, 15H, ThC_5Me_5), 0.08 (s, 1H, *Me*) ppm.

^{13}C NMR (C_6D_6, 125 MHz, 298 K): 152.8, 146.4, 129.6, 129.1, 123.9, 121.6 (*Ph*), 117.7, 117.6 (ThC_5Me_5), 115.3 (Ph), 58.8 (Me), 11.6, 11.3 (ThC_5Me_5) ppm.

IR (KBr, cm^{-1}): 3320 (s), 2953 (s), 2900 (s), 2855 (s), 2725 (w), 2090 (m), 1712 (w), 1600 (w), 1550 (w), 1456 (s), 1375 (m), 1261 (m), 1091 (m), 1022 (s), 845 (s), 802 (m), 705 (w), 684 (w), 611 (w), 543 (w).

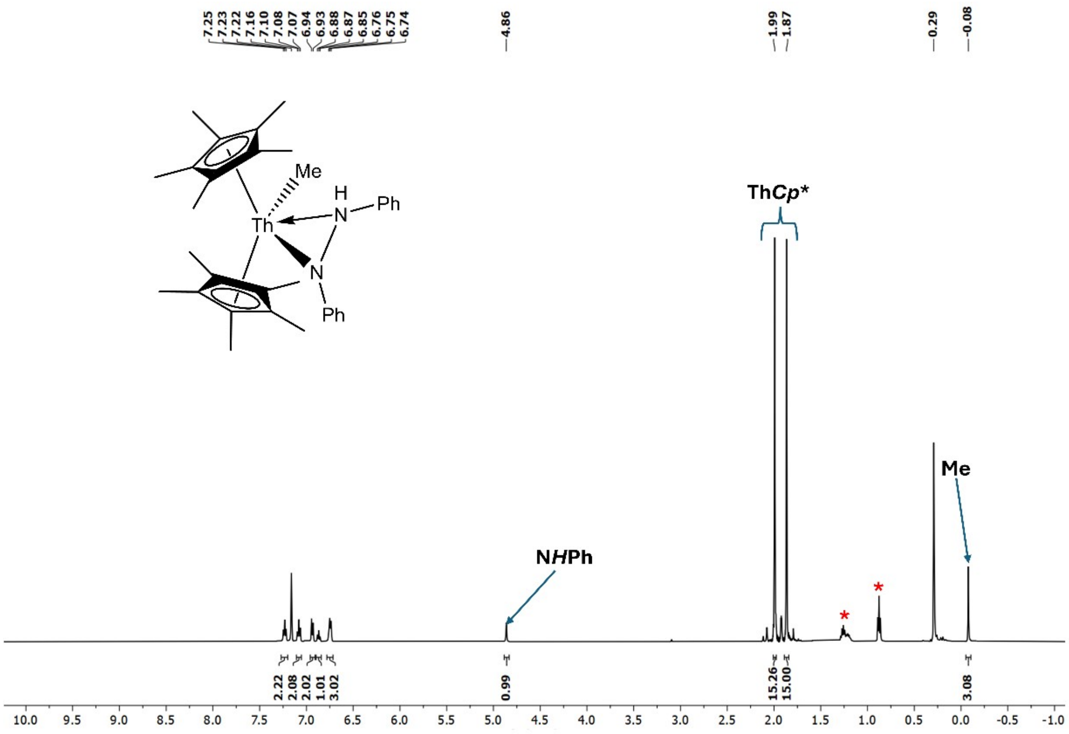

Figure S11. ^{1}H NMR (600 MHz, C_6D_6) of compound **5a**. *n-pentane

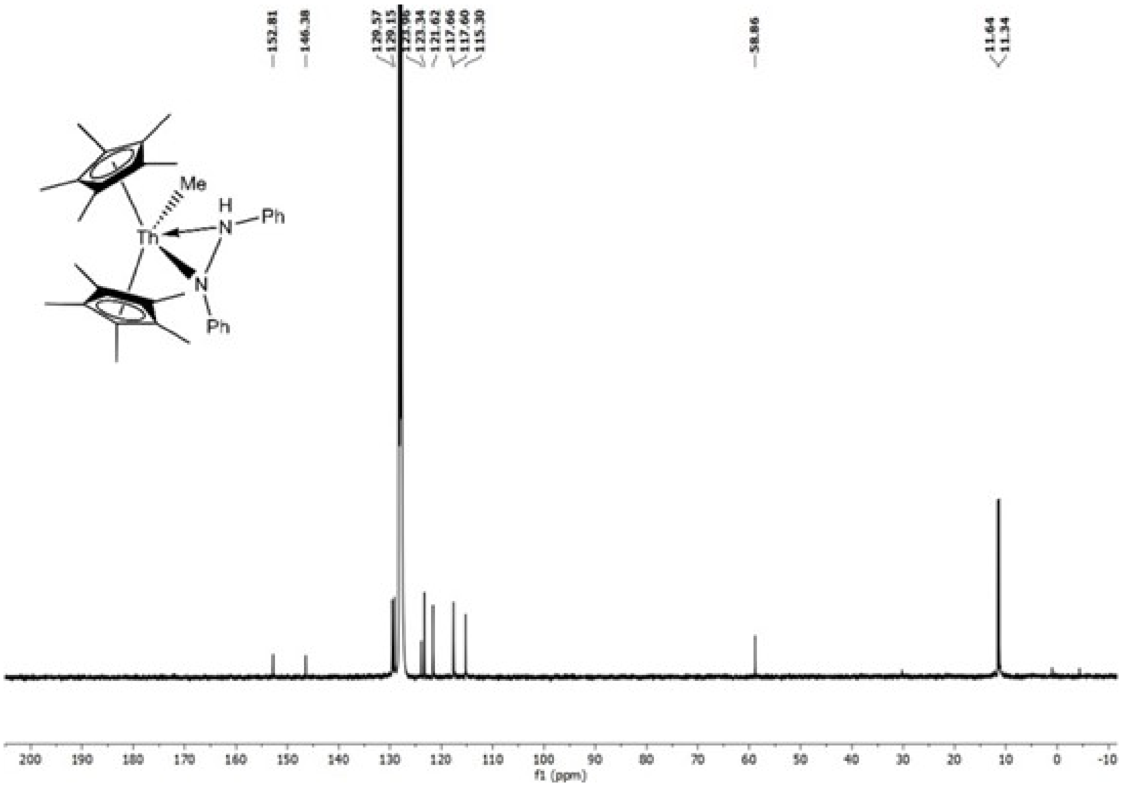

Figure S12. ^{13}C NMR (150 MHz, C_6D_6) of compound **5a**.

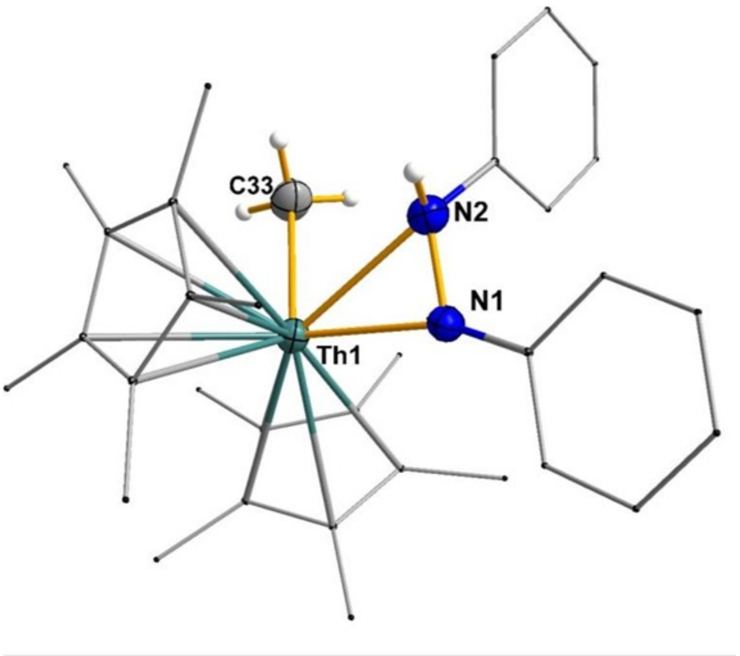

Figure S13. Molecular structure of compound **5a**. All the hydrogen atoms are omitted for clarity. Displacement parameters are shown at 50% probability; Selected interatomic distances [Å] and angles [°]: Th1–C33 1 2.9815(10), Th1–N1 2.8462(11), Th11–N2 2.463(3), N2–N1 2.376(3); N1–Th1–N2 77.61(3), N1–Th1–C33 88.65(7), N2–Th1–C33 93.67(4).

Synthesis of [$(C_5Me_5)_2Th\{\eta^2$-$N(C_6H_5)\}_2$(thf)] (5b).

Method 1: To a J. Young NMR tube charged with 50 mg (0.071 mmol) of **5a**, ca. 0.6 mL benzene-d_6 was added at room temperature, and the sealed NMR tube was heated to 70°C for 3 h to observe a solution colour change from bright green to light purple. After 3 h, the J. Young NMR tube was brought back into a glovebox, and the filtrate was collected in a scintillation vial. All the volatiles were removed under reduced pressure to obtain a light purple-colored crude solid. The crude product was extracted in 10 mL of Et_2O twice, filtered through diatomaceous earth, and concentrated to a light purple solid. Further purification of compound **5b** was achieved through crystallization using a solvent mixture of ether and pentane at -20 °C, yielding 42 mg (0.061 mmol, 88%) of purple crystalline material.

Method 2: To a J. Young NMR tube charged with 50 mg (0.093 mmol) of [$(C_5Me_5)_2Th(CH_3)_2$], 0.6 mL benzene-d_6 solution of PhN(H)N(H)Ph (17.3 mg, 0.093 mmol) was added at room temperature to witness the instant evolution of CH_4 gas. The NMR tube was sealed and heated to 70°C for 3h to observe a solution colour change from colourless to light green and light purple. After 3 h, the J. Young NMR tube was brought back into a glovebox, and the filtrate was collected in a scintillation vial. All the volatiles were removed under reduced pressure to obtain a light purple-colored crude solid. The crude product was extracted in 10 mL of Et_2O twice, filtered through diatomaceous earth, and concentrated to a light purple solid. Further purification of compound **5b** was achieved through crystallization using a solvent mixture of ether and pentane at -20 °C, yielding 50 mg (0.073 mmol, 78%) of purple crystalline material.

^{1}H NMR (C_6D_6, 400 MHz, 298 K): δ 7.30 (t, 4H, *Ph*), 6.77 (t, 4H, *Ph*), 1.77 (s, 30H, ThC_5*Me*$_5$) ppm.

^{13}C NMR (C_6D_6, 101 MHz, 298 K): 154.6, 126.7, 124.1 (*Ph*), 116.8 (Th*C*$_5$Me$_5$), 115.9 (Ph), 10.8 (ThC_5*Me*$_5$) ppm.

IR (KBr, cm^{-1}): 2935 (m), 2896 (s), 2853 (m), 2723 (s), 2093 (m), 1599(w), 1549 (w), 1456 (s), 1375 (s), 1261 (w), 1173 (w), 1046 (w), 1021 (s), 845 (s), 804 (w), 704 (w), 602 (w), 543 (w).

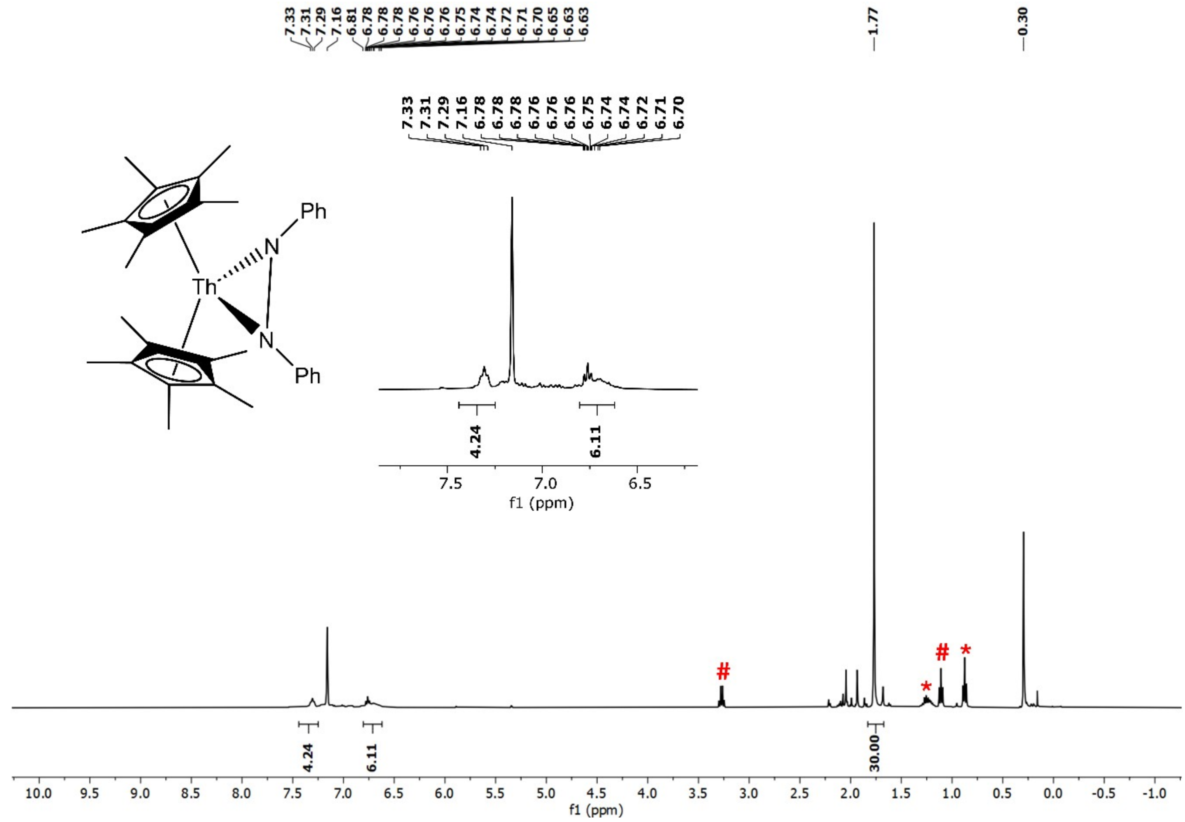

Figure S14. ^{1}H NMR (400 MHz, C_6D_6) of compound **5b**. *n-pentane, #diethylether

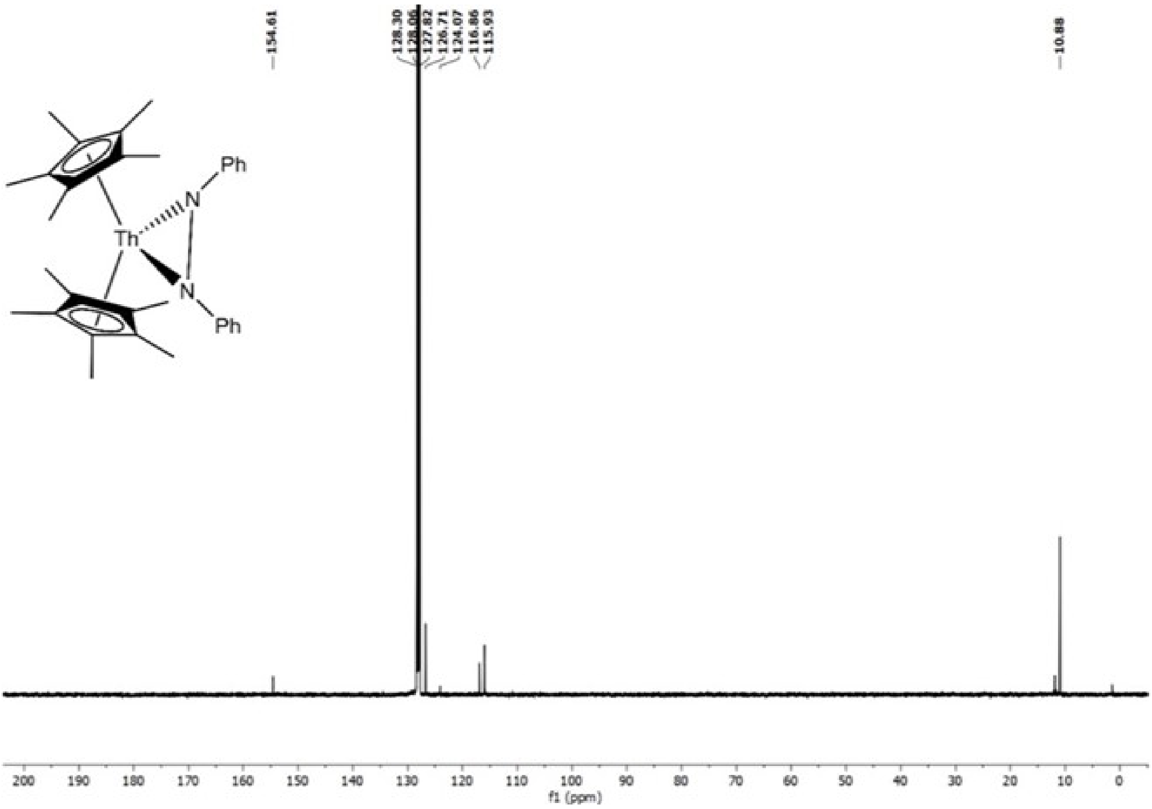

Figure S15. ^{13}C NMR (101 MHz, C_6D_6) of compound **5b**.

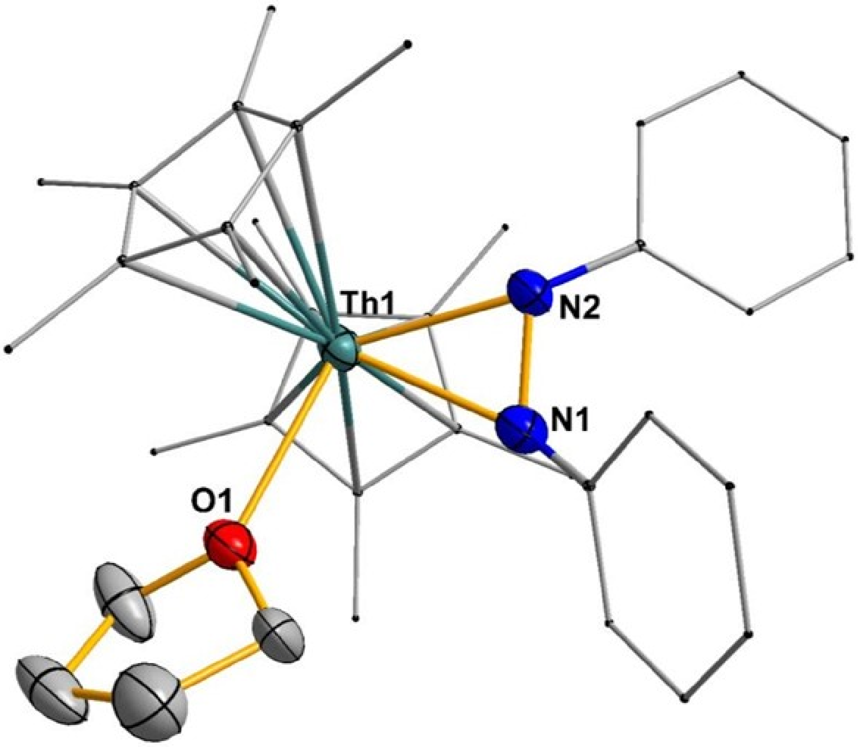

Figure S16. Molecular structure of compound **5b**. All the hydrogen atoms are omitted for clarity. Displacement parameters are shown at 50% probability; Selected interatomic distances [Å] and angles [°]: Th1–O1: 2.543(3), Th1–N1: 2.338(3), Th1–N2: 2.272(3), N2–N1: 1.469(4); N1–Th1–N2:37.12(11), N1–Th1–O1: 86.48(10), N2–Th1–O1: 120.81(11).

Synthesis of [$(C_5Me_5)_2Th\{\mu^2\text{-}N(C_6H_5)\}_2Al(C_5Me_5)$] (5).

A J. Young NMR tube was charged with 50 mg (0.073 mmol) of $(C_5Me_5)_2Th\{\eta^2\text{-}[N(C_6H_5)]_2\}$ (**5b**), 13.00 mg (0.020 mmol) $[(C_5Me_5)Al]_4$, and ca. 0.6 mL of benzene-d_6. The sealed NMR tube was shaken vigorously and heated for 15 minutes at 70 °C to observe a solution colour change from purplish green to yellowish orange. The NMR recorded after 15 minutes showed the complete consumption of $[(C_5Me_5)_2Th\{\eta^2\text{-}N(C_6H_5)\}_2]$ with the formation of compound $[(C_5Me_5)_2Th\{\mu_2\text{-}[N(C_6H_5)\}_2Al(C_5Me_5)]$ (**5**). The J. Young NMR tube was then taken inside the glove box, filtered, and the filtrate was collected in a scintillation vial. All volatiles were removed under reduced pressure, and the resulting crude orange powder was extracted with diethyl ether (2 × 3 mL) to obtain a yellowish orange-colored powder. Further purification of compound **5** was achieved through crystallization using a solvent mixture of ether and pentane at -20 °C, yielding 51 mg (0.060 mmol, 83%) of yellowish orange crystalline material.

^{1}H NMR (C_6D_6, 400 MHz, 298 K): δ 7.32 (t, $^{3}J_{H\text{-}H}$ = 7.80 Hz, 4H, *Ph*), 6.86 (t, $^{3}J_{H\text{-}H}$ = 6.84 Hz, 2H, *Ph*), 6.76 (d, $^{3}J_{H\text{-}H}$ = 7.57 Hz, 4H, *Ph*), 1.95 (s, 30H, ThC$_5$*Me*$_5$), 1.92 (s, 15H, AlC$_5$*Me*$_5$).

^{13}C NMR (C_6D_6, 101 MHz, 298 K): 11.6, 11.9, 115.0, 117.6, 123.1, 124.8, 125.1, 129.6, 153.3.

IR (KBr, cm^{-1}): 2971 (m), 2914 (m), 2857 (w), 1599 (w), 1508 (s), 1381 (m), 1229 (m), 1117 (m), 954 (m), 832 (m), 800 (w), 704 (w), 610 (w), 541 (w).

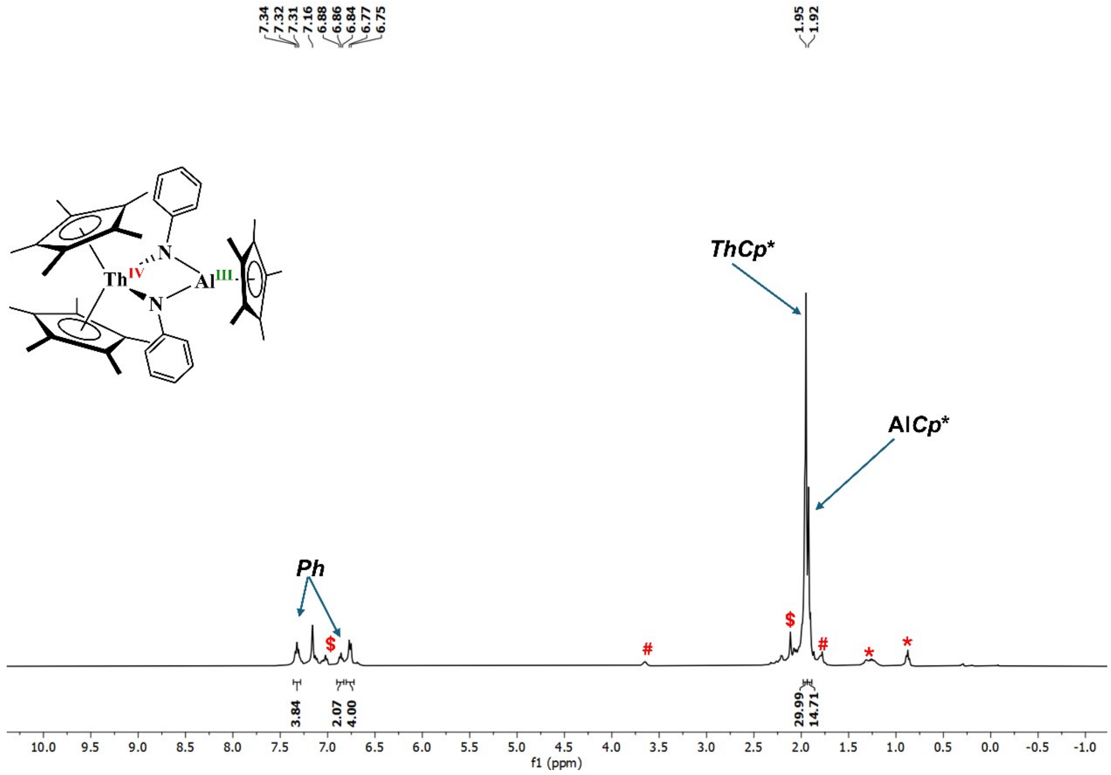

Figure S17. ^{1}H NMR (400 MHz, C_6D_6) of compound **5**. *n-pentane; $toluene; #THF

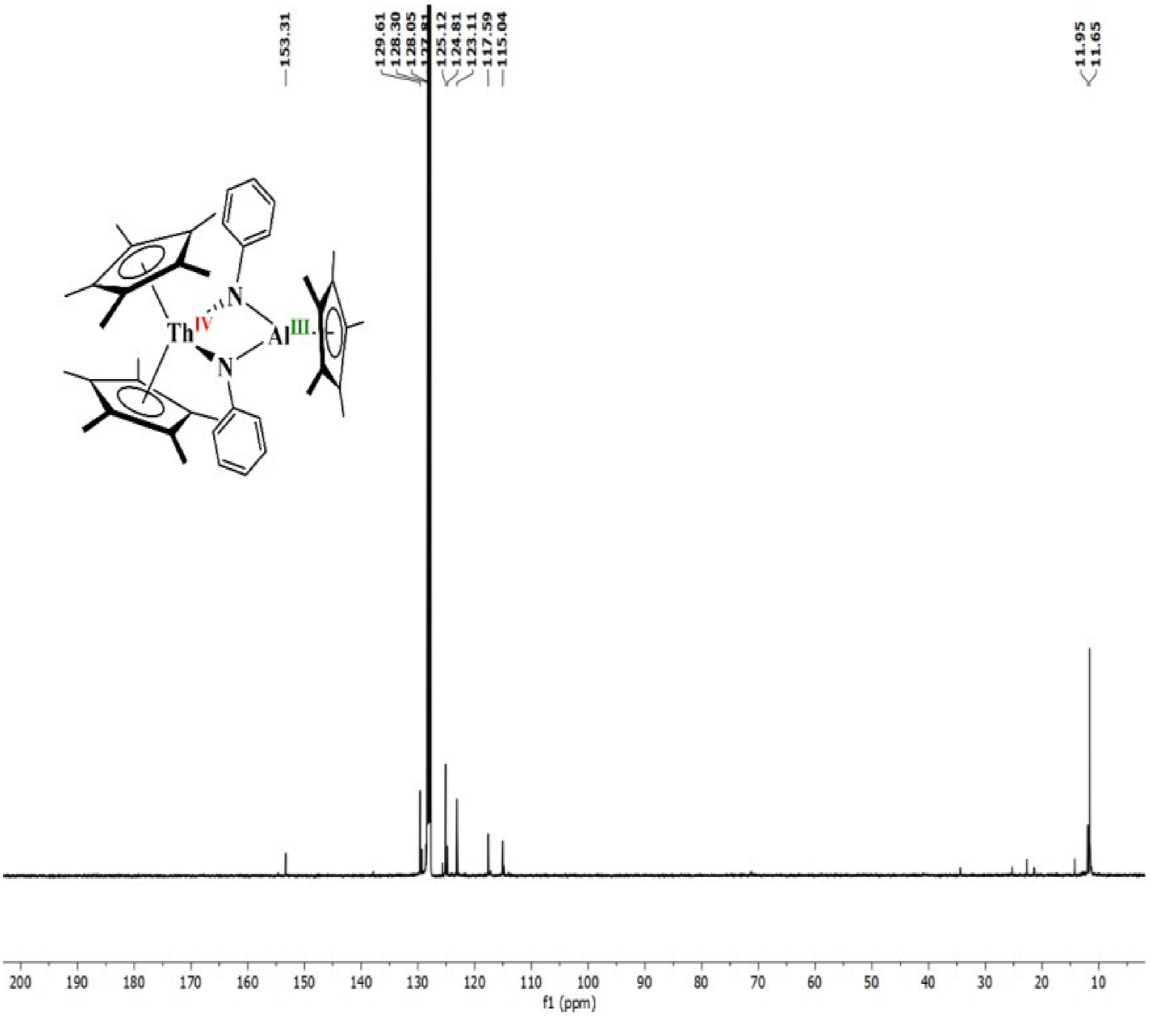

Figure S18. ^{13}C NMR (101 MHz, C_6D_6) of compound **5**.

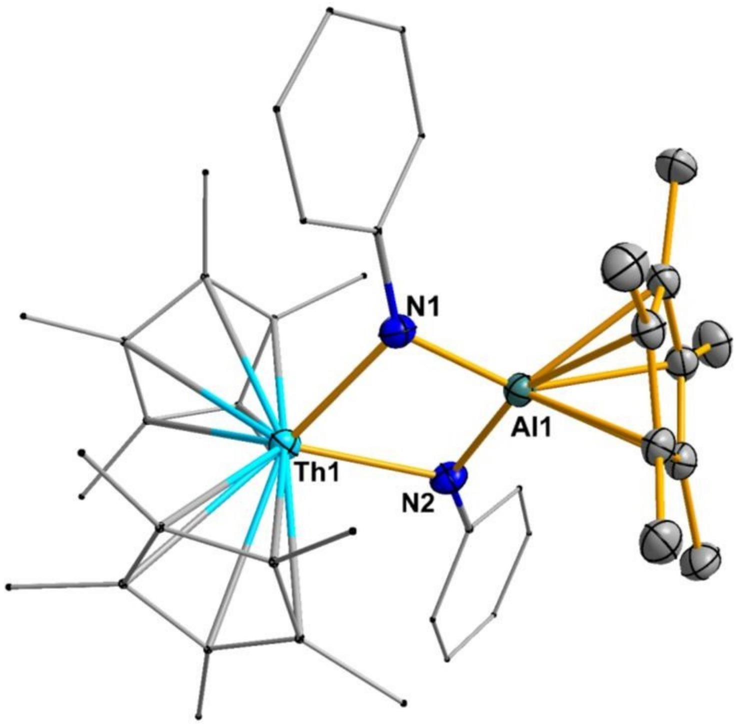

Figure S19. Molecular structure of compound **5**. All the hydrogen atoms are omitted for clarity. Displacement parameters are shown at 50% probability; Selected interatomic distances [Å] and angles [°]: Th1–N1: 2.281(2), Th1–N2: 2.302(2), N1–Al1: 1.821(2), N2–Al1: 1.816(2); N1–Th1–N2: 69.72(8), Th1–N1–Al1: 88.65(7), Th1-N2-Al1: 98.45(9), N1-Al1-N2: 92.14(10).

Synthesis of $[(C_5Me_5)_2U\{\mu_2\text{-}N(C_6H_5)\}_2Al(C_5Me_5)]$ (6).

A J. Young NMR tube was charged with 50 mg (0.080 mmol) of $[(C_5Me_5)_2U(=N(C_6H_5)]_2$, 14.3 mg (0.022 mmol) $[(C_5Me_5)Al]_4$, and ca. 0.5 mL of benzene-d_6. The sealed NMR tube was shaken vigorously and heated for 10 minutes at 70 °C to observe a colour change from dark brownish black to red. The ^{1}H NMR recorded after 10 minutes showed the complete consumption of $[(C_5Me_5)_2U(=N(C_6H_5))]_2$ with the formation of compound $[(C_5Me_5)_2U\{\mu_2\text{-}[N(C_6H_5)\}_2Al(C_5Me_5)]$ (**6**). The J. Young NMR tube was then taken inside the glove box, filtered, and the filtrate was collected in a scintillation vial. All the volatiles were removed under reduced pressure, and the resulting crude red powder was extracted with diethyl ether (2 x 3 mL) to obtain an orangish-red powder. Further purification of compound **6** was achieved through crystallization using a solvent mixture of ether and pentane at -20 °C, yielding 55 mg (0.064 mmol, 80%) of bright orangish-red crystalline material.

^{1}H NMR (C_6D_6, 600 MHz, 298 K): δ 4.42 (s, 30H, UC_5*Me*$_5$), 0.79 (s, 15H, AlC_5*Me*$_5$), -2.25 (bs, 4H, *Ph*), -3.78 (bs, 4H, *Ph*), -25.34 (bs, 2H, *Ph*).

IR (KBr, cm^{-1}): 2962 (s), 2907 (s), 2858 (s), 2723 (w), 2279 (w), 2903 (w), 1627 (w), 1599 (w), 1439 (m), 1384 (m), 1261 (m), 1092 (m), 1020 (s), 846 (m), 617 (m), 519 (m).

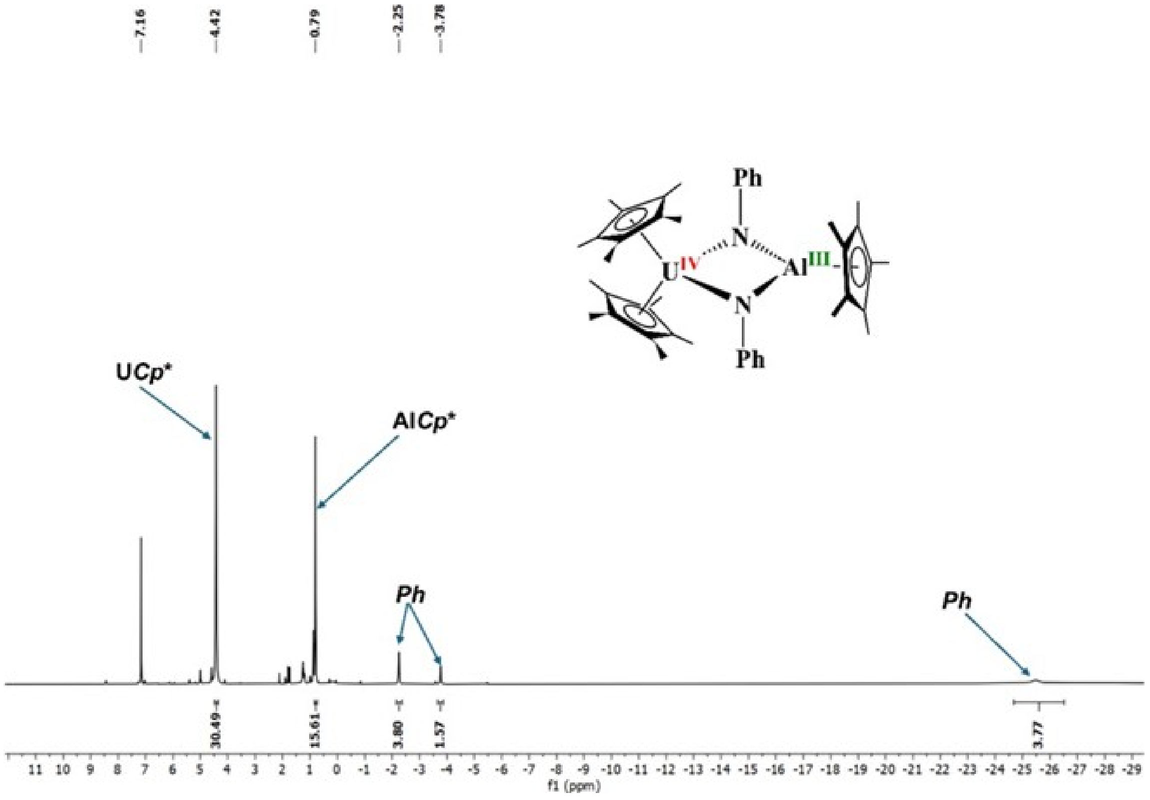

Figure S20. ^{1}H NMR (600 MHz, C_6D_6) of compound **6**.

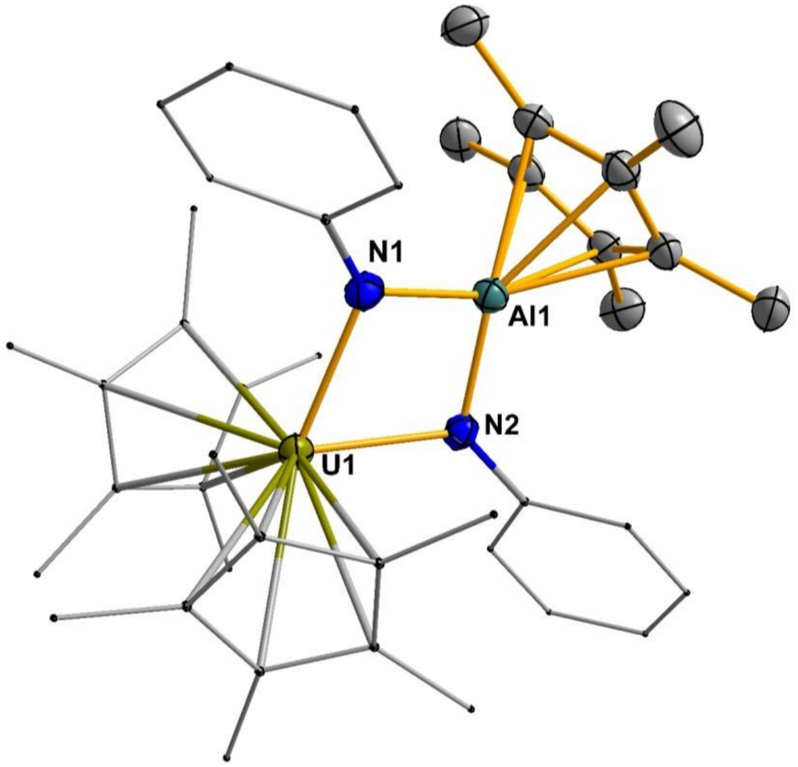

Figure S21. Molecular structure of compound **6**. All the hydrogen atoms are omitted for clarity. Displacement parameters are shown at 50% probability; Selected interatomic distances [Å] and angles [°]: U1–N1: 2.244(3), U1–N2: 2.225(2), N1–Al1: 1.836(3), N2–Al1: 1.833(3), N1–Al1: 3.632(9); N1–U1–N2: 71.75(9), U1–N1–Al1: 97.80(11), U1–N2–Al1: 98.57(11), N1-Al1-N2: 91.07(12).

2. Crystallographic Data Collection and Structure Determination.

SCXRD data for **1**, **6**, **5a**, and **5b** were collected on a Bruker D8 Venture diffractometer equipped with a Photon II CMOS area detector using Mo-Kα radiation from a microfocus source (Bruker AXS, Madison, WI, USA). SCXRD data for **2**, **3**, **A**, and **5** were collected on a Bruker SMART diffractometer equipped with an Apex II CCD area detector using Mo-Kα radiation from a sealed source tube with a focusing collimator (Bruker AXS). Crystals were cooled to their collection temperatures under streams of cold N_2 gas using Cryostream 700 or 800 cryostats (Oxford Cryosystems, Long Hanborough, UK). The selected single crystal of each complex was coated with viscous hydrocarbon oil inside a glove box before being mounted on a Kapton cryoloop using Parabar® hydrocarbon oil. Hemispheres of unique data were collected using strategies of scans about the omega and phi axes. Data collection, unit cell determination, data reduction, absorption correction, scaling, and space group determination were performed using the Apex3[8] or Apex4[9] software suites.

The structures of **1**, **3**, **A**, and **5b** were solved by an iterative dual space method as implemented in SHELXT.[S10] The structures of **2**, **6**, **5a**, and **5** were solved by direct methods as implemented in SHELXS v.2013.[S10] All structures were refined by full-matrix least squares against F^2 using SHELXL v.2019/3.[S10] Olex2[S11] was used as a graphical interface for model building and structure visualization. Non-hydrogen atoms were located from the difference map and refined anisotropically. Hydrogen atoms bonded to carbon were placed in calculated positions, and their coordinates and thermal parameters were restrained to ride on the carrier atoms. The amide hydrogen atom in **A** was refined by restraining a difference map peak in a chemically reasonable position to a distance of 0.90 Å from the N atom. This atom is disordered by symmetry across both sites and was fixed at an occupancy of 50%. For compound **2**, the crystal used was extremely small, and diffraction photographs showed numerous peaks consistent with scattering from satellite crystals. Extranneous scattering and very weak high-angle data result in anomalous difference map peaks and thermal parameters, and this structure was refined with restraints on the anisotropic displacement parameters to approximate rigid bond behavior.[S12] These artifacts can make element assignment ambiguous in some cases, but the accuracy of **2** is supported by its isomorphous relationship to the phosphorous analog. Compound **5a** had significant difference map peaks in chemically unrealistic positions, which appear to correspond to projections of heavy atoms translated in the *b** direction. These are attributed to packing faults, which can occur because these organometallic molecules are approximately globular, lack directional interactions, and are capable of orientational disorder. The refinement of **5b** converged with some carbon atoms having anomalously small and prolate thermal ellipsoids. This is attributed to systematic overestimation of F^2 due to diffraction from satellite crystals. The accuracy of element assignments for affected atoms can be inferred as they are part of known ligands that converge to expected geometries.

Refinement results are given in Tables S1 and S2. Graphical representations were generated using the DIAMOND program.[S13] CCDC-2501693 (**1**), CCDC-2501694 (**2**), CCDC-25016958(**3**), CCDC-2501696 (**A**), CCDC-2501697 (**6**), CCDC-2501698 (**5a**), CCDC-2501699 (**5b**), and CCDC-2501700 (**5**) contain the supplementary crystallographic data for this paper. These data can be obtained free of charge from the Crystallographic Data Centre via www.ccdc.cam.ac.uk/data_request/cif

Table S1. Crystallographic data of **1**, **2**, **3**, and **A.**

	$1 \cdot 0.5(C_4H_{10}O)$	$2 \cdot 0.5(C_5H_{12})$	$3 \cdot 0.5(C_5H_{12})$	**A**
formula	$C_{50}H_{72}AlP_2Th$	$C_{50.5}H_{67}AlAs_2Th$	$C_{50.5}H_{73}AlP_2U$	$C_{38}H_{51}AlN_2U$
Fw / g·mol^{-1}	1002.03	1088.94	1007.03	800.81
Temperature/K	173.0	150.0	150.0	150.0
cryst. color, habit	orange, irregular block	Red, irregular block	red, block	brown, block
crystal size / mm	0.10 × 0.08 × 0.03	0.14 × 0.05 × 0.04	0.12 × 0.12 × 0.02	0.18 × 0.14 × 0.08
crystal system	triclinic	triclinic	triclinic	orthorhombic
space group	*P*-1	*P*-1	*P*-1	*Pbcn*
a/Å	10.7096(6)	10.7863(16)	10.5718(8)	13.062(3)
b/Å	15.0349(10)	15.317(2)	14.7969(11)	13.198(3)
c/Å	15.6459(9)	15.579(2)	15.6151(12)	20.188(5)
α/°	91.198(2)	89.331(3)	91.625(2)	90
β/°	105.249(2)	73.603(3)	105.284(2)	90
γ/°	104.290(2)	74.673(3)	103.762(2)	90
Volume/Å^3	2345.4(2)	2375.9(6)	2277.8(3)	3480.4(15)
Z	2	2	2	4
ρ_{calc}g/cm^3	1.419	1.522	1.468	1.528
μ/mm^{-1}	3.299	4.570	3.686	4.716
F(000)	1018	1090.0	1022.0	1592
Radiation	MoKα (λ = 0.71073)	MoKα (λ = 0.71073)	MoKα (λ = 0.71073)	MoKα (λ = 0.71073)
θ range for data collection/°	2.756 to 27.505	1.366 to 26.643	1.358 to 27.499	2.018 to 29.066
Index ranges	$-13 \leq h \leq 13$, $-19 \leq k \leq 19$, $-20 \leq l \leq 20$	$-13 \leq h \leq 13$, $-19 \leq k \leq 19$, $-19 \leq l \leq 19$	$-13 \leq h \leq 13$, $-19 \leq k \leq 19$, $-20 \leq l \leq 20$	$-17 \leq h \leq 17$, $-17 \leq k \leq 18$, $-27 \leq l \leq 27$
Reflections collected	78332	33447	84525	68318
Independ. reflns (R_{int}, R_σ)	10715 [R_{int} = 0.0359 R_{sigma} = 0.0250]	9967 [R_{int} = 0.1044, R_{sigma} = 0.1232]	10463 [R_{int} = 0.0389, R_{sigma} = 0.0224]	4630 [R_{int} = 0.0557, R_{sigma} = 0.0229]
data/ restr./ param.	10715/0/540	9967/675/534	10463/0/537	4630/1/201

R_1, wR_2 [I> 2σ(I)]	0.0266, 0.0656	0.0599, 0.1316	R_1 = 0.0272, wR_2 = 0.0693	R_1 = 0.0377, wR_2 = 0.0698
R_1, wR_2 (all data)	0.0308, 0.0672	0.1085, 0.1488	R_1 = 0.0344, wR_2 = 0.0735	R_1 = 0.0625, wR_2 = 0.0771
GooF on F^2	1.045	0.997	1.132	1.233
largest diff. peak, hole / e·Å^{-3}	1.903, -0.755	4.821, -2.402	2.210, -0.703	1.433, -2.286
CCDC Number	2501693	2501694	2501695	2501696

Table S2. Crystallographic data of **6**, **5a-b**, and **5**

	**6**	**5a**	**5b·0.5($C_4H_{10}O$)**	**5**
formula	$C_{42}H_{55}N_2AlU$	$C_{33}H_{44}N_2Th$	$C_{36}H_{53}N_2O_{1.5}Th$	$C_{42}H_{55}AlN_2Th$
Fw / g·mol^{-1}	852.89	700.74	793.86	846.90
Temperature/K	173.0	173.0	173.0	150.0
cryst. color, habit	red, plate	yellow, plate	green, plate	Yellow, block
crystal size / mm	0.12 × 0.05 × 0.02	0.16 × 0.09 × 0.03	0.08 × 0.04 × 0.04	0.40 × 0.18 × 0.08
crystal system	monoclinic	triclinic	triclinic	monoclinic
space group	$P2_1/n$	*P*-1	*P*-1	$P2_1/n$
a/Å	10.5082(3)	9.5196(5)	10.7992(5)	10.5071(10)
b/Å	19.3141(6)	11.1473(6)	18.7301(9)	19.1915(19)
c/Å	18.1476(6)	15.1216(8)	19.8590(10)	18.1526(18)
α/°	90	82.637(2)	64.432(2	90
β/°	92.9368(12)	71.7644(19)	76.131(2)	92.7056(18)
γ/°	90	78.2949(19)	81.670(2)	90
Volume/Å^3	3678.3(2)	1488.78(14)	3513.9(3)	3656.3(6)
Z	4	2	4	4
ρ_{calc}g/cm^3	1.540	1.563	1.501	1.538
μ/mm^{-1}	4.468	5.030	4.275	4.134
F(000)	1704	692	1588	1696
Radiation	MoKα (λ = 0.71073)	MoKα (λ = 0.71073)	MoKα (λ = 0.71073)	MoKα (λ = 0.71073)
θ range for data collection/°	2.21 to 27.39	2.642 to 28.728	1.207 to 28.320	1.545 to 31.1951
Index ranges	-13 ≤ h ≤ 13, -25 ≤ k ≤ 25, -23 ≤ l ≤ 23	-12 ≤ h ≤ 12, -15 ≤ k ≤ 15, -20 ≤ l ≤ 20	-14 ≤ h ≤ 14, -24 ≤ k ≤ 24, -26 ≤ l ≤ 26	-15≤ h ≤ 15, -27 ≤ k ≤ 27, -26 ≤ l ≤ 26
Reflections collected	102546	49766	153634	138978
Independ. reflns (R_{int}, R_σ)	8449 [R_{int} = 0.0496R_{sigma} = 0.0250]	7714 [R_{int} = 0.0398, R_{sigma} = 0.0280]	17463 [R_{int} = 0.0487, R_{sigma} = 0.0303]	11805 [R_{int} = 0.0555, R_{sigma} = 0.0270]
data/ restr./ param.	8449/0/430	7714/0/339	17463/0/788	11805/0/430
R_1, wR_2 [I> 2σ(I)]	0.0237,0.0429	0.0246, 0.0481	R_1 = 0.0296, wR_2 =	R_1 = 0.0258, wR_2 = 0.0534

			0.0529	
R_1, wR_2 (all data)	0.0387, 0.0499	0.0309, 0.0517	R_1 = 0.0494, wR_2 = 0.0622	R_1 = 0.0400, wR_2 = 0.0576
GooF on F^2	1.084	1.089	1.077	1.089
largest diff. peak, hole / e·Å^{-3}	2.158, -0.939	2.675, -1.047	3.074, -1.351	1.338, -0.711
CCDC Number	2501697	2501698	2501699	2501700

3. UV-Visible spectrum of compounds 1,3, 5, and 6.

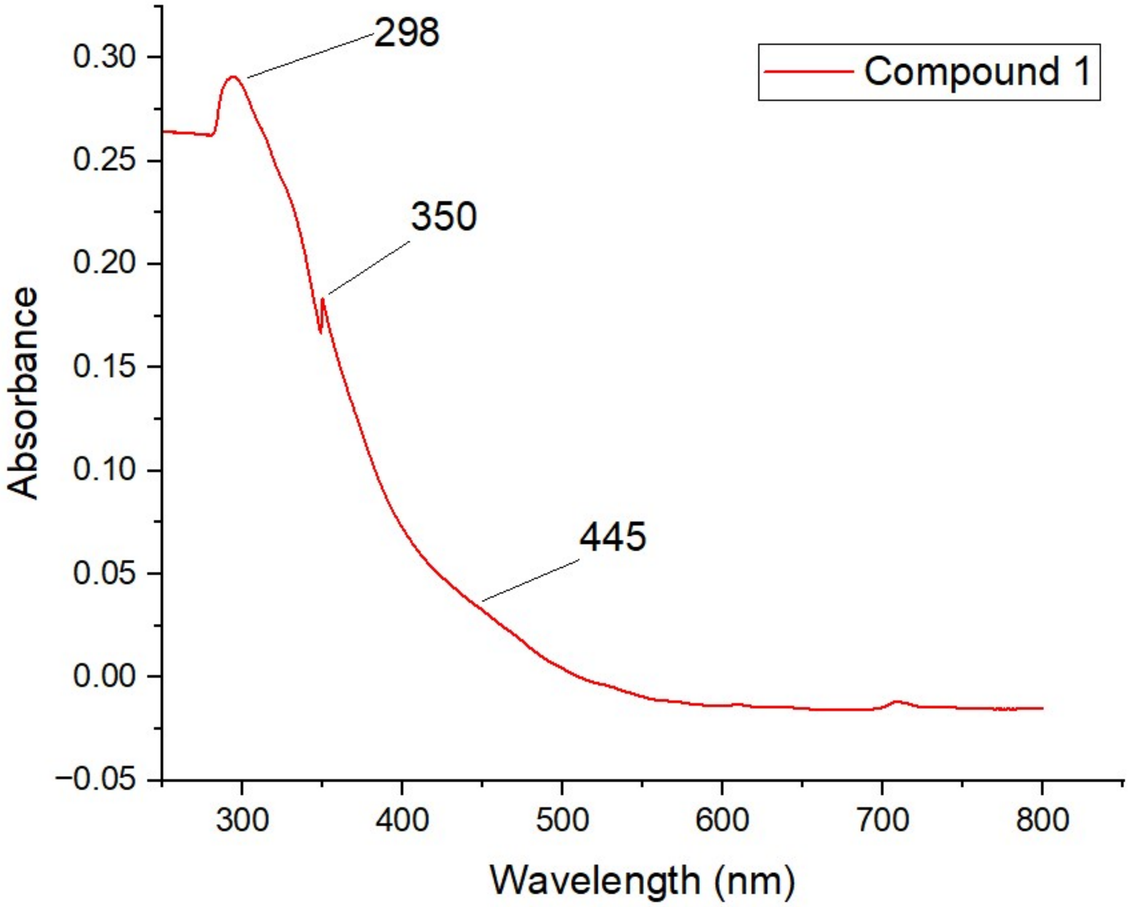

Figure S22. UV-visible spectrum of compound **1**

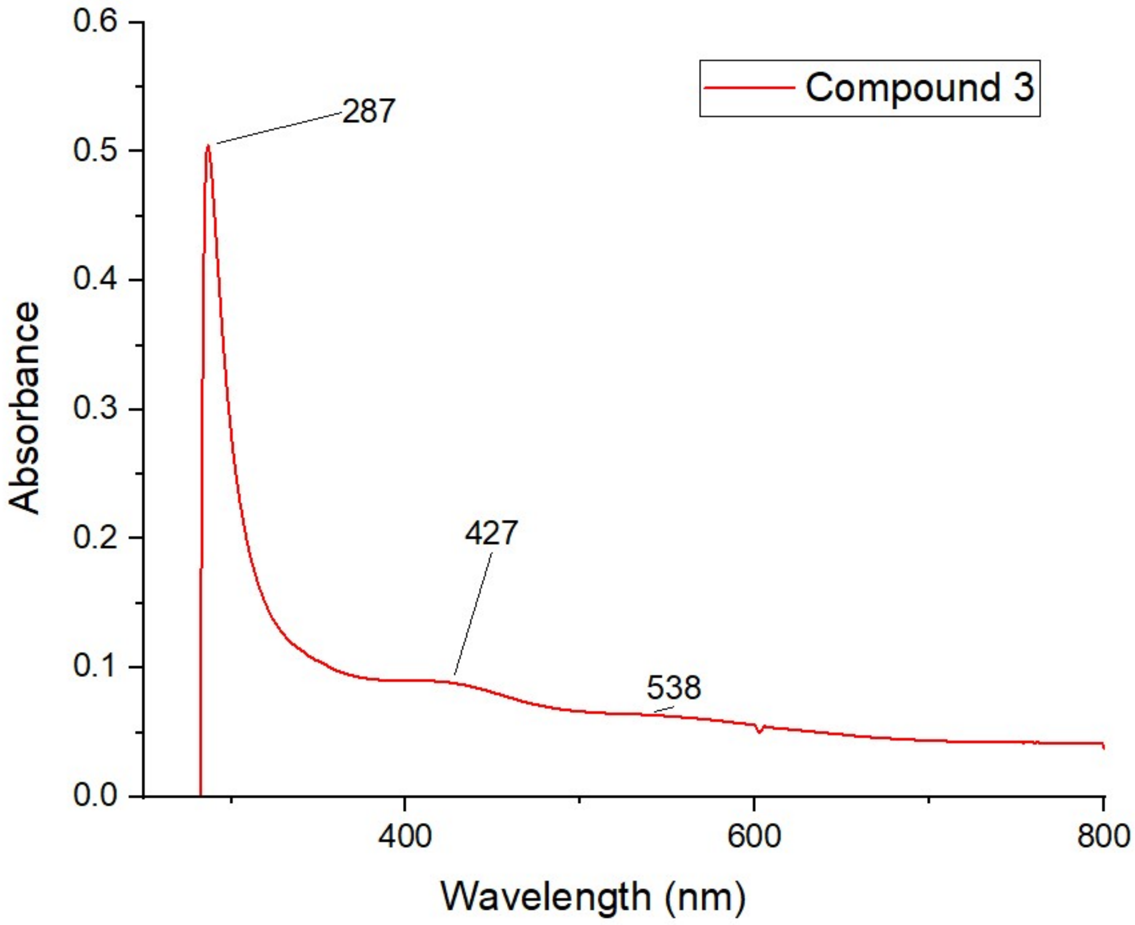

Figure S23. UV-visible spectrum of compound **3**

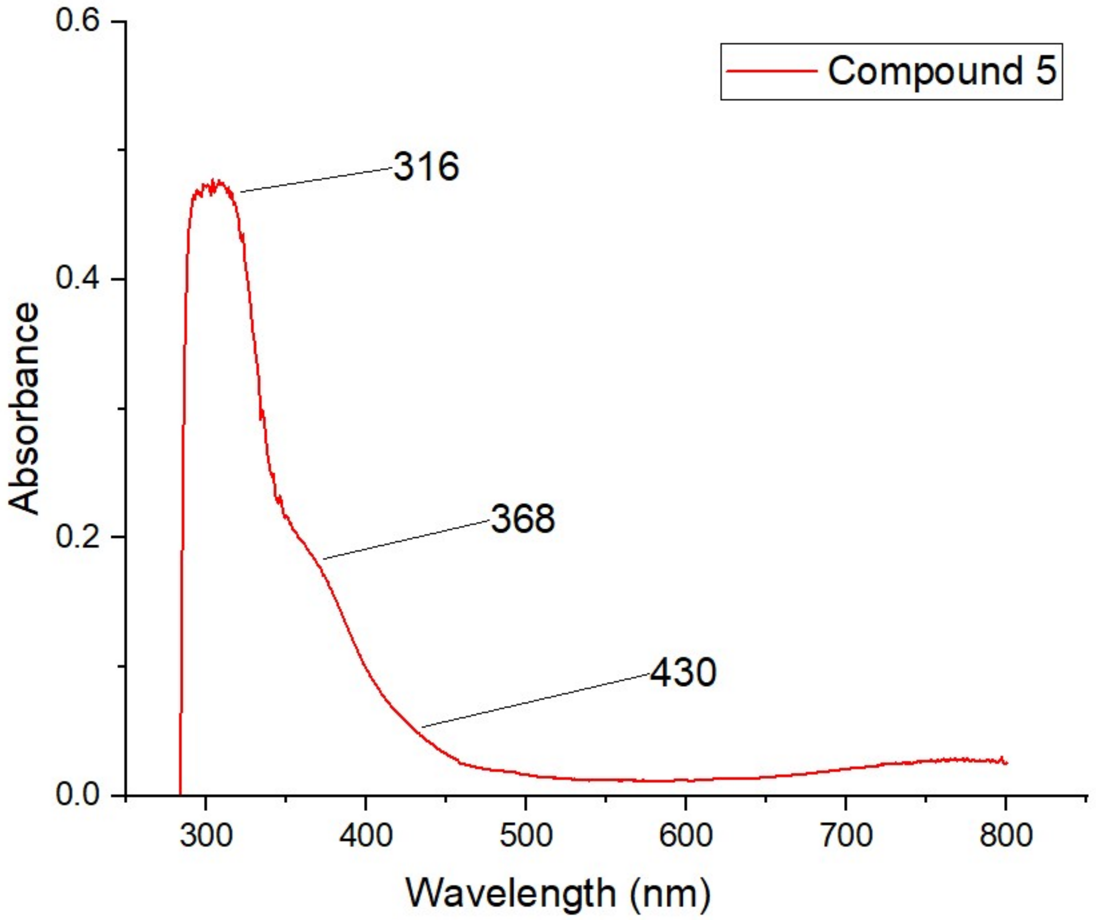

Figure S24. UV-visible spectrum of compound **5**

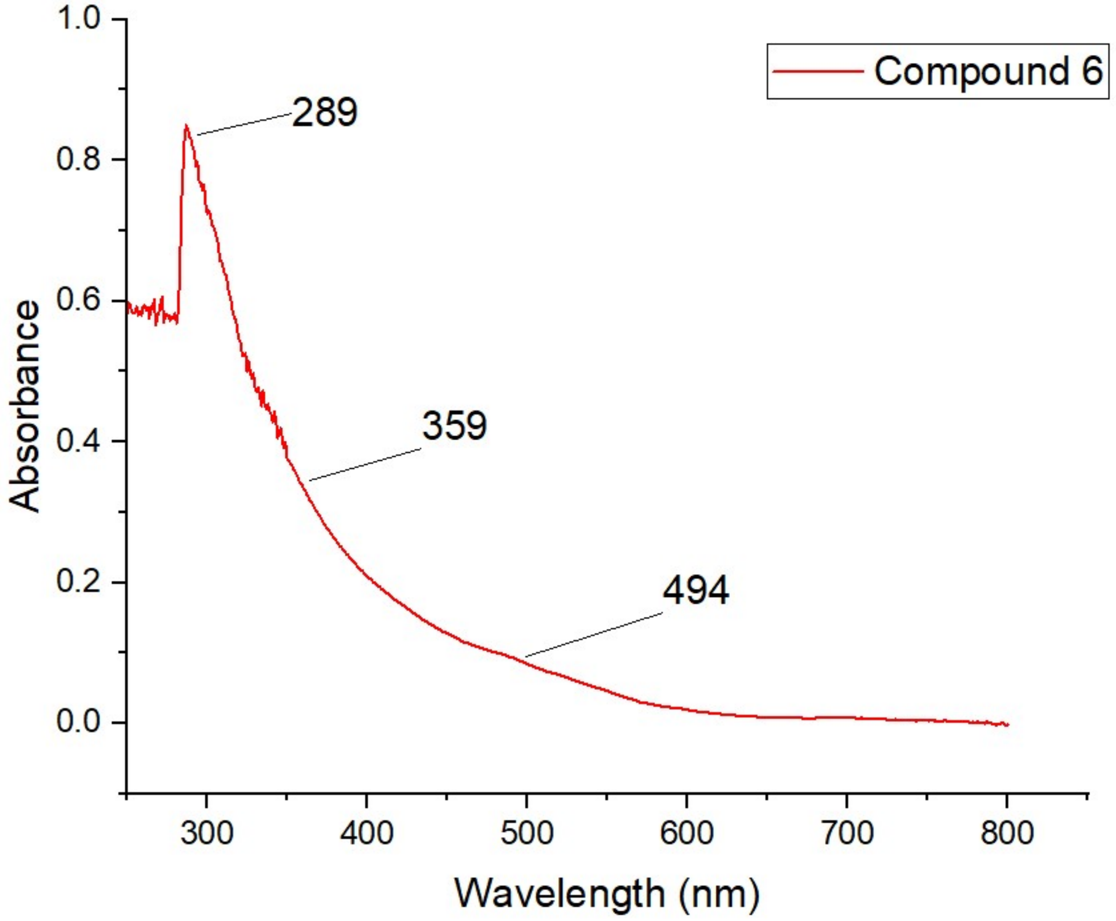

Figure S25. UV-visible spectrum of compound **6**

4. Computational details

All the DFT calculations were performed using the Gaussian09 suite of programs.[S14] Hybrid DFT functional (B3PW91) along with relativistic effective core potentials of the Stugart-Dresden_Köln type and their associated basis sets augmented by additional polarization functions for U & Al &As atoms and 6-31G** basis sets for the rest of the atoms were employed for the computation.[S15-16] Dispersion corrections were taken into account using the Grimme-3's scheme in conjunction with the Becke-Johnson (BJ) damping.

For Compound 1

Table S3. Comparison of selected bond distances between DFT optimized structures and X-ray for Th-P-Al

(S=3/2)	DFT		X-ray
	dispersion	No dispersion	
Th 1-P2	2.77182	2.84049	2.77090
Th 1-P3	2.88087	2.88374	2.89872
Al4-P2	2.30759	2.34136	2.21766
Al4-P3	2.38872	2.41433	2.37896
Th 1-X120	2.52225	2.56095	2.52573
Th 1-X121	2.54791	2.59754	2.57782

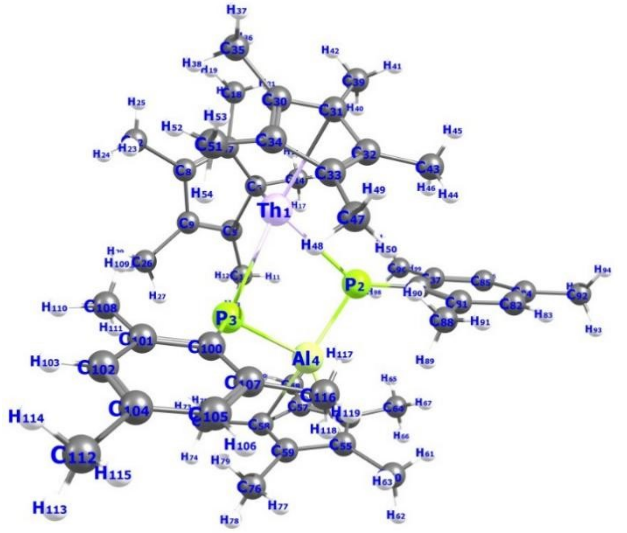

Table S4. Computed natural charges

Atom labels	Natural charges
Th1	0.82052
P2	-0.29682
P3	-0.29824
Al4	1.60983

Table S5. Computed Wiberg bond index between selected atoms

Atom labels	Wiberg bond index
Th1-P2	1.2589
Th1-P3	1.0688
Al4-P2	0.6927
Al4-P3	0.6347

Table S6. NBO bond analysis

Bond	% Contribution	Orbitals			
		s	p	d	f
(1.90734) BD (1)Th1-P2	0.4596*Th1 (21.12%)	18.49%	0.15%	65.03%	16.32%
	0.8881*P2 (78.88%)	46.96	52.99%	0.05%	-
(1.84399) BD (2)Th 1- P 2	0.4369*Th1 (19.09%)	0.25%	0.17%	57.17%	42.40%
	0.8995*P2 (80.91%)	2.45%	97.50%	0.05%	-
(1.84313) BD (1)Th 1- P 3	0.4866*Th1(23.68%)	20.10%	0.05%	67.38%	12.46%
	0.8736*P3 (76.32%)	26.05%	73.75%	0.20%	-
(1.81034) BD (1) P 2-Al 4	(80.29%) 0.8960* P2	27.87%	71.93%	0.20%	
	(19.71%) 0.4440*Al4	45.83%	52.97%	1.20	
(1.75196) BD (1) P 3-Al 4	(79.25%) 0.8902* P3	16.85%	82.80%	0.36	
	(20.75%) 0.4555*Al4	45.62	53.12%	1.26%	
(0.08313) BD*(1)Th 1- P 2	(78.88%) 0.8881*Th1	18.49%	0.15	65.03%	16.32%
	(21.12%) -0.4596* P2	46.96%	52.99%	0.05%	
(0.06448) BD*(2)Th 1- P 2	(80.91%) 0.8995*Th1	0.25%	0.17%	57.17%	42.40%
	(19.09%) -0.4369* P2	2.45%	97.50%	0.05%	
(0.09204) BD*(1)Th 1- P 3	(76.32%) 0.8736*Th1	20.10%	0.05%	67.38%	12.46%
	(23.68%) -0.4866* P3	26.05%	73.75%	0.20%	
(0.09575) BD*(1) P 2-Al 4	(19.71%) 0.4440* P2	27.87%	71.93	0.20%	
	(80.29%) -0.8960*Al4	45.83%	52.97%	1.20%	
(0.10269) BD*(1) P 3-Al 4	(20.75%) 0.4555* P3	16.85%	82.80	0.36%	
	(79.25%) -0.8902*Al4	45.62%	53.12%	1.26	

Table S7. NBO second order perturbation analysis

Donor NBO	Acceptor NBO	E(2) kcal/mol
(1.98361) CR(2)Th1<br>s(100.00%)	(0.24858) LV(1)Al4<br>s(1.85%)$p^{52.89}$(98.02%)$d^{0.07}$(0.12%)	13.42
(1.90734) BD(1)Th1- P2<br>Th1(sp$^{0.01}$d$^{3.52}$f$^{0.88}$)- P2(sp$^{1.13}$)	(0.24858) L(1)Al4<br>s(1.85%)$p^{52.89}$(98.02%)$d^{0.07}$(0.12%)	29.42
(1.90734) BD(1)Th1- P2<br>Th1(sp$^{0.01}$d$^{3.52}$f$^{0.88}$)- P2(sp$^{1.13}$)	(0.21622) L(2)Al4<br>s(5.89%)$p^{15.95}$(94.01%)$d^{0.02}$(0.10%)	17.08
(1.84399) BD(2)Th1- P2<br>Th1 (sp$^{0.70}$d$^{99.99}$f$^{99.99}$)-P2<br>sp$^{39.76}$d$^{0.02}$)	(0.21622) LV(2)Al4<br>s(5.89%)$p^{15.95}$(94.01%)$d^{0.02}$(0.10%)	18.52
(1.84313) BD(1)Th1- P3<br>Th1 (sd$^{3.35}$f$^{0.62}$)- P3 (sp$^{2.83}$)	(0.24858) LV(1)Al4<br>s(1.85%)$p^{52.89}$(98.02%)$d^{0.07}$(0.12%)	30.07
(1.84313) BD(1)Th1- P3<br>Th1 (sd$^{3.35}$f$^{0.62}$) - P3 (sp$^{2.83}$)	(0.21622) LV(2)Al4<br>s(5.89%)$p^{15.95}$(94.01%)$d^{0.02}$(0.10%)	24.10
(1.81034) BD(1)P2-Al4<br>P2 (sp$^{2.58}$d$^{0.01}$)- Al4 (sp$^{1.16}$d$^{0.03}$)	(0.37197) LV(1)Th1<br>s(0.07%)$p^{0.33}$(0.02%)$d^{99.99}$(81.76%)$f^{99.99}$(18.14%)	43.39
(1.81034) BD (1)P2-Al4<br>P2 (sp$^{2.58}$d$^{0.01}$)- Al4 (sp$^{1.16}$d$^{0.03}$)	(0.29042) LV(2)Th1<br>s(1.57%)$p^{0.01}$(0.02%)$d^{49.03}$(76.87%)$f^{13.73}$(21.53%)	16.77
(1.81034) BD(1)P2-Al4<br>P2 (sp$^{2.58}$d$^{0.01}$)- Al4 (sp$^{1.16}$d$^{0.03}$)	(0.21555) LV(4)Th1<br>s(34.67%)p0.00(0.02%)$d^{1.08}$(37.41%)$^{f0.80}$(27.90%)g 0.00(0.01%)	20.19

(1.81034) BD(1)P2-Al4<br>P2 ($sp^{2.58}d^{0.01}$)-Al4 ($sp^{1.16}d^{0.03}$)	(0.11809) LV(6)Th1<br>s(1.19%)$p^{0.23}$(0.27%)$d^{7.39}$(8.83%)$f^{75.08}$(89.68%)	35.29
(1.75196) BD(1)P3-Al4<br>P3 ($sp^{4.91}d^{0.02}$)-Al4 ($sp^{1.16}d^{0.03}$)	(0.37197) LV (1)Th1<br>s(0.07%)$p^{0.33}$(0.02%)$d^{99.99}$(81.76%)$f^{99.99}$(18.14%)	40.99
(1.75196) BD(1)P3-Al4<br>P3 ($sp^{4.91}d^{0}$·02)-Al4<br>($sp^{1.16}d^{0.03}$)	(0.21555) LV(4)Th1<br>s(34.67%)$d^{1.08}$(37.41%)$f^{0.80}$(27.90%)	22.14

Table S8. Computed Alpha Mos.

(199)HOMO (-0.15732 a.u.)	(198) (-0.16806 a.u.)
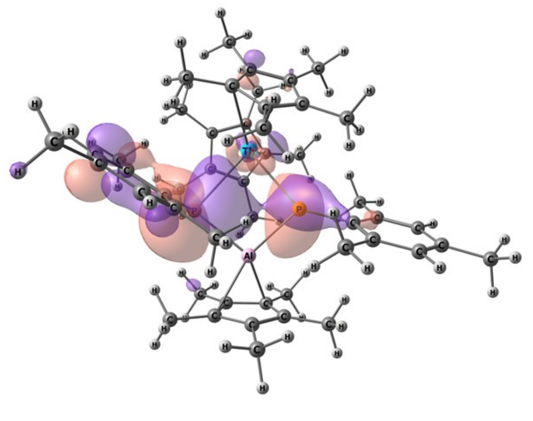	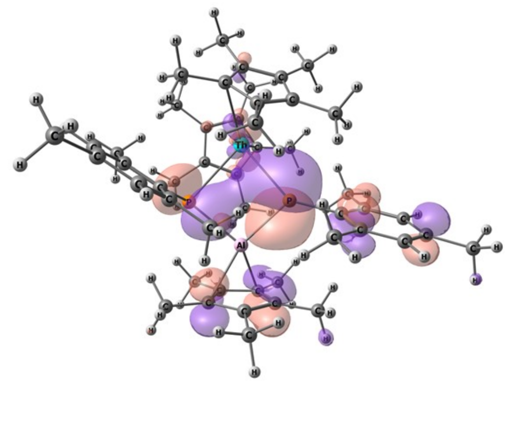
(197) (-0.17366 a.u.)	(194) (-0.21240 a.u.)
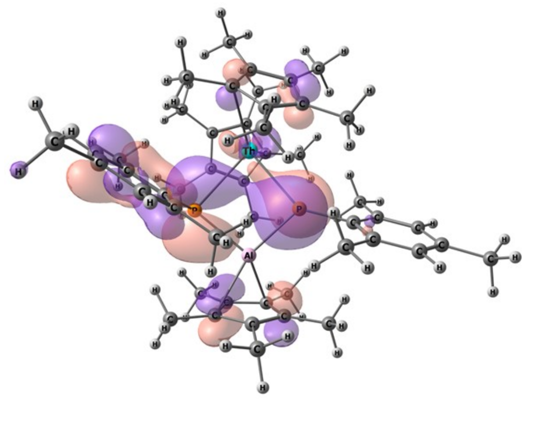	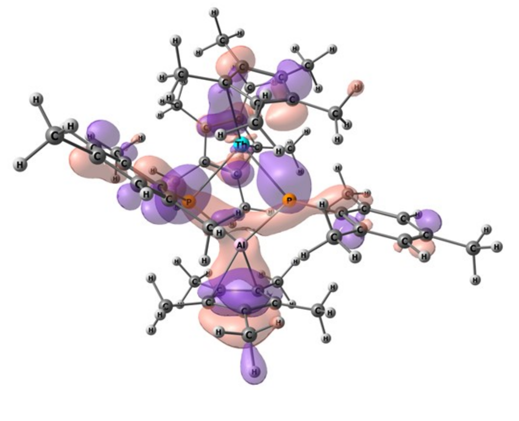
(187) (-0.24583 a.u.)	(186) (-0.25549 a.u.)

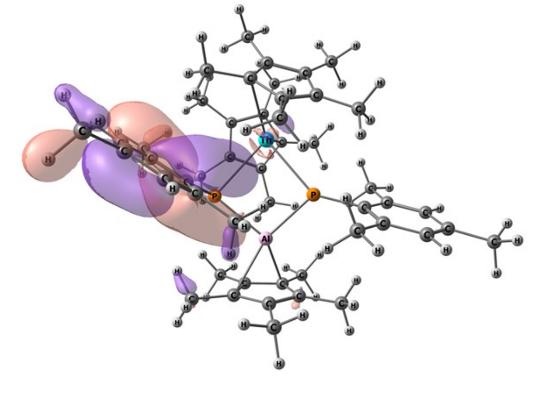

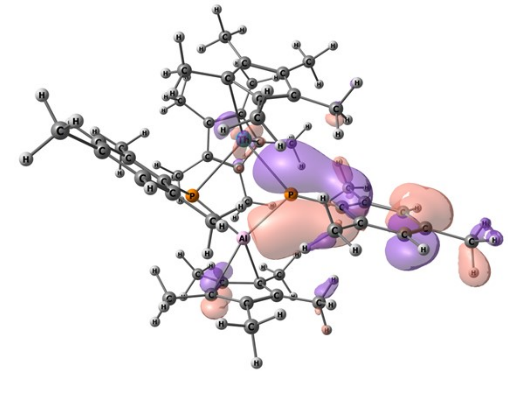

(103) (-0.52014 a.u.)

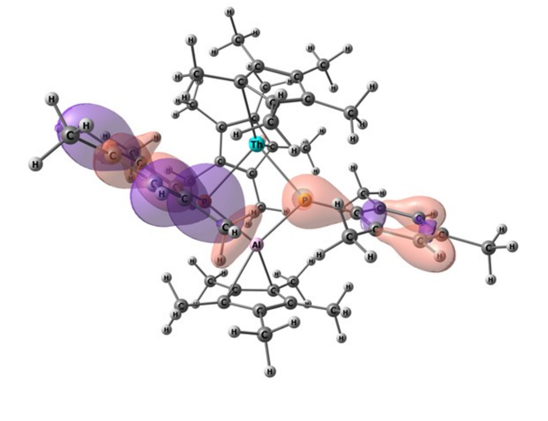

(102) (-0.53115 a.u.)

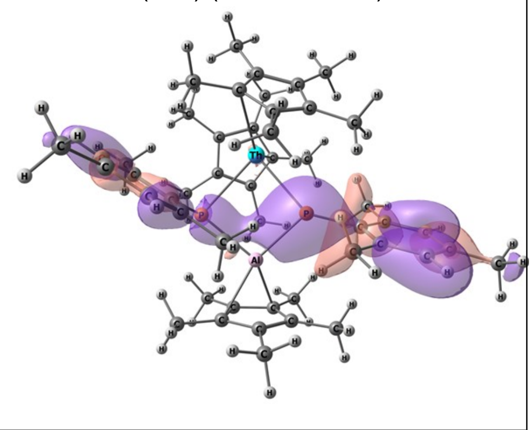

(98) (-0.58296 a.u.)

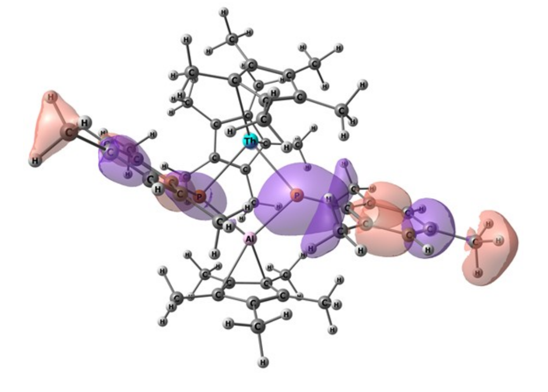

Table S9. Computed BCP descriptors

	ρ(r)	$^2\rho$(r)	G(r)	V(r)	H(r)	ε
Th1-P2	0.101	0.211	0.085	-0.117	-0.322	0.222
Th1-P3	0.101	0.212	0.085	-0.117	-0.032	0.223
Al4-P2	0.079	0.531	0.038	-0.062	-0.024	0.095
Al4-P3	0.079	0.530	0.038	-0.062	-0.024	0.097
Th1-P3- Al2-P4	0.301	0.117	0.029	-0.293	-0.00001	-1.445

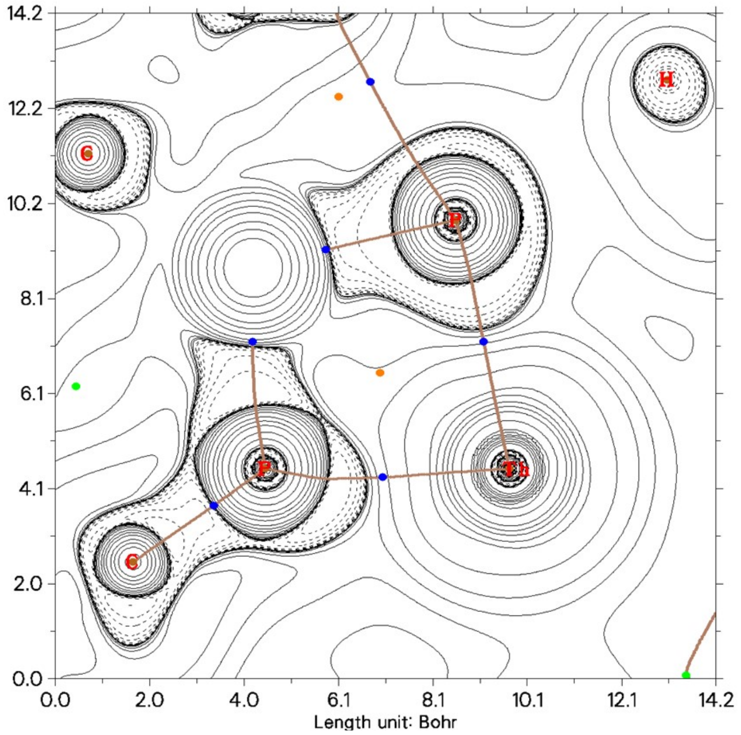

Compound 2

Table 10. Comparison of selected bond distances between DFT optimized structures and X-ray for Th-As-Al

(S=3/2)	DFT		X-ray	
	dispersion	No dispersion		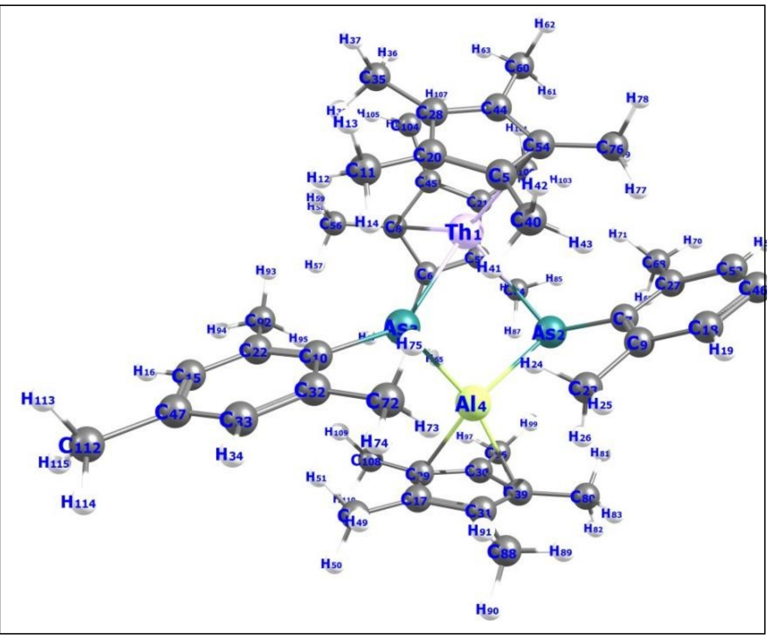
Th 1-As2	2.98636	3.02407	2.98137	
Th 1-As3	2.85542	2.92479	2.84611	
Al4-As2	2.50094	2.52842	2.46304	
Al4-As3	2.39647	2.43817	2.37570	
Th 1-X120	2.51891	2.58379	2.54275	
Th 1-X121	2.53744	2.55134	2.52421	

Table S11: Computed natural charges

Atom labels	Natural charges
Th1	0.76204
As2	-0.20728
As3	-0.21583
Al4	1.50956

Table S12: Computed Wiberg bond index between selected atoms

Atom labels	Wiberg bond index
Th1- As2	1.0760
Th1- As3	1.2853
Al4- As2	0.6712
Al4- As3	0.7470

Table S13: NBO bond analysis

Bond	% Contribution	Orbitals			
		s	p	d	f
(1.84795) BD (1)Th 1-As 2	(25.97%) 0.5096*Th 1	22.20%	0.06%	66.61%	11.12%
	(74.03%) 0.8604*As 2	22.48%	77.31 %	0.21%	-
(1.90755) BD (1)Th 1-As 3	(21.81%) 0.4670*Th 1	19.99%	0.10%	62.69%	17.20%
	(78.19%) 0.8843*As 3	49.30%	50.64 %	0.06%	-
(1.83892) BD (2)Th 1-As 3	(20.37%) 0.4513*Th 1	0.06%	0.14%	58.37%	41.42%
	(79.63%) 0.8924*As 3	1.76%	98.19 %	0.05%	-
(1.73649) BD (1)As 2-Al 4	(76.23%) 0.8731*As 2	13.08%	86.61 %	0.32%	
	(23.77%) 0.4876*Al 4	46.94%	51.98 %	1.07%	
(1.80992) BD (1)As 3-Al 4	(78.38%) 0.8853*As 3	28.07%	71.74 %	0.18%	
	(21.62%) 0.4650*Al 4	43.81%	55.14 %	1.04%	
(0.08558) BD*(1)Th 1-As 2	(74.03%) 0.8604*Th 1	22.20%	0.06%	66.61%	11.12%
	(25.97%) -0.5096*As 2	22.48%	77.31 %	0.21%	
(0.08414) BD*(1)Th 1-As 3	(78.19%) 0.8843*Th 1	19.99%	0.10%	62.69%	17.20%
	(21.81%) -0.4670*As 3	49.30%	50.64 %	0.06%	
(0.06316) BD*(2)Th 1-As 3	(79.63%) 0.8924*Th 1	0.06%	0.14%	58.37%	41.42%
	(20.37%) -0.4513*As 3	1.76%	98.19 %	0.05%	
(0.11151) BD*(1)As 2-Al 4	(23.77%) 0.4876*As 2	13.08%	86.61 %	0.32%	
	(76.23%) -0.8731*Al 4	46.94%	51.98 %	1.07%	
(0.09853) BD*(1)As 3-Al 4	(21.62%) 0.4650*As 3	28.07%	71.74 %	0.18%	
	(78.38%) -0.8853*Al 4	43.81%	55.14 %	1.04%	

Table S14: NBO second-order perturbation analysis

Donor NBO	Acceptor NBO	E(2) kcal/mol
(1.98492) CR (2)Th1<br>s(100.00%)	(0.22560) LV(2)Al4<br>s(8.05%)p$^{11.42}$(91.88%)d$^{0.01}$(0.08%)	13.14
62. (1.76256) LP (1)As2<br>sp$^{0.90}$	(0.09853) BD*(1)As3-Al4<br>As3 s(28.07%)p$^{2.56}$(71.74%)d$^{0.01}$(0.18%)-<br>Al 4 s(43.81%)p$^{1.26}$(55.14%)d$^{0.02}$(1.04%)	10.29
(1.84795) BD (1)Th1-As2<br>Th 1 (sd$^{3.00}$f$^{0.50}$)-As2 (sp$^{3.44}$)	210. (0.25207) LV 1)Al4<br>s(0.47%)p$^{99.99}$(99.40%)d$^{0.27}$(0.13%)	18.30
(1.84795) BD (1)Th1-As2<br>Th1 (sd$^{3.00}$f$^{0.50}$)-As2 (sp$^{3.44}$)	(0.22560) LV (2)Al4<br>s(8.05%)p$^{11.42}$(91.88%)d$^{0.01}$(0.08%)	39.14
(1.90755) BD (1)Th1-As3<br>Th1 (sd$^{3.14}$f$^{0.86}$)-As3 (sp$^{1.03}$)	(0.25207) LV (1)Al4<br>s(0.47%)p$^{99.99}$99.40%)d$^{0.27}$(0.13%)	17.09
(1.90755) BD 1)Th1-As3<br>Th1 (sd$^{3.14}$f$^{0.86}$)-As3 (sp$^{1.03}$)	(0.22560) LV (2)Al4<br>s(8.05%)p$^{11.42}$(91.88%)d$^{0.01}$(0.08%)	34.82
(1.83892) BD (2)Th1-As3<br>Th1 (sp$^{2.27}$d$^{99.99}$f$^{99.99}$)-As3<br>(sp$^{55.69}$)	(0.25207) LV (1)Al4<br>s(0.47%)p$^{99.99}$(99.40%)d$^{0.27}$(0.13%)	12.54
(1.73649) BD (1)As2-Al4<br>As2 (sp$^{6.62}$)-Al4 (sp$^{1.11}$)	(0.37010) LV (1)Th1<br>sp$^{0.24}$(0.02%)d$^{99.99}$(81.58%)f$^{99.99}$(18.31%)	37.56
(1.73649) BD (1)As2-Al4<br>As2 (sp$^{6.62}$)-Al4 (sp$^{1.11}$)	(0.29299) LV (2)Th1<br>s(1.51%)p$^{0.01}$(0.02%)d$^{52.87}$(79.59%)f$^{12.54}$(18.87%)	12.73
(1.73649) BD (1)As2-Al4<br>As2 (sp$^{6.62}$)-Al4 (sp$^{1.11}$)	(0.21364) LV (4)Th1<br>s(34.86%)d$^{1.15}$(39.99%)f $^{0.72}$(25.12%)	18.66
(1.73649) BD (1)As2-Al4<br>As2 (sp$^{6.62}$)-Al4 (sp$^{1.11}$)	(0.14553) LV (5)Th1<br>s(2.71%)p$^{0.06}$(0.17%)d$^{3.10}$(8.40%)f$^{32.76}$(88.72%)	15.08
(1.73649) BD (1)As 2-Al 4<br>As2 (sp$^{6.62}$)-Al4 (sp $^{1.11}$)	(0.11760) LV (6)Th1<br>s(2.05%)p$^{0.13}$(0.27%)d$^{4.01}$(8.21%)f$^{43.67}$(89.45%)	12.91
(1.73649) BD (1)As2-Al4<br>As2 (sp$^{6.62}$)-Al4 (sp$^{1.11}$)	(0.08820) LV (7)Th1<br>s(0.23%)p$^{0.87}$(0.20%)d$^{18.35}$(4.23%)f$^{99.99}$(95.29%)	17.03
(1.73649) BD (1)As2-Al4<br>As2 (sp$^{6.62}$)-Al4 (sp$^{1.11}$)	(0.06556) LV (9)Th1<br>s(1.41%)p$^{0.24}$(0.34%)d$^{27.11}$(38.27%)f$^{42.46}$(59.95%)	12.48
(1.73649) BD (1)As2-Al4<br>As2 (sp$^{6.62}$)-Al4 (sp$^{1.11}$)	(0.08558) BD*(1)Th1-As2<br>Th1 s(22.20%)d$^{3.00}$(66.61%)f$^{0.50}$(11.12%)-<br>As2 s(22.48%)p$^{3.44}$(77.31%)	18.99
(1.80992) BD (1)As3-Al4<br>As3 (sp$^{2.56}$)-Al4 (sp$^{1.26}$)	(0.37010) LV (1)Th1<br>s(0.08%)p$^{0.24}$(0.02%)d$^{99.99}$(81.58%)f$^{99.99}$(18.31%)	44.56
(1.80992) BD (1)As3-Al4<br>As3 (sp$^{2.56}$)-Al4 (sp$^{1.26}$)	201. (0.29299) LV (2)Th 1<br>s(1.51%)d$^{52.87}$(79.59%)f$^{12.54}$(18.87%)	14.63
(1.80992) BD (1)As3-Al4<br>As3 (sp$^{2.56}$)-Al4 (sp$^{1.26}$)	(0.21364) LV (4)Th1<br>s(34.86%)d$^{1.15}$(39.99%)f$^{0.72}$(25.12%)	17.89
(1.80992) BD (1)As3-Al4<br>As3 (sp$^{2.56}$)-Al4 (sp$^{1.26}$)	(0.14553) LV (5)Th1<br>s(2.71%)p$^{0.06}$(0.17%)d$^{3.10}$(8.40%)f$^{32.76}$(88.72%)	18.40
(1.80992) BD (1)As3-Al4<br>As3 (sp$^{2.56}$)-Al4 (sp$^{1.26}$)	(0.11760) LV (6)Th1<br>s(2.05%)p$^{0.13}$(0.27%)d$^{4.01}$(8.21%)f$^{43.67}$	54.93
(1.80992) BD (1)As3-Al4<br>As3 (sp$^{2.56}$)-Al4 (sp$^{1.26}$)	(0.08414) BD*(1)Th1-As3<br>Th1 s(19.99%)d$^{3.14}$(62.69%)f$^{0.86}$(17.20%)-<br>As3 s(49.30%)p$^{1.03}$(50.64%)	36.78
(1.80992) BD (1)As3-Al4<br>As3 (sp$^{2.56}$)-Al4 (sp$^{1.26}$)	(0.08820) LV (7)Th1<br>s(0.23%)p$^{0.87}$(0.20%)d$^{18.35}$(4.23%)f$^{99.99}$(95.29%)	17.46
(1.80992) BD (1)As3-Al4<br>As3 (sp$^{2.56}$)-Al4 (sp$^{1.26}$)	(0.01281) RY (5)Th1<br>s(0.90%)p$^{1.26}$(1.14%)d$^{69.16}$(62.56%)f39.09(35.36%)	11.92

Table S15: Computed MOs.

(199)HOMO (-0.15585 a.u)	(198) (-0.16297 a.u.)
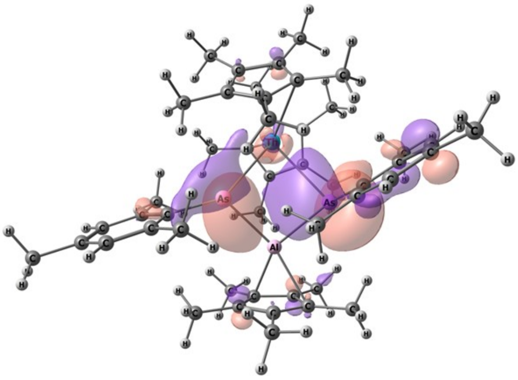	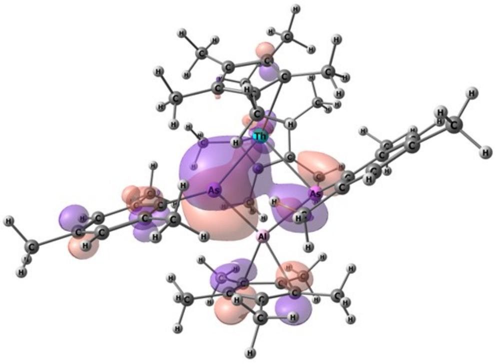
(197) (-0.16967 a.u.)	(194) (-0.21221 a.u.)
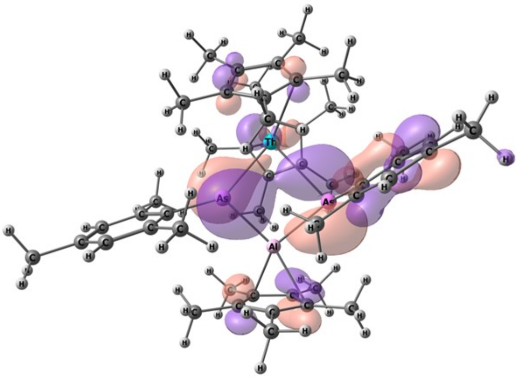	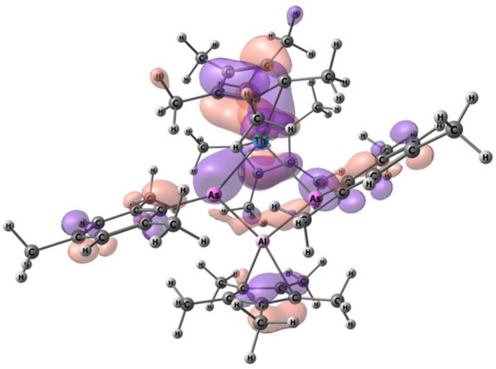
(187) (-0.23894 a.u.)	(186) (-0.25077 a.u.)
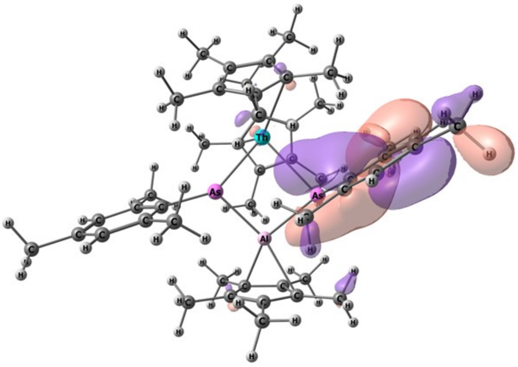	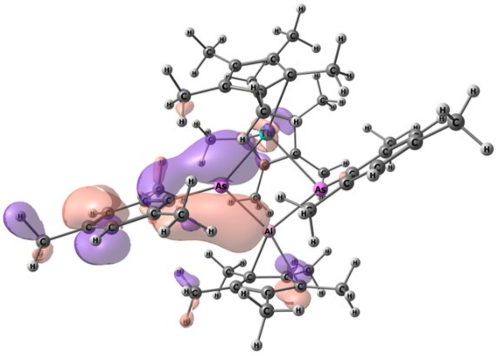
(103) (-0.52210 a.u.)	(102) (-0.53294 a.u.)
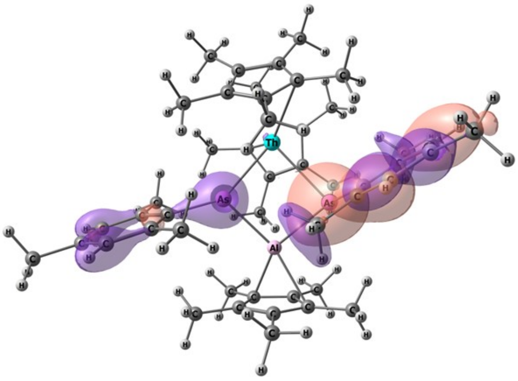	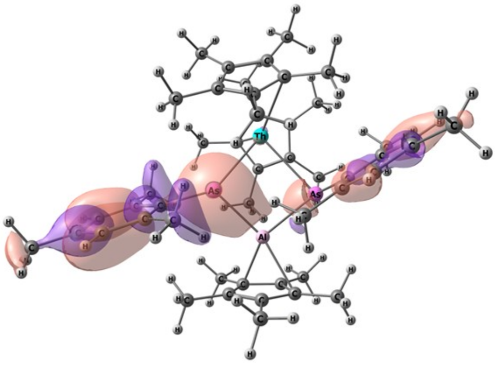
(98) (-0.58108 a.u.)	

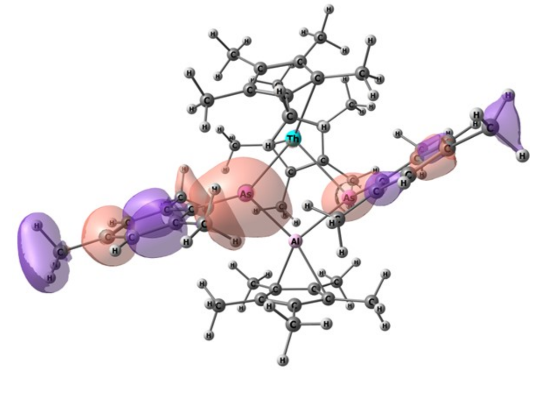

Table S16: Computed BCP descriptors

	ρ(r)	$^2\rho$(r)	G(r)	V(r)	H(r)	ε
Th1- As2	0.053	0.019	0.019	-0.034	-0.015	0.158
Th1- As3	0.054	0.052	0.027	-0.041	-0.015	0.451
Al4- As2	0.053	0.067	0.008	-0.036	-0.027	0.101
Al4- As3	0.056	0.108	0.011	-0.028	-0.028	0.133
Th1-As2- Al4-As3	0.017	0.038	0.010	-0.010	- 0.0004	-1.382

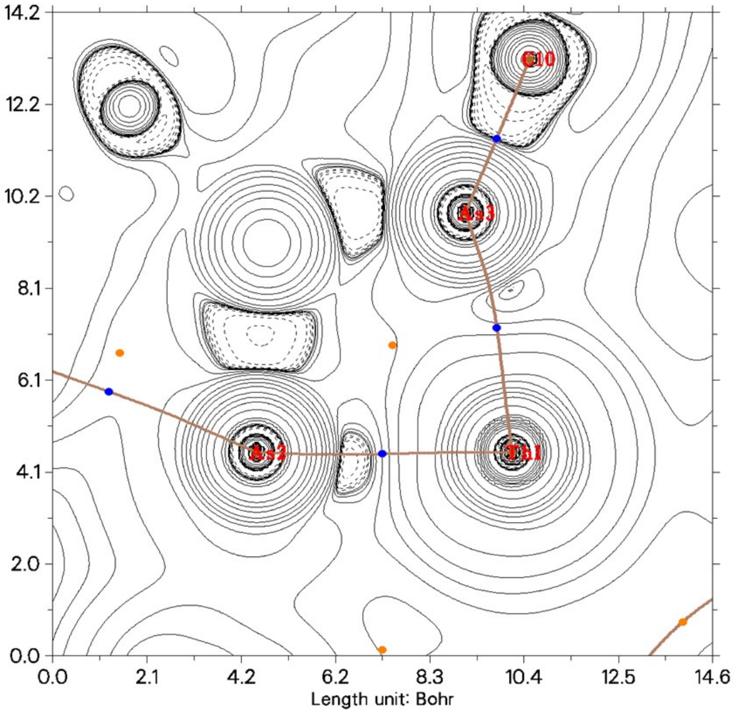

Compound 3

Table S17. Comparison of selected bond distances between DFT optimized structures and X-ray for U-P-Al

(S=3/2)	DFT		X-ray
	dispersion	No dispersion	
U1-P2	2.70079	2.78493	2.73270
U1-P3	2.69255	2.78673	2.69532
Al4-P2	2.36650	2.37589	2.32901
Al4-P3	2.34138	2.36789	2.31249
U1-X120	2.42773	2.52876	2.49792
U1-X121	2.47568	2.48088	2.44962

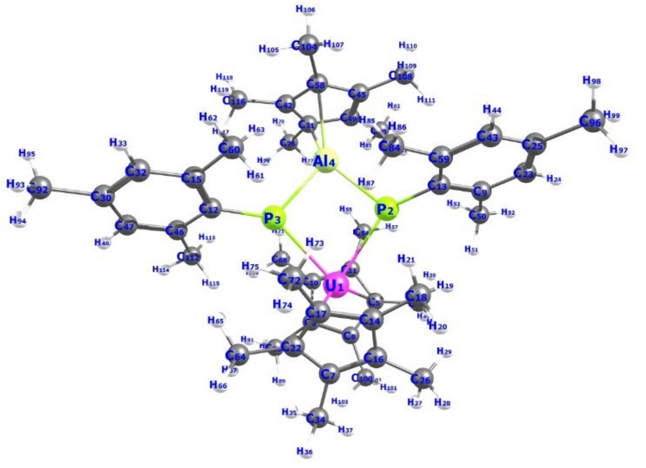

Table S18. Computed natural charges

Atom labels	Natural charges	Spin Density
U1	0.74296	2.27271
P2	-0.29491	-0.05373
P3	-0.22987	-0.12280
Al4	1.55174	0.00220

Table S19. Computed Wiberg bond index between selected atoms

Atom labels	Wiberg bond index
U1-P2	1.0379
U1-P3	1.2577
Al4-P2	0.7125
Al4-P3	0.7062

Alpha MOs

Table S20. NBO bond analysis

Bond	% Contribution	Orbitals			
		s	p	d	f
(0.94442) BD(1) U1-P2	(15.93%)0.3991*U1	9.41%	0.16%	53.43%	37.00%
	(84.07%) 0.9169*P2	37.92%	61.94%	0.15%	-
(0.90657) BD(2) U1- P2	(23.49%) 0.4847*U1	8.71%	0.07%	56.94%	34.27%
	(76.51%) 0.8747*P2	3.62%	96.30%	0.07%	-
((0.95099) BD(1) U1- P3	(24.28%) 0.4927*U1	18.38%	0.09%	62.37%	19.15%
	(75.72%) 0.8702*P3	41.74%	58.17%	0.09%	-
(0.91502) BD(2) U1- P3	(29.56%) 0.5437*U1	0.43%	0.07%	37.44%	62.07%)
	(70.44%) 0.8393*P3	1.14%	98.81%	0.05%	
(0.92398) BD (1) P2-Al4	(78.01%) 0.8832*P2	35.74%	63.95%	0.31	
	(21.99%) 0.4689*Al4	42.65%	56.38%	0.97%	

(0.93808) BD (1) P3-Al4	(80.6%) 0.8970*P3	32.24%	67.58%	0.18%	
	(19.54%) 0.4421*Al4	46.24%	52.77%	0.99%	
(0.04549) BD*(1) U1- P2	(84.07%) 0.9169*U1	9.41%	0.16%	53.43%	37.00%
	(15.93%) -0.3991*P2	37.92%	61.94%	0.15%	
(0.06755) BD*(2) U1- P2	(76.51%) 0.8747*U1	8.71%	0.07%	56.94%	34.27%
	(23.49%) -0.4847*P2	3.62%	96.30%	0.07%	
(0.05236) BD*(1) U1- P3	(75.72%) 0.8702*U1	18.38%	0.09%	62.37%	19.15%
	(24.28%) -0.4927*P3	41.74%	58.17%	0.09%	
(0.04100) BD*(2) U1- P3	(70.44%) 0.8393*U1	0.43%	0.07%	37.44%	62.07%
	(29.56%) -0.5437*P3	1.14%	98.81%	0.05%	
(0.03677) BD*(1) P2-Al4	(21.99%) 0.4689*P2	35.74%	63.95%	0.31%	
	(78.01%) - 0.8832*Al4	42.65%	56.38%	0.97%	
(0.05381) BD*(1) 3-Al4	(19.54%) 0.4421* P3	32.24	67.58%	0.18%	
	(80.46%) - 0.8970*Al4	46.24%	52.77%	0.99%	

Table S21. NBO second-order perturbation analysis

Donor NBO	Acceptor NBO	E(2) kcal/mol
(0.90657) BD (2) U1- P2 U1 (sp$^{0.01}$d$^{6.54}$f$^{3.94}$)-P2 (sp$^{26.58}$d$^{0.02}$)	(0.11601) LV (2)Al4 s(6.75%)p$^{13.80}$(93.19%)d$^{0.01}$(0.06%)	18.20
(0.95099) BD (1) U1- P3 U1 (sp$^{0.01}$d$^{3.39}$f$^{1.04}$)-P3 (sp$^{1.39}$)	(0.11601) LV (2)Al4 s(6.75%)p$^{13.80}$(93.19%)d $^{0.01}$(0.06%)	13.75

Table S22. Computed Alpha MOs.

(201)HOMO (-0.15739 a.u.)	(200) (-0.17009 a.u.)
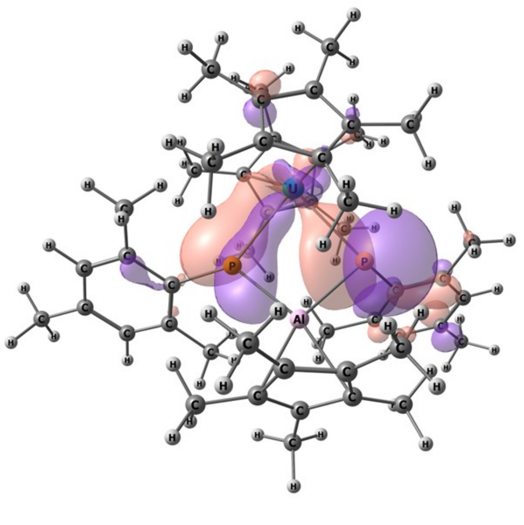	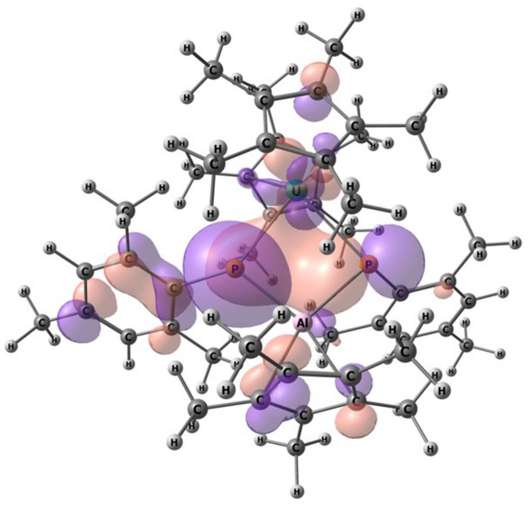
(199) (-0.17413 a.u.)	(193) (-0.21591 a.u.)

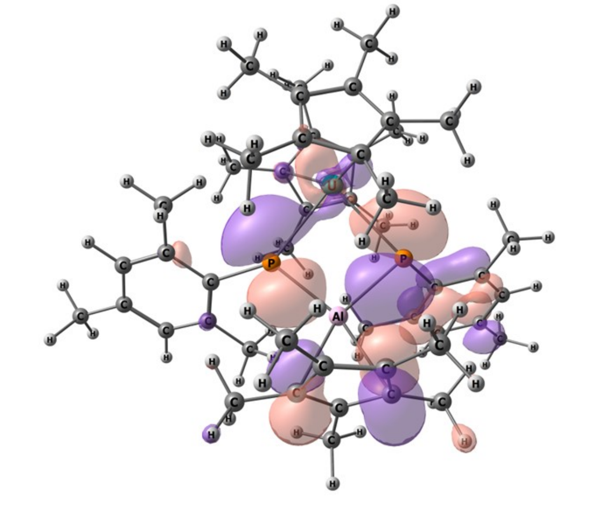	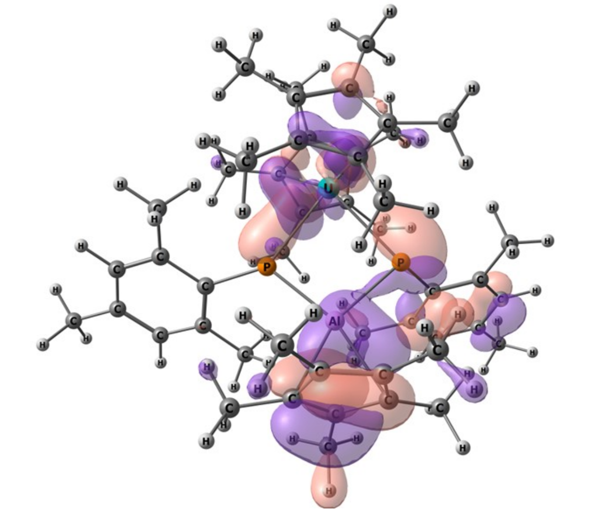
(188) (-0.23202 a.u.)	(187) (-0.25032 a.u.)
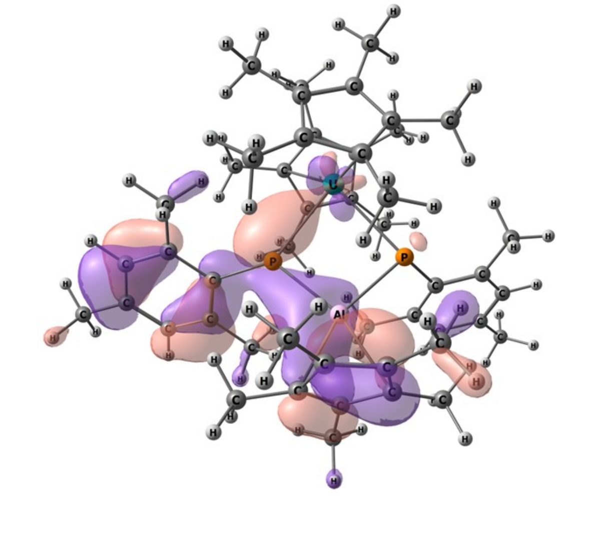	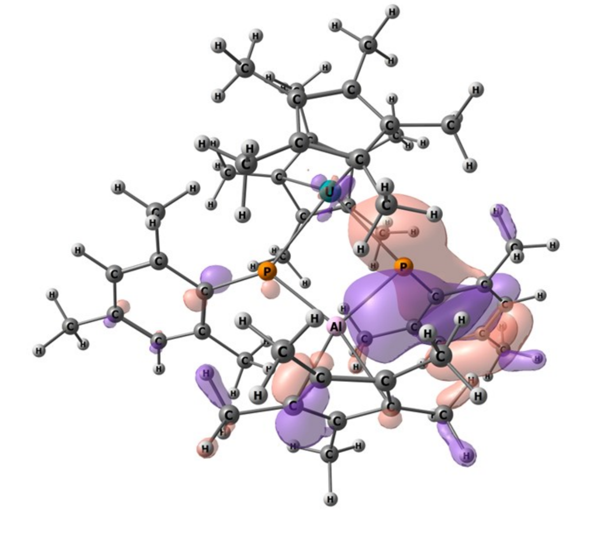
(186) (-0.25448 a.u.)	(103) (-0.52112 a.u.)
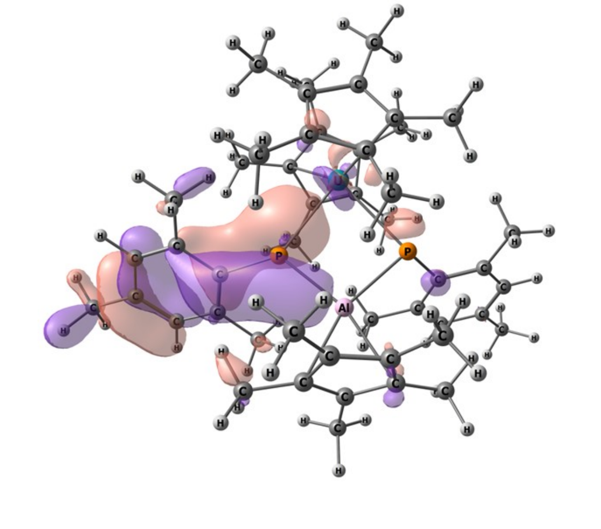	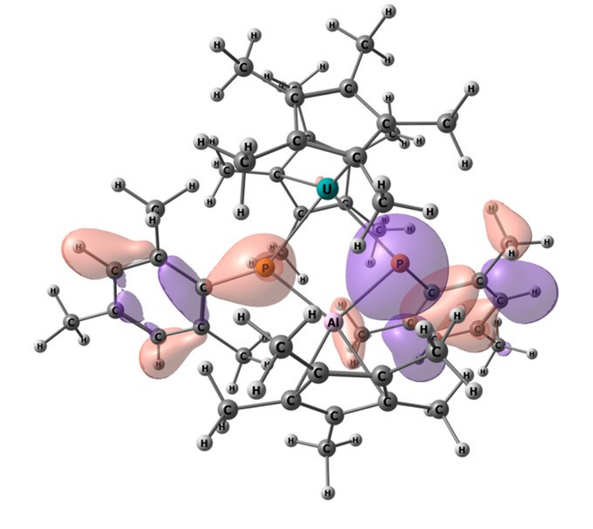
(102) (-0.53285 a.u.)	(98) (-0.58465 a.u.)

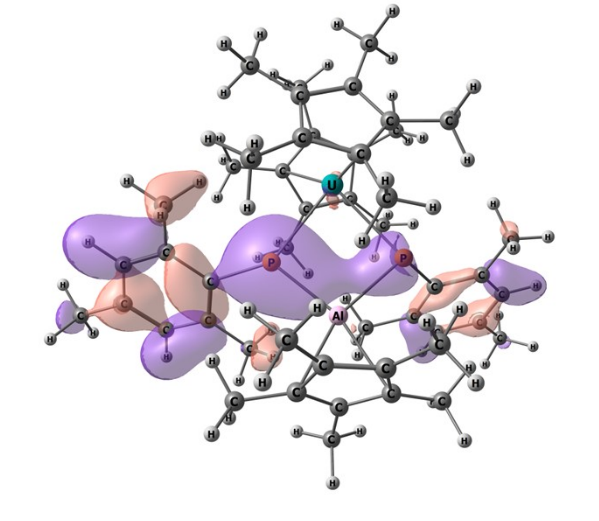 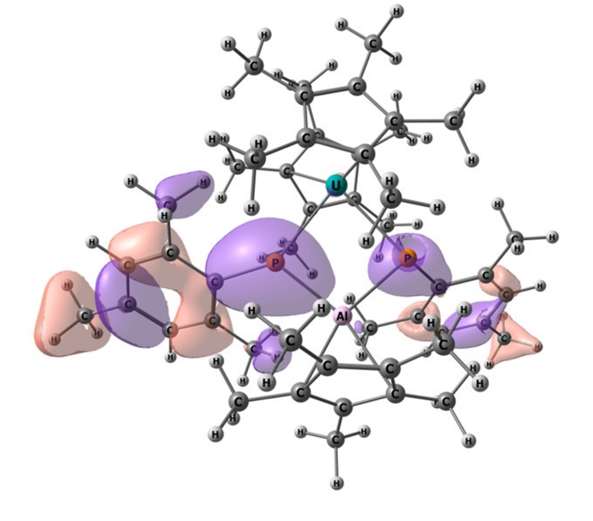

Beta MOs

Table S23. NBO bond analysis

Bond	% Contribution	Orbitals			
		s	p	d	f
(0.92849) BD (1) U1- P2	(25.00%) 0.5000* U1	19.47%	0.08%	62.29%	18.16 %
	(75.00%) 0.8660* P2	26.79%	73.10%	0.12%	-
(0.95021) BD (1) U1- P3	(22.34%) 0.4726* U1	18.94%	0.11%	61.52%	19.43 %
	(77.66%) 0.8813* P3	43.53%	56.36%	0.07%	-
(0.91152) BD (2) U1- P3	(17.13%) 0.4139* U1	0.69%	0.10%	50.50%	48.70 %
	(82.87%) 0.9103* P3	1.97%	98.00%	0.03%	-
(0.91125) BD (1) P2-Al4	(77.67%) 0.8813* P2	19.23%	80.44%	0.33%	
	(22.33%) 0.4725* Al4	49.28%	49.53%	1.19%	
(0.93635) BD (1) P3-Al4	(80.95%) 0.8997* P3	30.24%	69.60%	0.16%	
	(19.05%) 0.4365* Al4	42.03%	56.81%	1.16%	
(0.05300) BD*(1) U1- P2	(75.00%) 0.8660* U1	19.47%	0.08%	62.29%	18.16 %
	(25.00%) -0.5000* P2	26.79%	73.10%	0.12%	
(0.04836) BD*(1) U1- P3	(77.66%) 0.8813* U1	18.94%	0.11%	61.52%	19.43 %
	(22.34%) -0.4726* P3	43.57%	56.36%	0.07%	
(0.02781) BD*(2) U1- P3	(82.87%) 0.9103* U1	0.69%	0.10%	50.50%	48.70 %
	(17.13%) -0.4139* P3	1.97%	98.00%	0.03%	
(0.04565) BD*(1) P2- Al4	(22.33%) 0.4725* P2	19.23%	80.44%	0.33%	
	(77.67%) -0.8813*Al4	49.28%	49.53%	1.19%	
(0.05039) BD*(1) P 3- Al4	(19.05%) 0.4365* P3	30.24%	69.60%	0.16%	
	(80.95%) -0.8997*Al4	42.03%	56.81%	1.16%	

Table S24. NBO second-order perturbation analysis

Donor NBO	Acceptor NBO	E(2) kcal/mol
(0.92849) BD (1) U1- P2<br>U1 (sd$^{3.20}$f$^{0.93}$)-P2 (sp$^{2.73}$)	(0.11490) LV(2)Al4<br>s(6.97%)p$^{13.33}$(92.98%)d$^{0.01}$(0.05%)	24.43
(0.95021) BD (1) U1- P3<br>U1 (sd$^{3.25}$f $^{1.03}$)-P3 (sp$^{1.29}$)	211. (0.11490) LV(2)Al4<br>s(6.97%)p$^{13.33}$(92.98%)	15.13

Table S25. Computed Beta MOs.

(199)HOMO (-0.15655 a.u.)<br>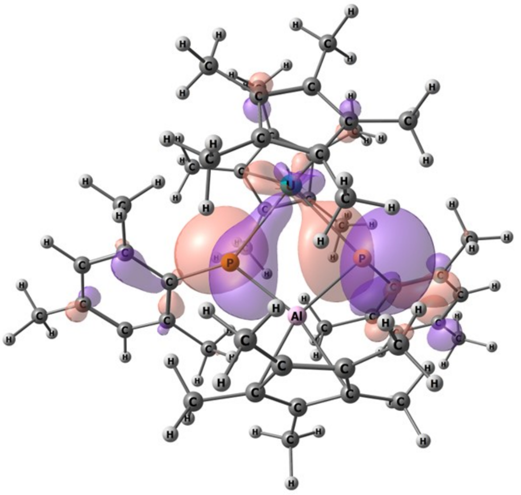	(198) (-0.17295 a.u.)<br>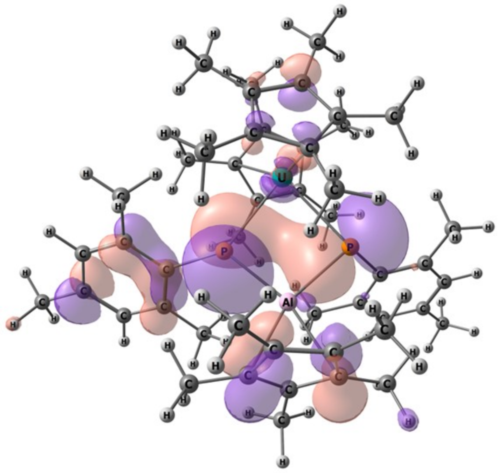
(197) (-0.17514 a.u.)<br>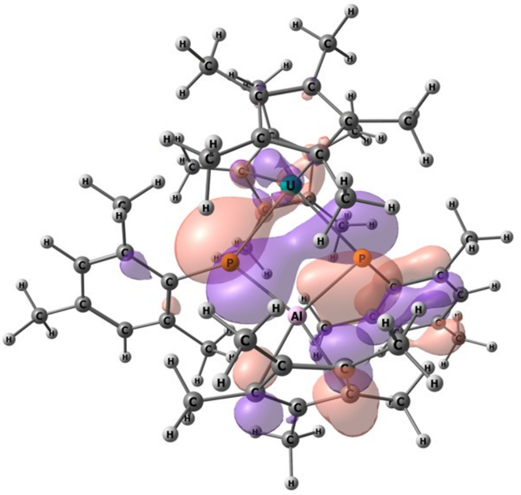	(189) (-0.22905 a.u.)<br>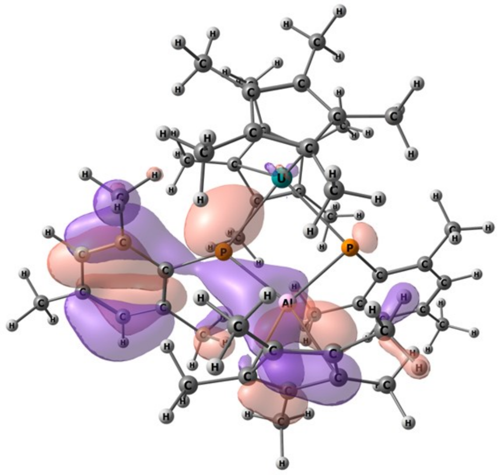
(188) (-0.23176 a.u.)	(187) (-0.24995 a.u.)

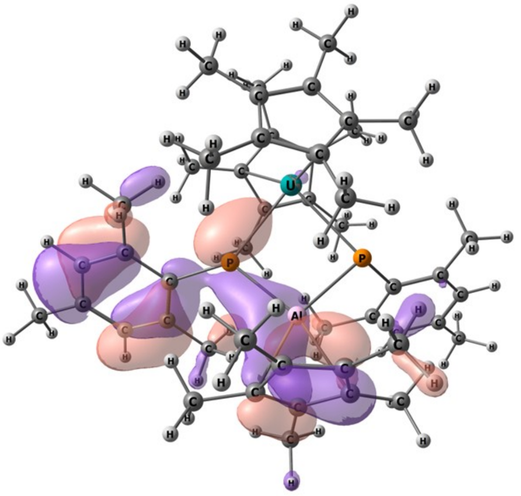	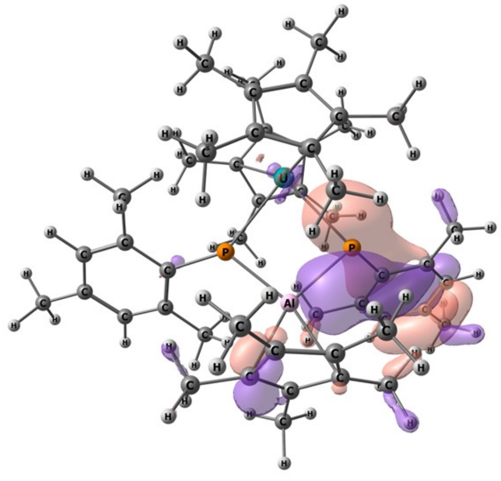
(186) (-0.25412 a.u.)	(185) (-0.28023 a.u.)
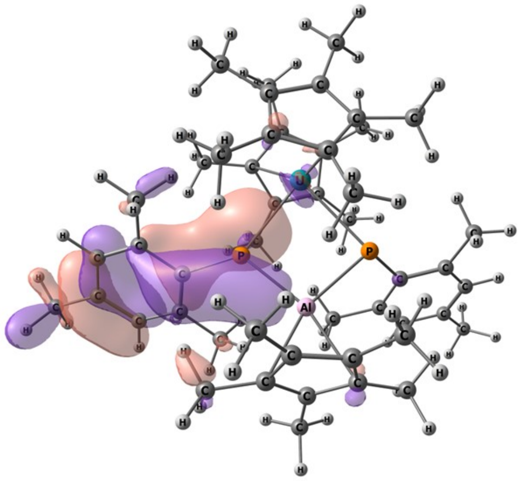	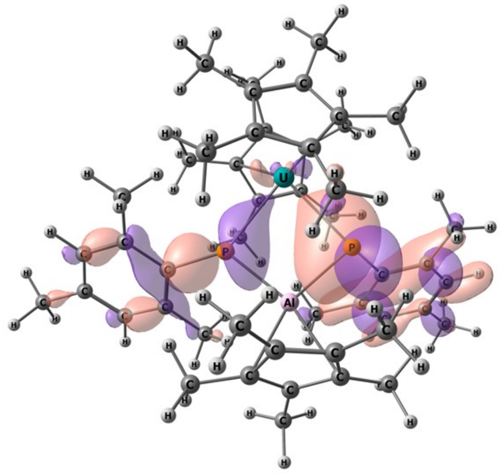
(103) (-0.52176 a.u.)	(102) (-0.53371 a.u.)
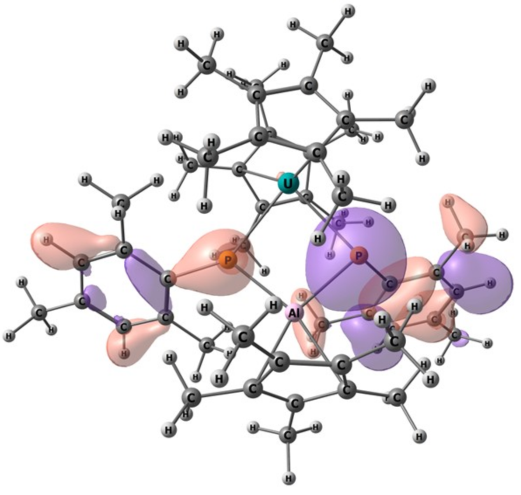	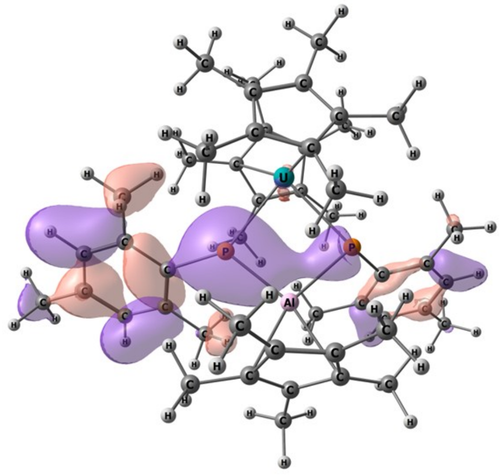
(99) (-0.57591 a.u.)	(98) (-0.58573 a.u.)

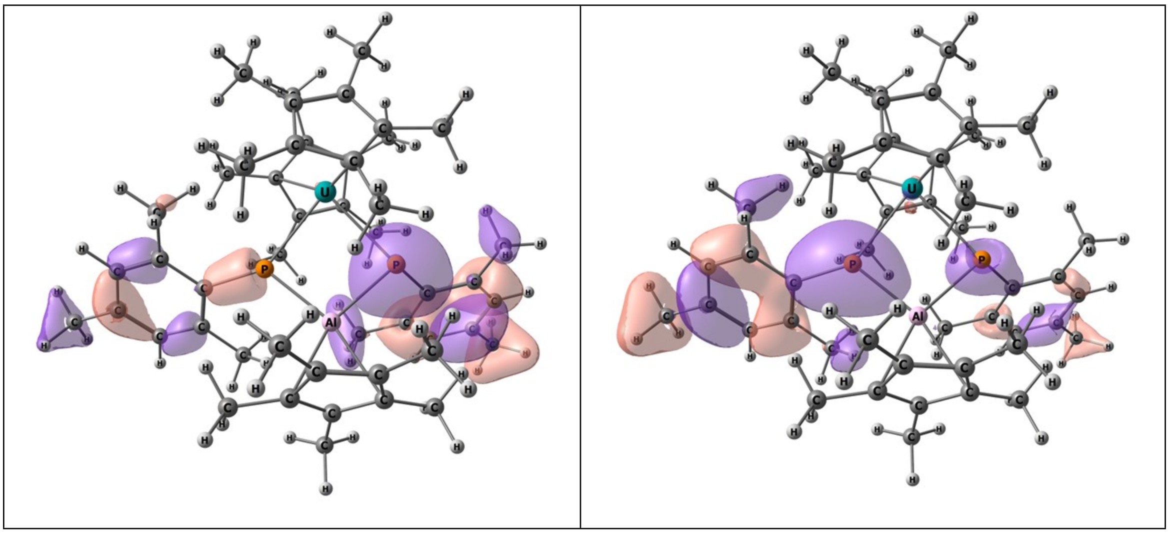

Table S26: Computed BCP descriptors

	ρ(r)	$^{2}\rho$(r)	G(r)	V(r)	H(r)	ε
U1-P2	0.060	0.046	0.028	-0.044	-0.016	0.167
U1-P3	0.067	0.068	0.037	-0.056	-0.020	0.498
Al4-P2	0.059	0.120	0.011	-0.040	-0.029	0.086
Al4-P3	0.056	0.140	0.012	-0.038	-0.026	0.090
U1-P3-Al4-N2	0.020	0.043	0.012	-0.013	-0.001	-1.55

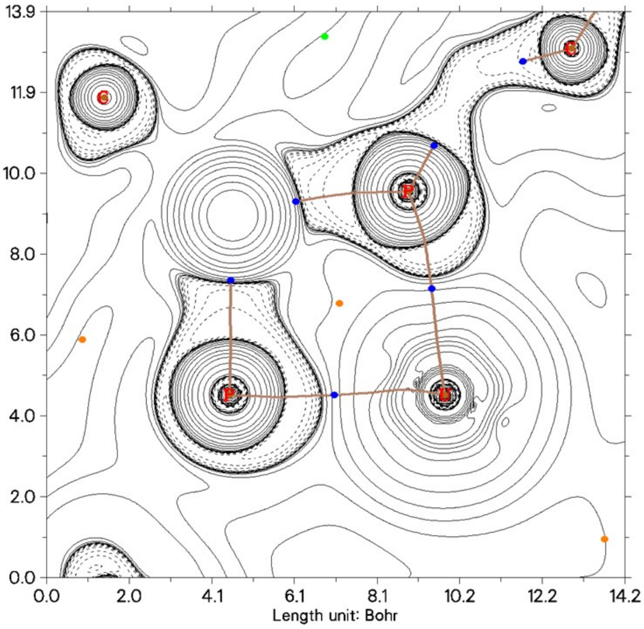

Compound 4

Table 27. Comparison of selected bond distances between DFT optimized structures and X-ray for U-As-Al

(S=3/2)	DFT		X-ray	
	dispersion	No dispersion		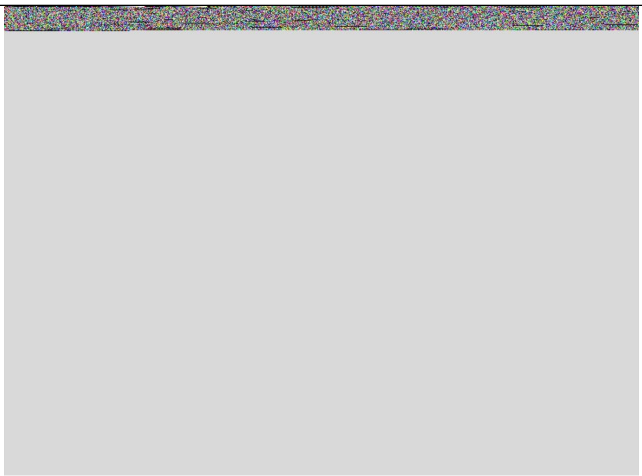
U1-As2		3.05565	-	
U1-As3		2.84354	-	
Al4-As2		2.48489	-	
Al4-As3		2.49082	-	
U1-X120		2.47756	-	
U1-X121		2.51367	-	

Table S28: Computed natural charges for **UAsAl**

Atom labels	Natural charges	Spin Density
U1	0.84173	2.32557
As2	-0.29712	-0.02929
As3	-0.15860	-0.16762
Al4	1.44153	-0.00422

Table S29: Computed Wiberg bond index between selected atoms in **UAsAl**

Atom labels	Wiberg bond index
U1-As2	0.8150
U1-As3	1.2576
Al4-As2	0.7858
Al4-As3	0.7534

Table S30: NBO bond analysis for **UAsAl**

Alpha

Bond	% Contribution	Orbitals			
		s	p	d	f
69. (0.90937) BD (1) U1-As2	(27.54%) 0.5248* U1	22.67%	0.16%	64.96%	12.21%
	(72.46%) 0.8512* As2	15.45%	84.41%	0.15%	-
70. (0.95084) BD (1) U1-As3	(24.43%) 0.4943* U1	20.86%	0.10%	62.21%	16.84%
	(75.57%) 0.8693*As3	43.53%	56.37%	0.09%	-
71. (0.92053) BD (2) U1-As3	(35.76%) 0.5980* U1	0.52%	0.05%	27.81%	71.63%
	(64.24%) 0.8015*As3	0.84%	99.12%	0.04%	-
72. (0.92114) BD (1)As2-Al4	(74.49%) 0.8631*As2	15.00%	84.71%	0.29%	
	(25.51%) 0.5051*Al4	51.61%	47.45%	0.94%	
74. (0.94331) BD (1)As3-A4	(79.20%) 0.8899*As3		66.57%	0.16%	

		33.27%			
	(20.80%) 0.4561*Al4	39.26%	59.75%	0.99%	
213. (0.06518) BD*(1) U1-As2	(72.46%) 0.8512* U1	22.67%	0.16%	64.96%	12.21%
	(27.54%) -0.5248*As2	15.45%	84.41%	0.15%	
214. (0.05419) BD*(1) U1-As3	(75.57%) 0.8693* U1	20.86%	0.10%	62.21%	16.84%
	(24.43%) -0.4943*As3	43.53%	56.37%	0.09%	
215. (0.05582) BD*(2) U1-As3	(64.24%) 0.8015* U1	0.52%	0.05%	27.81%	71.63%
	(35.76%) -0.5980*As3	0.84%	99.12%	0.04%	
216. (0.05195) BD*(1)As2-Al4	(25.51%) 0.5051*As2	15.00%	84.71%	0.29%	
	(74.49%) -0.8631*Al4	51.61%	47.45%	0.94%	
218. (0.05067) BD*(1)As3-Al4	(20.80%) 0.4561*As3	33.27%	66.57%	0.16%	
	(79.20%) -0.8899*Al4	39.26%	59.75%	0.99%	

Beta MOs

Bond	% Contribution	Orbitals			
		s	p	d	f
(0.91624) BD (1) U 1-As 2	(25.40%) 0.5039* U 1	23.13%	0.12%	59.82%	16.93 %
	(74.60%) 0.8637*As 2	17.92%	81.94 %	0.13%	-
(0.95093) BD (1) U 1-As 3	(22.48%) 0.4741* U 1	21.04%	0.11%	61.76%	17.08 %
	(77.52%) 0.8805*As 3	46.02%	53.52 %	0.07%	-
(0.91159) BD (2) U 1-As 3	(16.85%) 0.4104* U 1	0.35%	0.08%	52.42%	47.15 %
	(83.15%) 0.9119*As 3	1.29%	98.68 %	0.03%	-
(0.92538) BD (1)As 2-Al 4	(74.44%) 0.8628*As 2	15.50%	84.22 %	0.22%	
	(25.56%) 0.5056*Al 4	51.47%	47.61 %	0.92%	
(0.94064) BD (1)As 3-Al 4	(79.16%) 0.8897*As 3	30.73%	69.13 %	0.14%	
	(20.84%) 0.4565*Al 4	39.83%	59.21 %	0.95%	
(0.05084) BD*(1) U 1-As 2	(74.60%) 0.8637* U 1	23.13%	0.12%	59.82%	16.93 %
	(25.40%) -0.5039*As 2	17.92%	81.94 %	0.13%	
(0.04903) BD*(1) U 1-As 3	(77.52%) 0.8805* U 1	21.04%	0.11%	61.76%	17.08 %
	(22.48%) -0.4741*As 3	46.02%	53.92 %	0.07%	
(0.02631) BD*(2) U 1-As 3	(83.15%) 0.9119* U 1	0.35%	0.08%	52.42%	47.15 %
	(16.85%) -0.4104*As 3	1.29%	98.68 %	0.03%	
(0.05069) BD*(1)As 2-Al 4	(25.56%) 0.5056*As 2	15.50%	84.22 %	0.29%	
	(74.44%) -0.8628*Al 4	51.47%	47.61 %	0.92%	
(0.05270) BD*(1)As 3-Al 4	(20.84%) 0.4565*As 3	30.73%	69.13 %	0.14%	
	(79.16%) -0.8897*Al 4	39.83%	59.21	0.95%	

			%		

Table S31: NBO second order perturbation analysis

Alpha MOs

Donor NBO	Acceptor NBO	E(2) kcal/mol
(0.90937) BD (1) U1-As2<br>U 1 (sp0.01d$^{2.87}$f$^{0.54}$)-As2 (sp$^{5.46}$)	(0.12773) LV (1)Al 4<br>s(3.38%)p$^{28.54}$(96.56%)d $^{0.02}$(0.05%)	20.01
(0.90937) BD (1) U 1-As 2<br>U 1 (sd$^{2.87}$f $^{0.54}$)-As2 (sp$^{5.46}$)	(0.11587) LV (2)Al4<br>s(4.93%)p$^{19.26}$(94.96%)d$^{0.02}$(0.11%)	15.50
(0.95084) BD (1) U1-A3<br>U1 (sd$^{2.98}$f$^{0.81}$)-As3 (sp$^{1.29}$)	(0.12773) LV (1)Al4<br>s(3.38%)p$^{28.54}$(96.56%)d $^{0.02}$(0.05%)	15.49
(0.95084) BD (1) U1-As3<br>U1 (sd$^{2.98}$f $^{0.81}$)-As3 (sp$^{1.29}$)	(0.11587) LV (2)Al4<br>s(4.93%)p$^{19.26}$(94.96%)d $^{0.02}$(0.11%)	9.38

Beta MOs

Donor NBO	Acceptor NBO	E(2) kcal/mol
(0.91624) BD (1) U 1-As2<br>U1 (sd$^{2.59}$f $^{0.73}$)-As2 (sp$^{4.57}$)	(0.12946) LV (1)Al4<br>s(1.41%)p$^{70.05}$(98.49%)d$^{0.08}$(0.11%)	12.08
(0.91624) BD (1) 1-As2<br>U1 (sd$^{2.59}$f $^{0.73}$)-As2 (sp$^{4.57}$)	(0.11641) LV (2)Al4<br>s(6.51%)p$^{14.34}$(93.39%)d$^{0.02}$(0.10%)	20.92
(0.95093) BD (1) U1-As3<br>U1 (sd $^{2.94}$f $^{0.81}$)-As3 (sp$^{1.17}$)	(0.12946) LV (1)Al4<br>s(1.41%)p$^{70.05}$(98.49%)d $^{0.08}$(0.11%)	10.81
(0.95093) BD (1) U1-As3<br>U1 (sd$^{2.94}$f$^{0.81}$)-As3 (sp$^{1.17}$)	(0.11641) LV (2)Al4<br>s(6.51%)p$^{14.34}$(93.39%)d$^{0.02}$(0.10%)	14.02

Table S32: Computed Alpha Mos.

(201)HOMO (-0.15426 a.u.)	(200) (-0.16693 a.u.)
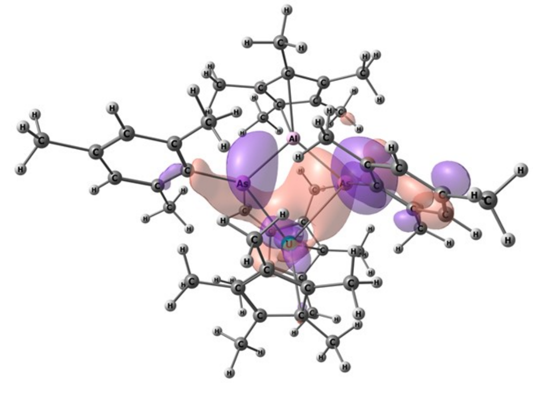	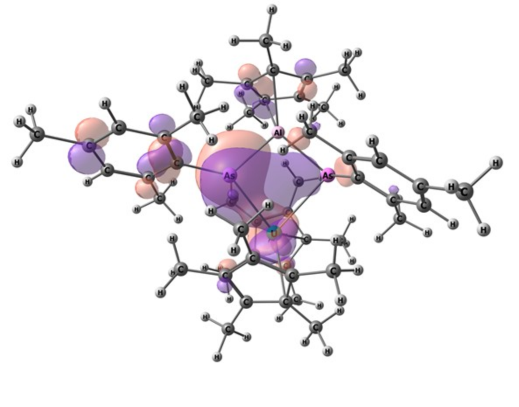
(199) (-0.17060 a.u.)	(193) (-0.21750 a.u.)

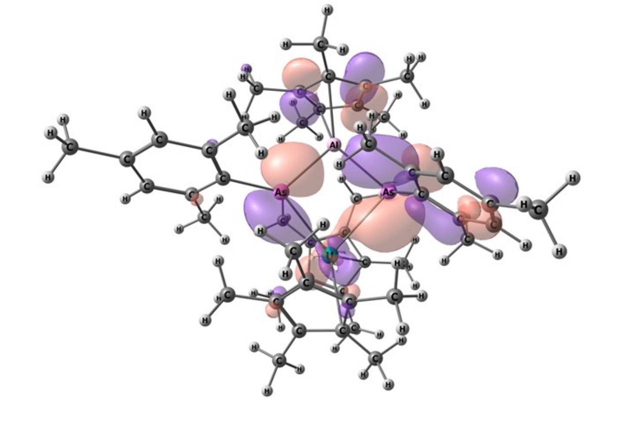	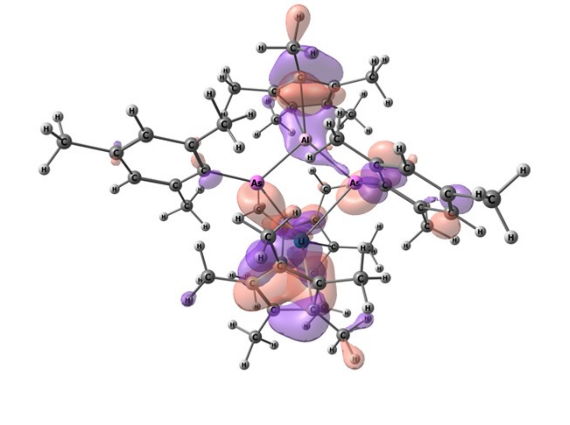
(189) (-0.22731 a.u.)	(187) (-0.23840 a.u.)
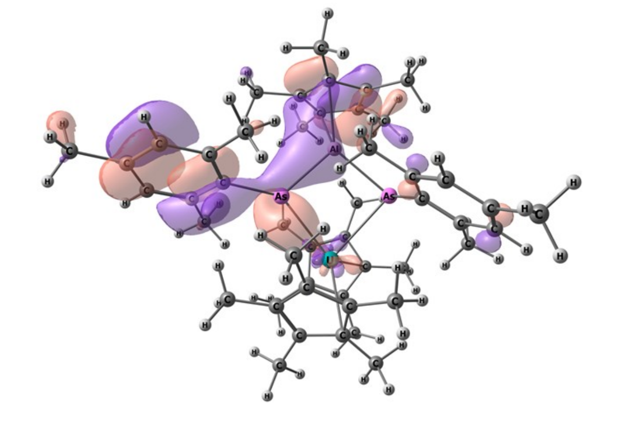	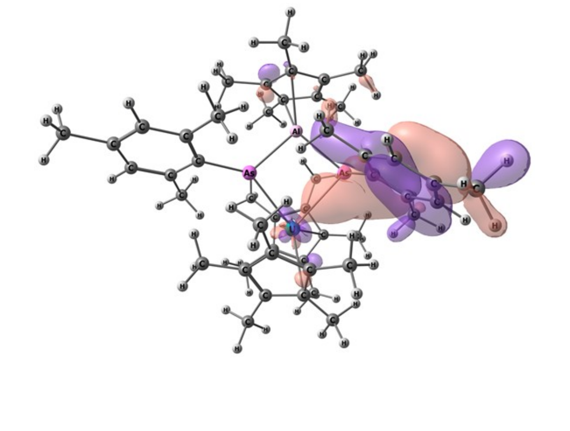
(186) (-0.24887 a.u.)	(103) (-0.52212 a.u.)
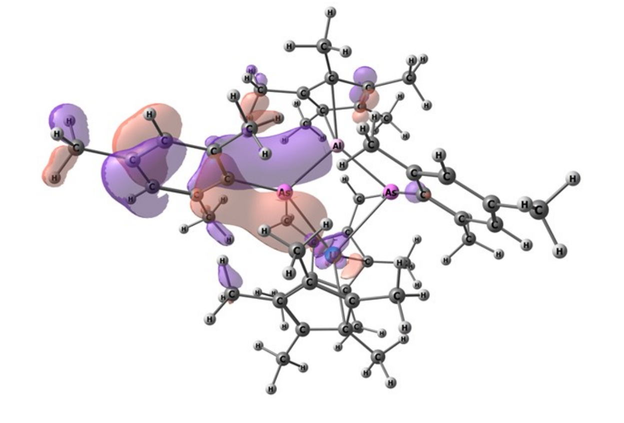	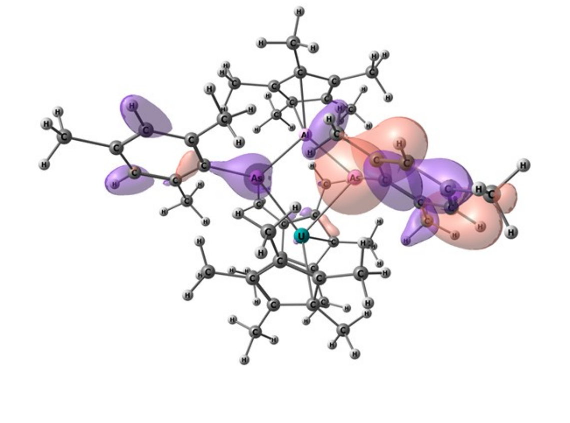
(102) (-0.53480 a.u.)	(98) (-0.58311 a.u.)
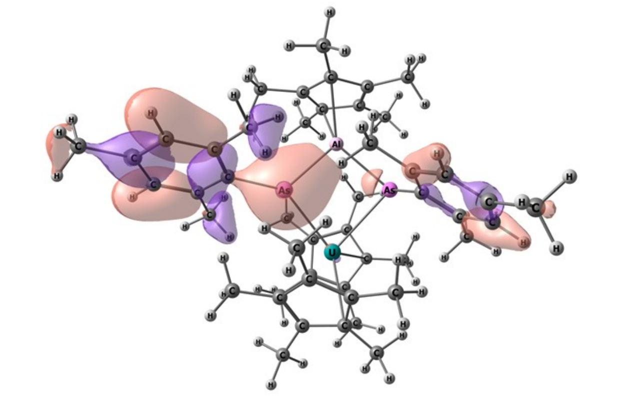	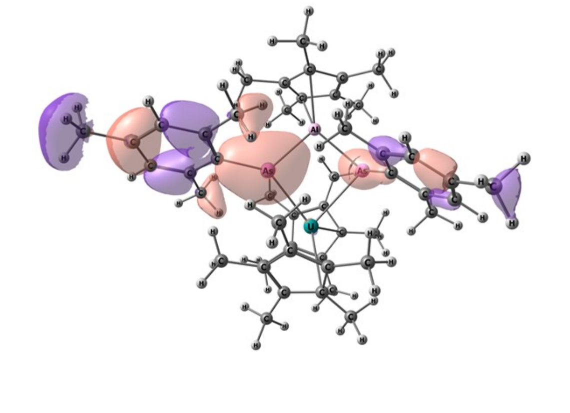

Table S33: Computed Beta Mos.

(199)HOMO (-0.15467 a.u.)<br>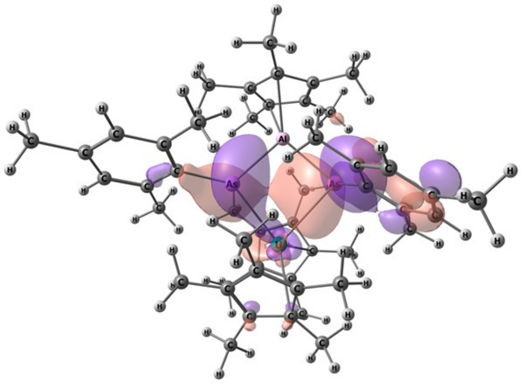	(198) (-0.16762 a.u.)<br>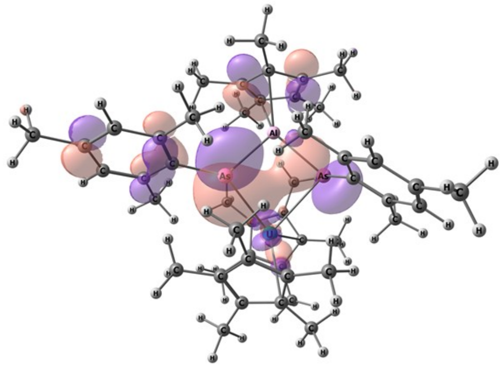
(197) (-0.17149 a.u.)<br>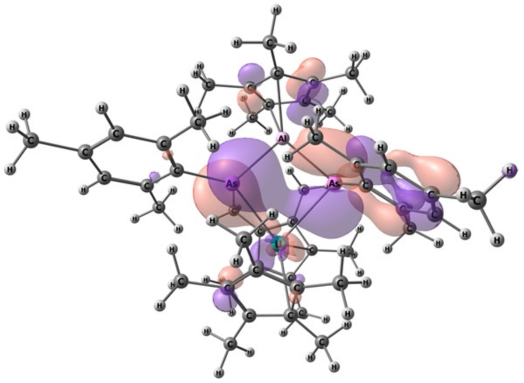	(193) (-0.21607 a.u.)<br>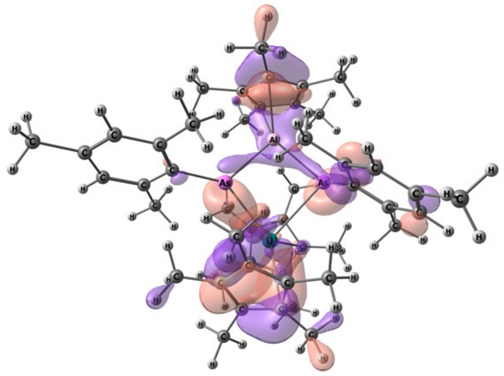
(189) (-0.22709 a.u.)<br>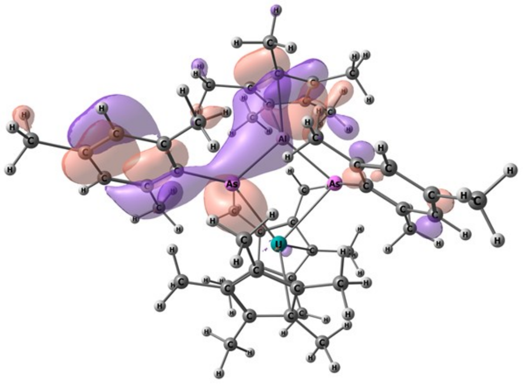	(187) (-0.23766 a.u.)<br>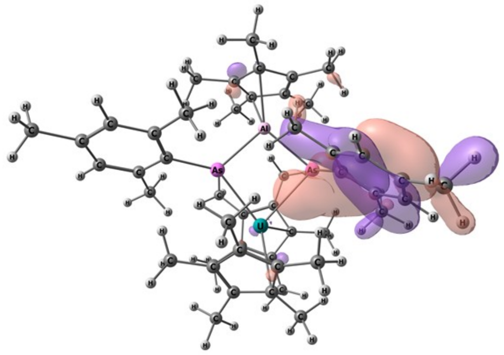
(186) (-0.24869 a.u.)<br>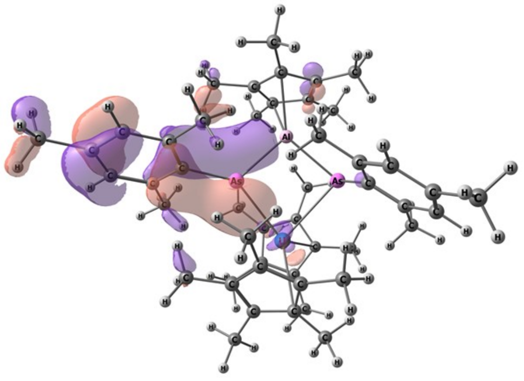	(103) (-0.52267 a.u.)<br>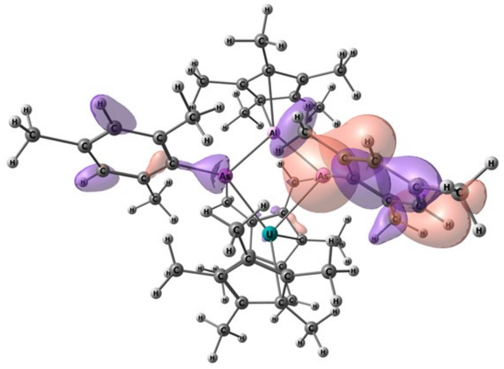

(102) (-0.53621 a.u.)	
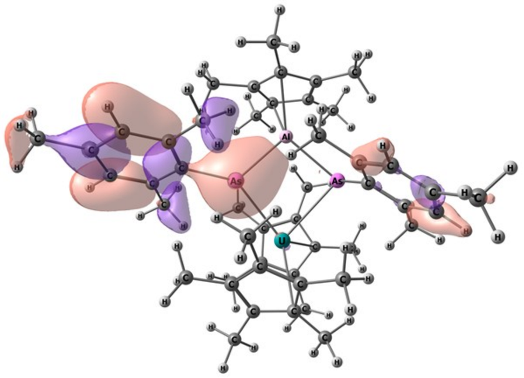	

Table S34: Computed BCP descriptors.

	ρ(r)	$^2\rho$(r)	G(r)	V(r)	H(r)	ε
U1-As2	0.049	0.022	0.018	-0.032	-0.013	0.102
U1-As3	0.060	0.065	0.032	-0.050	-0.017	0.465
As2-Al4	0.055	0.069	0.008	-0.037	-0.029	0.097
As3-Al4	0.053	0.100	0.010	-0.037	-0.026	0.073
U1-As2-As3-Al4	0.017	0.036	0.010	-0.010	-0.001	-1.491

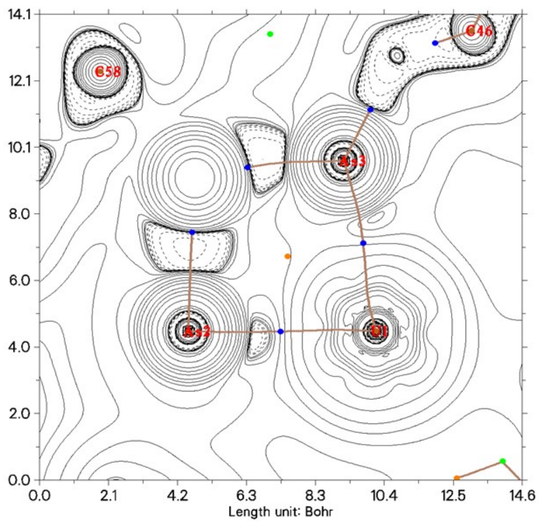

Compound 6

Table S35. Comparison of selected bond distances between DFT optimized structures and X-ray for U-N-Al

(S=3/2)	DFT		X-ray	
	dispersio n	No dispersion		
U1-N3	2,21421	2.22864	2.24403	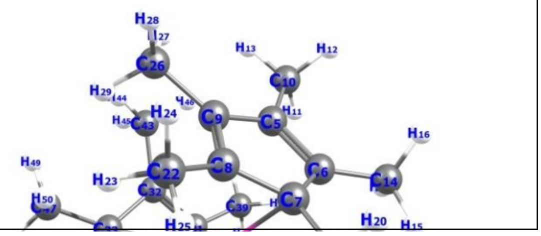

U1-N4	2,21523	2.22832	2.22497	
Al2-N3	1,85336	1.86772	1.83634	
Al2-N4	1,85371	1.86695	1.83332	
U1-X102	2,46011	2.51796	2.49980	
U1-X103	2,47920	2.54199	2.52652	

Table S36: Computed natural charges

Atom labels	Natural charges	Spin Density
U1	1.38873	2.13099
Al2	2.10583	0.01465
N3	-1.18229	-0.04023
N4	-1.18333	-0.04033

Table S37: Computed Wiberg bond index between selected atoms

Atom labels	Wiberg bond index
U1-N3	0.9399
U1-N4	0.9410
Al2-N3	0.3480
Al2-N4	0.3482

Alpha MOs

Table S38: NBO bond analysis

Bond	% Contribution	Orbitals			
		s	p	d	f
(0.96188) BD (1) U 1- N 3	(13.61%) 0.3690* U 1	9.23%	1.20%	53.00%	
	(86.39%) 0.9294* N 3	40.31%	59.68%	0.01%	-
(0.92491) BD (2) U 1- N 3	(13.31%) 0.3648* U 1	1.92%	0.15 %	42.55 %	55.37%
	(86.69%) 0.9311* N 3	16.26%	83.74%	0.01%	-
(0.87725) BD (3) U 1- N 3	(5.86%) 0.2421* U 1	0.59%	1.61%	48.37%	49.39%
	(94.14%) 0.9702* N 3	8.65%	91.34%	0.02%	-

(0.96151) BD (1) U 1- N 4	(13.61%) 0.3689* U 1	9.28%	1.22%	52.73%	36.74%
	(86.39%) 0.9295* N 4	40.47%	59.52%	0.01%	
(0.92604) BD (2) U 1- N 4	(13.49%) 0.3673* U 1	1.97%	0.13%	42.41%	55.47%
	(86.51%) 0.9301* N 4	42.41	84.03%	0.01%	
(0.87777) BD (3) U 1- N 4	(5.75%) 0.2398* U 1	0.58%	1.69%	48.69%	84.25%
	(94.25%) 0.9708* N 4	8.79	91.19%	0.02%	
(0.04129) BD*(1) U 1- N 3	(86.39%) 0.9294* U 1	9.23%	1.20%	53.00	36.54
	(13.61%) -0.3690* N 3	40.31%	59.68%	0.00%	
(0.03524) BD*(2) U 1- N 3	(86.69%) 0.9311* U 1	1.92%	0.15%	42.55%	28.87%
	(13.31%) -0.3648* N 3	16.26%	83.74%	0.00%	
(0.02781) BD*(3) U 1- N 3	(94.14%) 0.9702* U 1	0.59%)	1.61%)	48.37%)	49.39%
	(5.86%) -0.2421* N 3	8.65%	91.34%	0.02%	
(0.04079) BD*(1) U 1- N 4	(86.39%) 0.9295* U 1	9.28%	1.22%	52.73%	36.74%
	(13.61%) -0.3689* N 4	40.47%	59.52%	0.01%	
(0.03516) BD*(2) U 1- N 4	(86.51%) 0.9301* U 1	1.97%	0.13%	42.41%	55.47%
	(13.49%) -0.3673* N 4	15.97%	84.03%	0.01%	
(0.02787) BD*(3) U 1- N 4	(94.25%) 0.9708* U 1	0.58%	1.69%	48.69%	48.99%
	(5.75%) -0.2398* N 4	8.79%	91.19%	0.02%	

Table S39: NBO second order perturbation analysis

Donor NBO	Acceptor NBO	E(2) kcal/mol
(0.87777) BD (3) U 1- N 4<br>U 1 ($sp^{2.91}d^{83.73}f^{84.25}$)-N4 ($sp^{10.37}$)	(0.47895) LV (1) C 91<br>s(0.13%)$p^{99.99}$(99.87%)d 0.06(0.01%)	29.27
(0.98533) CR (2) U 1<br>s(100.00%)	(0.08216) LV (3)Al 2<br>s(1.69%)$p^{58.19}$(98.15%)$d^{0.10}$(0.16%)	26.89
(0.96188) BD (1) U 1- N 3<br>U 1 ($sp^{0.13}d^{5.75}$)-N3 ($sp^{1.48}$)	(0.08216) LV (3)Al 2<br>s(1.69%)$p^{58.19}$(98.15%)$d^{0.10}$(0.16%)	14.24
(0.92491) BD (2) U 1- N 3<br>U1 ($sp^{0.08}d^{22.19}f^{28.87}$)-N3 ($sp^{5.15}$)	(0.18173) LV (1)Al 2<br>s(97.38%)	20.44
(0.96151) BD (1) U 1- N 4<br>U1 ($sp^{0.13}d^{5.68}f^{3.96}$)	(0.08216) LV (3)Al 2<br>s(1.69%)$p^{58.19}$(98.15%)$d^{0.10}$(0.16%)	14.62
(0.87725) BD (3) U 1- N 3<br>U 1 ($sp^{2.71}d^{81.47}f^{83.19}$)-N 3 ($sp^{10.56}$)	(0.18173) LV (1)Al 2<br>s(97.38%)$p^{0.02}$(2.42%)	28.41
(0.87777) BD (3) U 1- N 4<br>U 1 ($sp^{2.91}d^{83.73}f^{84.25}$)-N 4 ($sp^{10.37}$)	(0.18173) LV (1)Al 2<br>s(97.38%)$p^{0.02}$(2.42%)d 0.00(0.21%)	29.12
(0.92604) BD (2) U 1- N 4<br>U 1 ($sp^{0.07}d^{21.55}f^{28.19}$) 55.47%)-*N4<br>($sp^{5.26}$)	(0.18173) LV (1)Al 2<br>s(97.38%)$p^{0.02}$(2.42%)	19.60

Table S40: Computed Alpha MOs.

(177)homo-2 (-0.18569 a.u.)	(169) (-0.23725 a.u.)

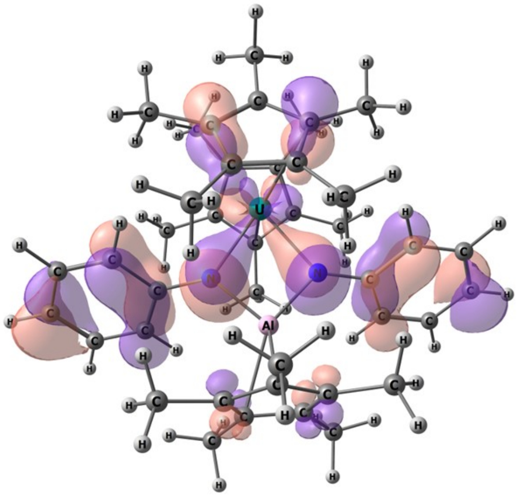	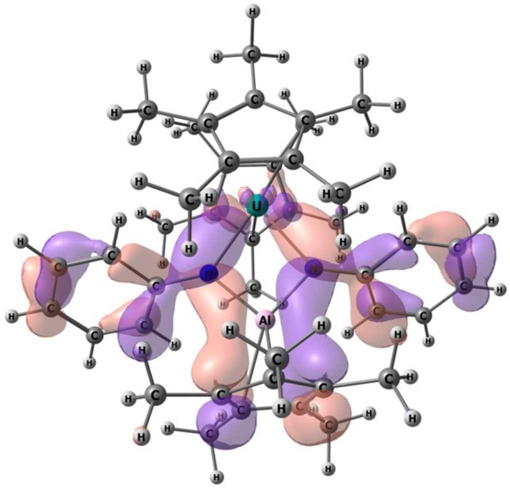
(166) (-0.25715 a.u.)<br>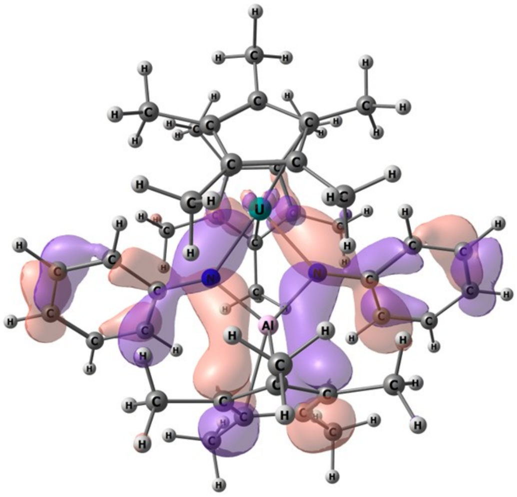	(165) (-0.29837 a.u.)<br>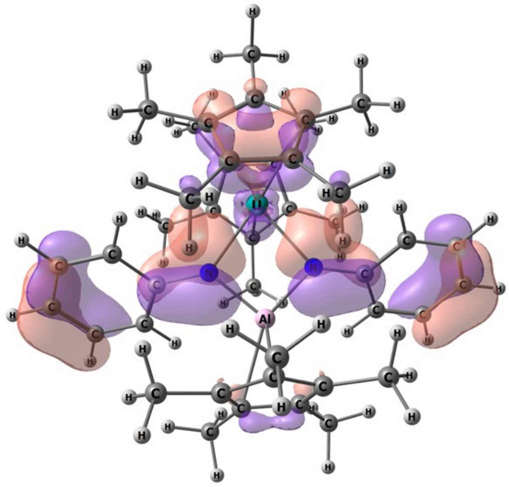
(164) (-0.30567 a.u)<br>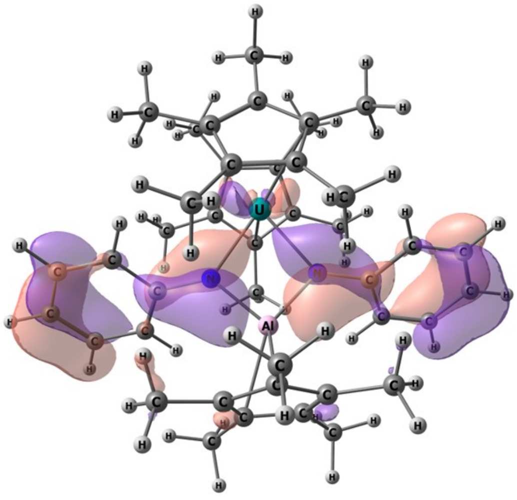	

Beta MOs

Table S41: NBO bond analysis

Bond	% Contribution	Orbitals			
		s	p	d	f
(0.96092) BD (1) U 1- N 3	(12.12%) 0.3481* U 1	10.24%	1.44%	56.15%	
	(87.88%) 0.9375* N 3	41.92%	58.07%	0.01%	-
(0.92590) BD (2) U 1- N 3	(10.81%) 0.3287* U 1	2.27%	0.18 %	42.54%	55.00%
	(89.19%) 0.9444* N 3	17.77%	82.22%	0.01%	-
(0.87914) BD (3) U 1- N 3	(5.59%) 0.2363* U 1	036%	2.05%	466.86 %	50.68%
	(94.41%) 0.9717* N 3	6.16%	93.82%	0.01%	-
(0.96053) BD (1) U 1- N 4	(12.11%) 0.3481* U 1	10.29%	1.47%	55.89%	32.31%
	(87.89%) 0.9375* N 4	42.08%	57.91%	0.01%	
(0.92689) BD (2) U 1- N 4	(10.95%) 0.3309* U 1	2.33%	0.16%	42.61%	54.89%
	(89.05%) 0.9437* N 4	17.50	82.49%	0.01%	
(0.87966) BD (3) U 1- N 4	(5.50%) 0.2345* U 1	0.35%	2.15%	47.15%	50.30%
	(94.50%) 0.9721* N 4	6.30%	93.68%	0.02%	
(0.03883) BD*(1) U 1- N 3	(87.88%) 0.9375* U 1	10.24%	1.44%	56.15%	32.12%
	(12.12%) -0.3481* N 3	41.92%	58.07%	0.01%	
(0.02890) BD*(2) U 1- N 3	(89.19%) 0.9444* U 1	2.27%	0.18%	42.54%	55.00%
	(10.81%) -0.3287* N 3	17.77%	82.22%	0.01%	
(0.02297) BD*(3) U 1- N 3	(94.41%) 0.9717* U 1	0.36%	2.05%	46.86%	50.68%
	(5.59%) -0.2363* N 3	6.16%	93.82%	0.01%	
(0.03850) BD*(1) U 1- N 4	(87.89%) 0.9375* U 1	10.29%	1.47%	55.89%	32.31%
	(12.11%) -0.3481* N 4	42.08%	57.91%	0.01%	
(0.02897) BD*(2) U 1- N 4	(89.05%) 0.9437* U 1	2.33%	0.16%	42.61%	54.89%
	(10.95%) -0.3309* N 4	17.50%	82.49%	0.01%	
(0.02318) BD*(3) U 1- N 4	(94.50%) 0.9721* U 1	0.35%	2.15%	47.15%	50.30 %
	(5.50%) -0.2345* N 4	6.30%	93.68%	0.01%	

Table S42: NBO second-order perturbation analysis

Donor NBO	Acceptor NBO	E(2) kcal/mol
(0.87914) BD (3) U 1- N 3<br>U 1 ($sp^{5.67}d^{99.99}f^{99.99}$)-*N3 ($sp^{15.23}$)	(0.47432) LV (1) C 80<br>s(0.18%)$p^{99.99}$(99.81%)	34.05
(0.87966) BD (3) U 1- N 4<br>U 1 $sp^{6.14}d^{99.99}f^{99.9}$)-* N 4 ($sp^{14.87}$)	(0.47480) LV (1) C 91<br>s(0.19%)$p^{99.99}$(99.80%)	33.53
(0.98469) CR (2) U 1<br>s(100.00%)	(0.08117) LV (3)Al 2<br>s(0.95%)$p^{99.99}$(98.91%)$d^{.15}$(0.14%)	28.25
(0.96092) BD (1) U 1- N 3<br>* U 1 ($sp^{0.14}d^{5.48}$(56.15%)$f^{3.14}$)-* N 3($sp^{1.39}$)	(0.08117) LV (3)Al 2<br>s(0.95%)$p^{99.99}$(98.91%)$d^{0.15}$(0.14%)	15.06
(0.92590) BD (2) U 1- N 3<br>* U 1 ($sp^{0.08}d^{18.78}f^{24.28}$)-* N 3 ($sp^{4.63}$)	(0.16970) LV (1)Al 2<br>s(97.40%) $^{0.02}$(2.35%)	20.58
(0.87914) BD (3) U 1- N 3<br>* U 1 ($sp^{5.67}d^{99.99}f^{99.99}$)-* N 3 ($sp^{15.23}$)	(0.16970) LV (1)Al 2<br>s(97.40%)$p^{0.02}$(2.35%)d	23.48
(0.96053) BD (1) U 1- N 4<br>* U 1 ($sp^{0.14}d^{5.43}f^{3.14}$)-* N 4 ($sp^{1.38}$)	(0.08117) LV (3)Al 2<br>s(0.95%)$p^{99.99}$(98.91%)$d^{0.15}$(0.14%)	15.51
77. (0.92689) BD (2) U 1- N 4<br>* U 1 ($sp^{0.07}d^{18.32}f^{23.60}$)-* N 4 ($sp^{4.71}$)	185. (0.16970) LV (1)Al 2<br>s(97.40%) $^{0.02}$(2.35%)	19.80
(0.87966) BD (3) U 1- N 4	(0.16970) LV (1)Al 2	24.26

* U 1 ($sp^{6.14}d^{99.99}f^{99.99}$)-* N 4 ($sp^{14.87}$)	s(97.40%)$p^{0.02}$(2.35%)	

Table S43: Computed Beta Mos.

(177)HOMO (-0.18519 a.u.) 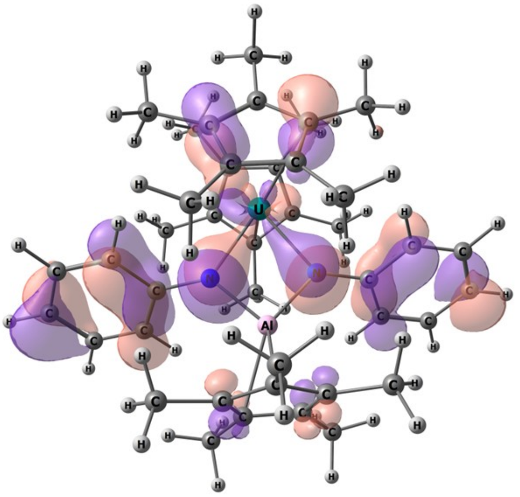	(176) (-0.18721 a.u.) 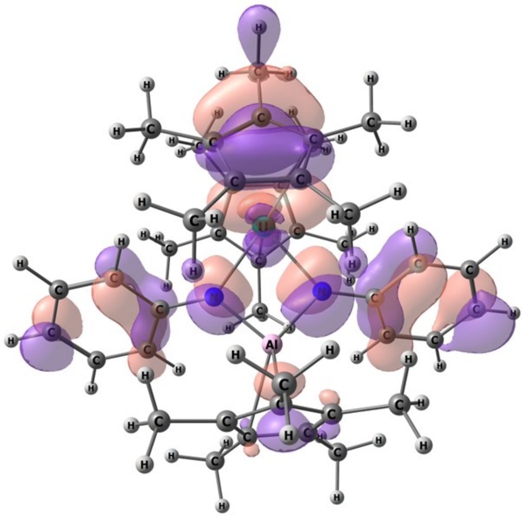
(171) (-0.21813 a.u.) 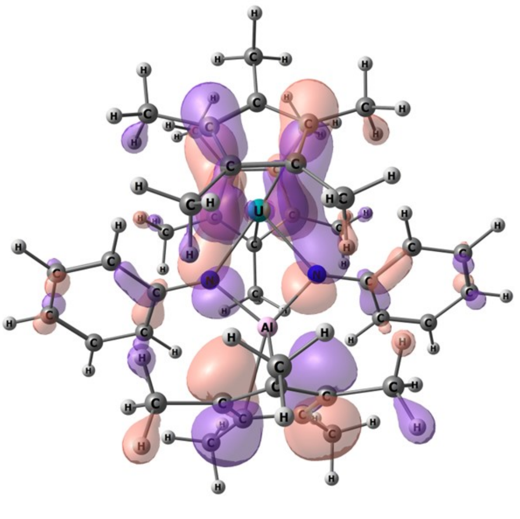	(169) (-0.23736 a.u.) 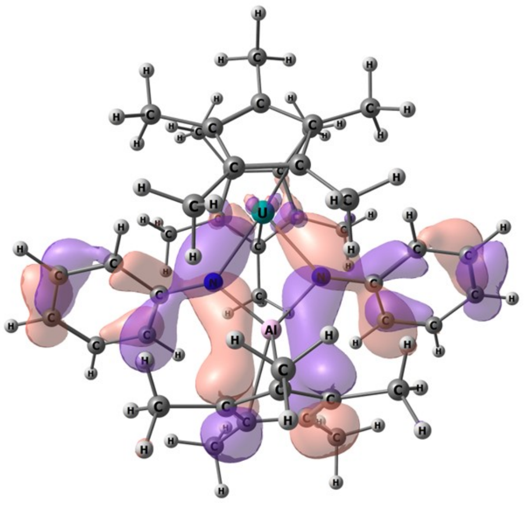
(166) (-0.25673 a.u.)	(165) (-0.29745 a.u.)

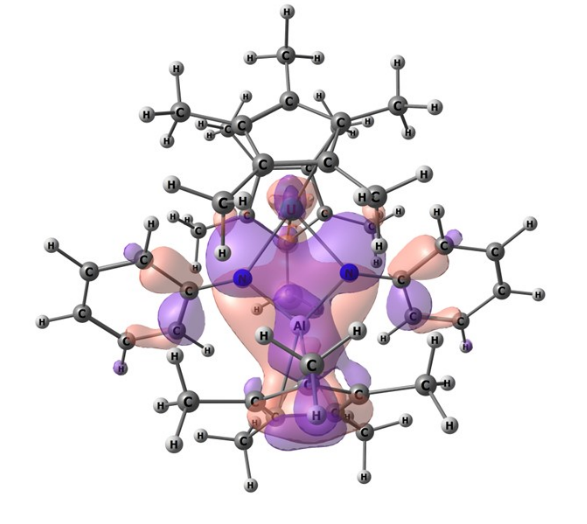

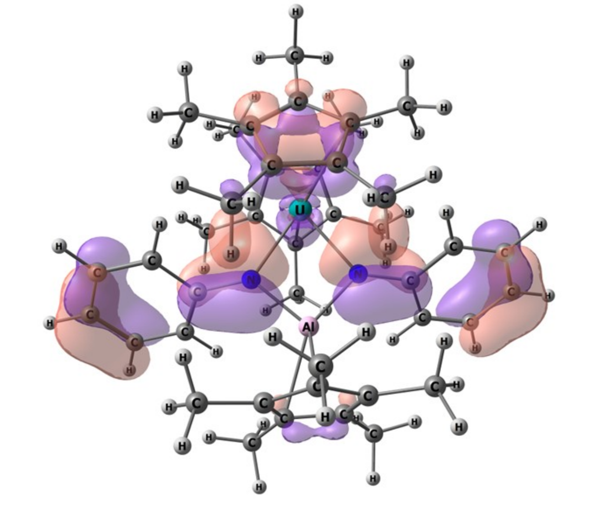

(164) (-0.30555 a.u)

(58) (-0.88995 a.u.)

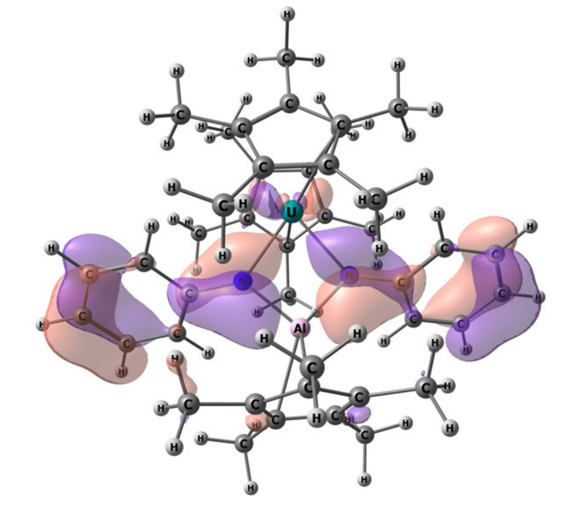

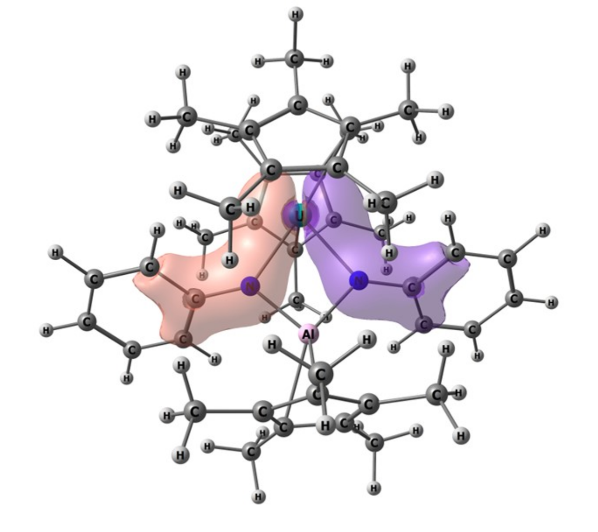

(55) (-0.91097 a.u.)

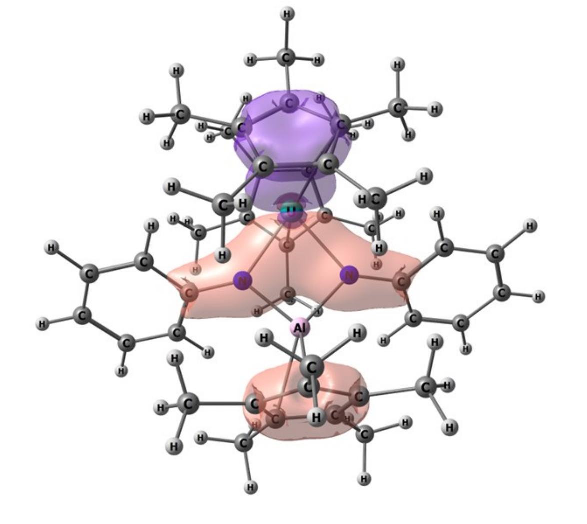

Table S44: Computed BCP descriptors

	ρ(r)	$^2\rho$(r)	G(r)	V(r)	H(r)	ε
U1-N3	0.111	0.262	0.102	-0.137	-0.036	0.110
U1-N4	0.111	0.262	0.101	-0.137	-0.036	0.111
Al2-N3	0.079	0.522	0.037	-0.061	-0.024	0.098
Al2-N4	0.079	0.522	0.037	-0.061	-0.024	0.100
U1-N3-Al2-N4	0.032	0.120	0.030	-0.031	-0.001	- 1.540

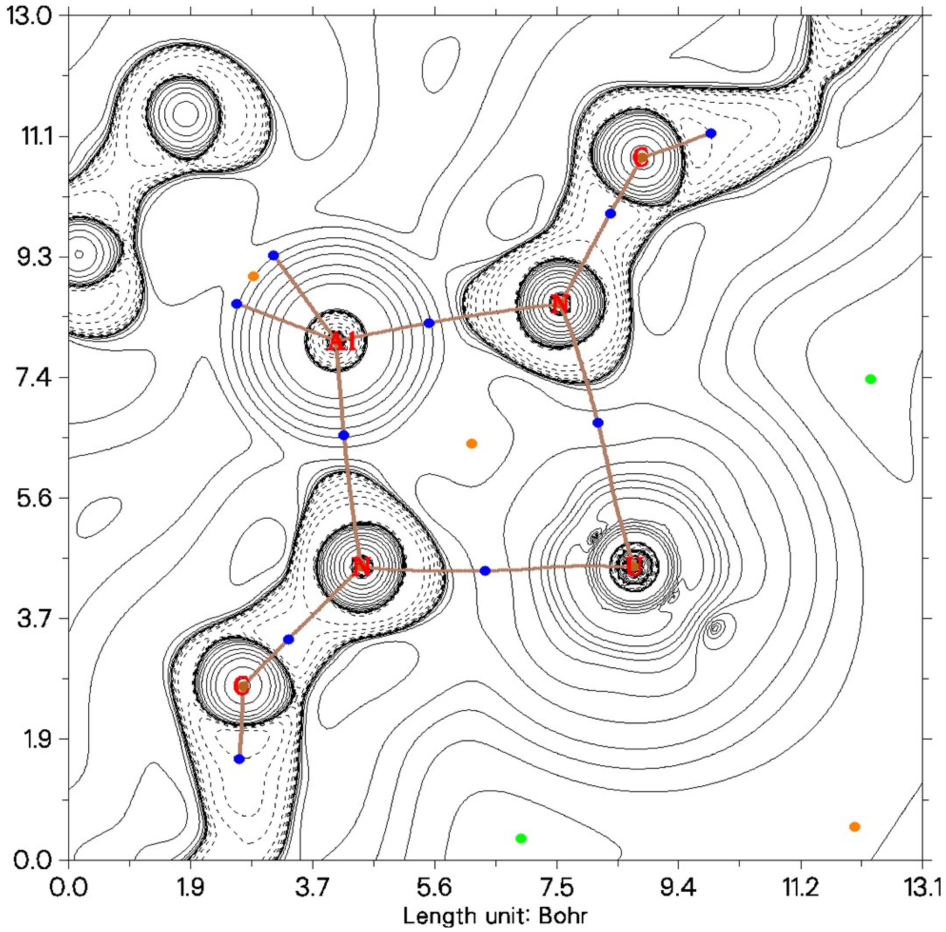

Compound 5

Table S45. Comparison of selected bond distances between DFT optimized structures and X-ray for Th-N-Al

(S=1/2)	DFT		X-ray
	dispersion	No dispersion	
Th 1-N3	2.27897	2.29091	2.30167
Th 1-N4	2.28014	2.29388	2.28073
Al2-N3	1.84858	1.86523	1.81634
Al2-N4	1.84902	1.86485	1.82149
Th1-X102	2.53031	2.58982	2.55080
Th1-X103	2.55114	2.62137	2.57802

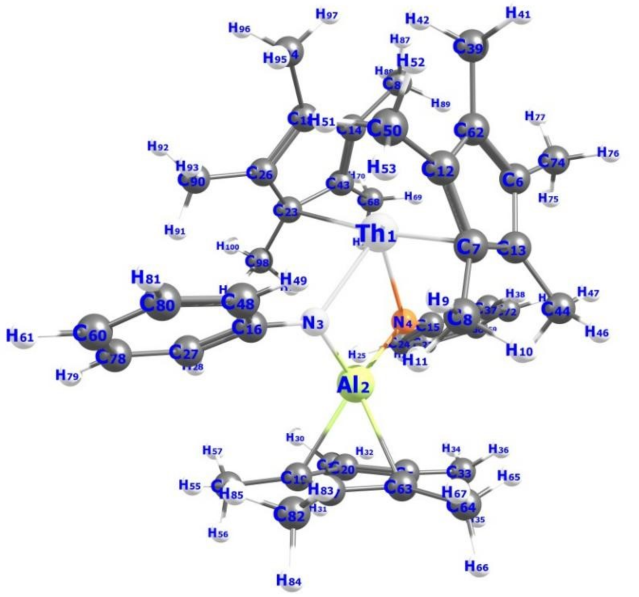

Table S46: Computed natural charges

Atom labels	Natural charges
Th1	1.62916
N3	-1.27913
N4	-1.28040
Al2	2.13693

Table S47: Computed Wiberg bond index between selected atoms

Atom labels	Wiberg bond index
Th1-N3	0.7728
Th1-N4	0.7733
Al2-N3	0.3477
Al2-N4	0.3477

Table S48: NBO bond analysis

Bond	% Contribution	Orbitals			
		s	p	d	f
(1.92184) BD (1)Th 1- N 3	(9.34%) 0.3056*Th 1	8.61%	1.58%	68.22%	21.48 %
	(90.66%) 0.9522* N 3	38.62 %	61.36%	0.01%	-
(1.92108) BD (1)Th 1- N 4	(9.33%) 0.3055*Th 1	8.64%	1.61%	67.98%	21.67 %
	(90.67%) 0.9522* N 4	38.89 %	61.10%	0.01%	-
(0.08703) BD*(1)Th 1- N 3	(90.66%) 0.9522*Th 1	8.61%	1.58%	68.22%	21.48 %
	(9.34%) -0.3056* N 3	38.62 %	61.36%	0.01%	-
(0.08792) BD*(1)Th 1- N 4	(90.67%) 0.9522*Th 1	8.64%	1.61%	67.98%	21.67
	(9.33%) -0.3055* N 4	38.89 %	61.10%	0.01%	

Table S49: NBO second-order perturbation analysis

Donor NBO	Acceptor NBO	E(2) kcal/mol
(1.99980) CR (1)Th 1 s(100.00%)	(0.17655) LV (2)Al 2 s(12.98%)p$^{6.68}$(86.73%)	12.45
(1.97143) CR (2)Th 1 s(100.00%)	(0.17655) LV (2)Al 2 s(12.98%)p$^{6.68}$(86.73%)	38.01
(1.92184) BD (1)Th 1- N 3 Th1 (sp$^{0.18}$d $^{7.92}$f$^{2.49}$)-N3 (sp$^{1.59}$)	(0.20402) LV (1)Al 2 s(2.57%)$^{37.96}$(97.40%)	17.69
(1.92184) BD (1)Th 1- N 3 Th1 (sp$^{0.18}$d$^{7.92}$f$^{2.49}$)-*N3 (sp$^{1.59}$)	(0.17655) LV (2)Al 2 s(12.98%)p$^{6.68}$(86.73%)	14.33
(1.92108) BD (1)Th 1- N 4 Th1 (sp$^{0.19}$d$^{7.87}$f^{2}·51)-N4 (sp$^{1.57}$)	(0.20402) LV (1)Al 2 s(2.57%)p$^{37.96}$(97.40%)	18.17
(1.92108) BD (1)Th 1- N 4 Th1 (sp$^{0.19}$d$^{7.87}$f$^{2.51}$)-*N4 (sp$^{1.57}$)	186. (0.17655) LV (2)Al 2 s(12.98%)p$^{6.68}$(86.73%)	14.07

Table S50: Computed Alpha Mos.

(177)HOMO (-0.18157 a.u.)	(176) (-0.18512 a.u.)
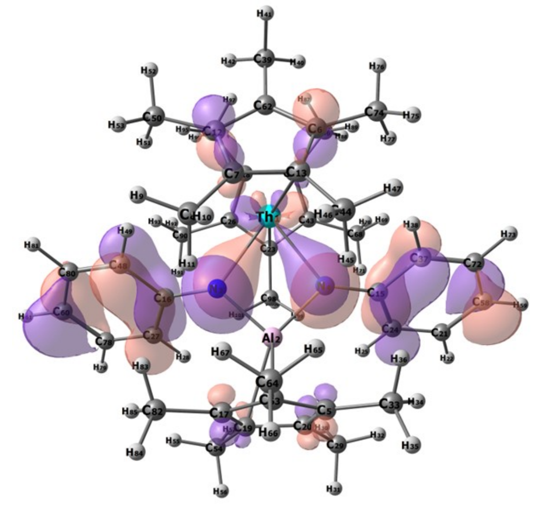	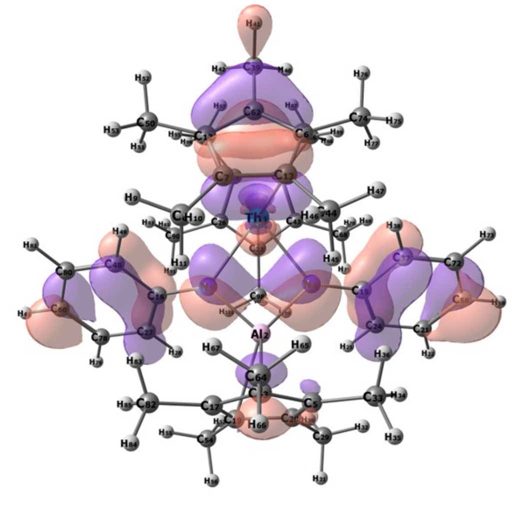
(172) (-0.20993 a.u.)	(169) (-0.23685 a.u.)
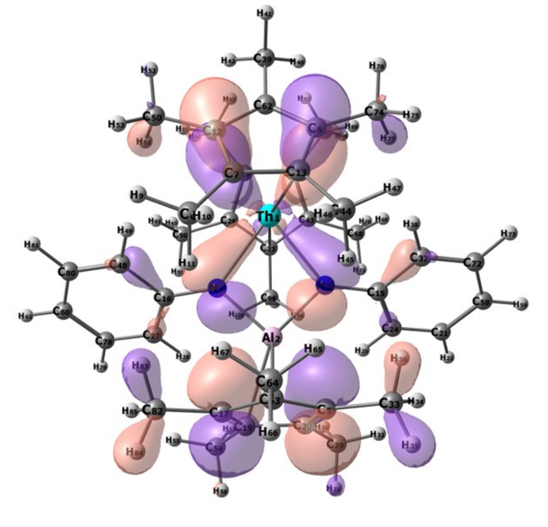	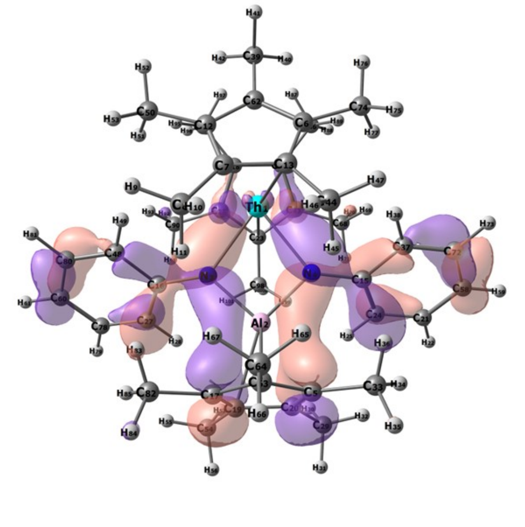
(166) (-0.25389 a.u.)	(165) (-0.29535 a.u.)
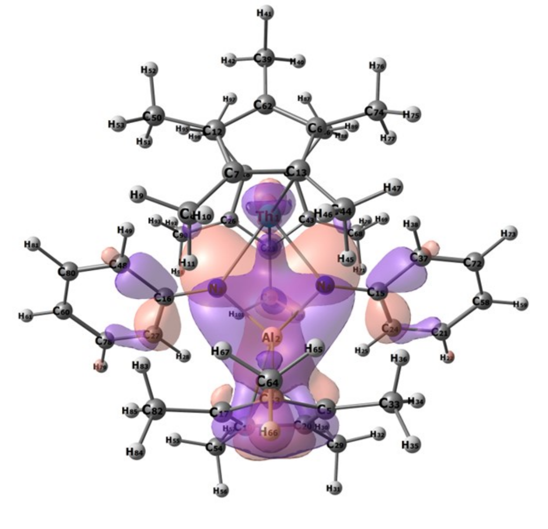	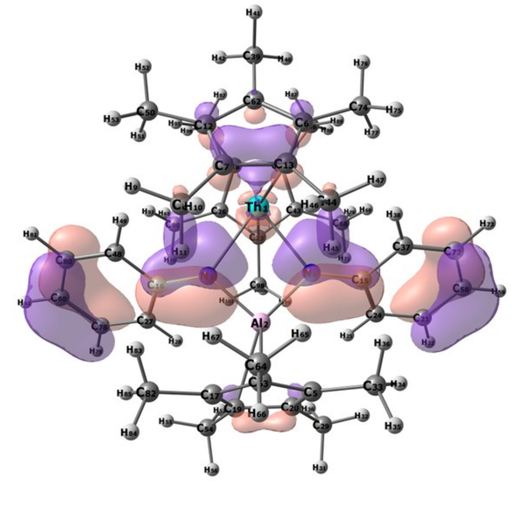
(164) (-0.30275 a.u.)	(146) (-0.36023 a.u.)

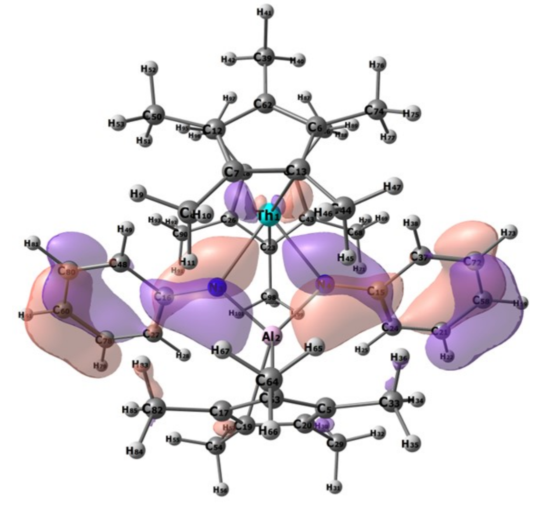

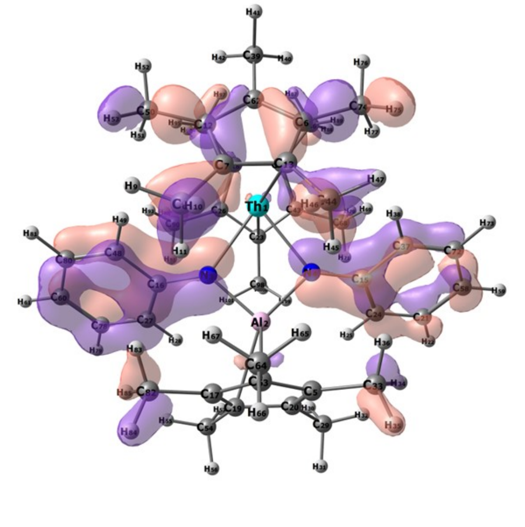

(145) (-0.36130 a.u.)

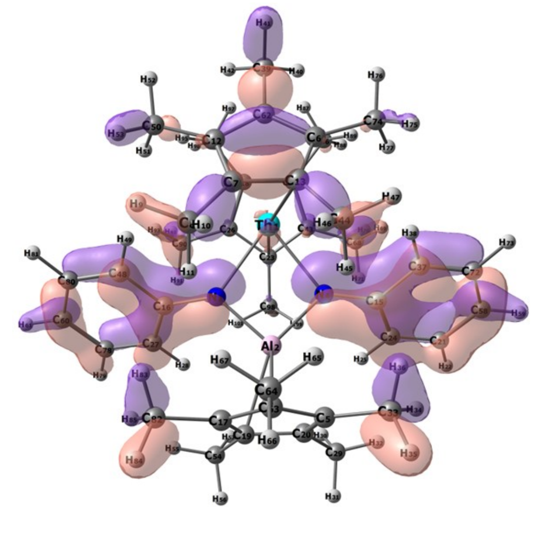

(83) (-0.68217 a.u.)

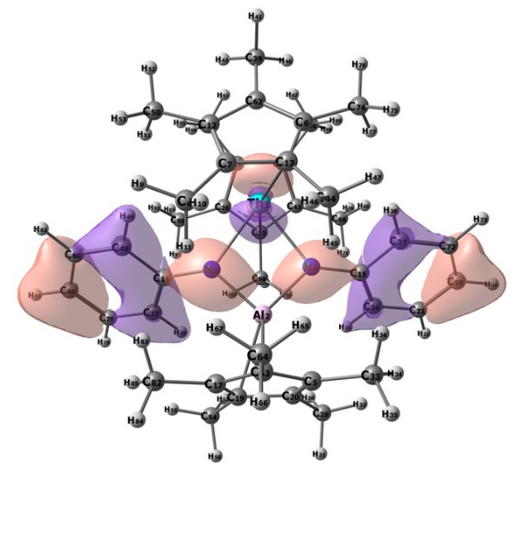

(58) (-0.87316 a.u.)

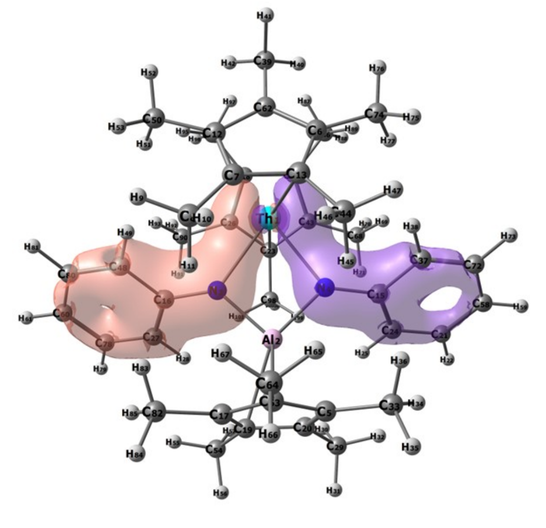

Table S51: Computed BCP descriptors

	ρ(r)	$^2\rho$(r)	G(r)	V(r)	H(r)	ε
Th1-N3	0.101	0.211	0.085	-0.117	-0.322	0.222
Th1-N4	0.101	0.212	0.085	-0.117	-0.032	0.223
Al2-N3	0.079	0.531	0.038	-0.062	-0.024	0.095
Al2-N4	0.079	0.530	0.038	-0.062	-0.024	0.097
Th1-N3- Al2-N4	0.301	0.117	0.029	-0.293	-0.00001	-1.445

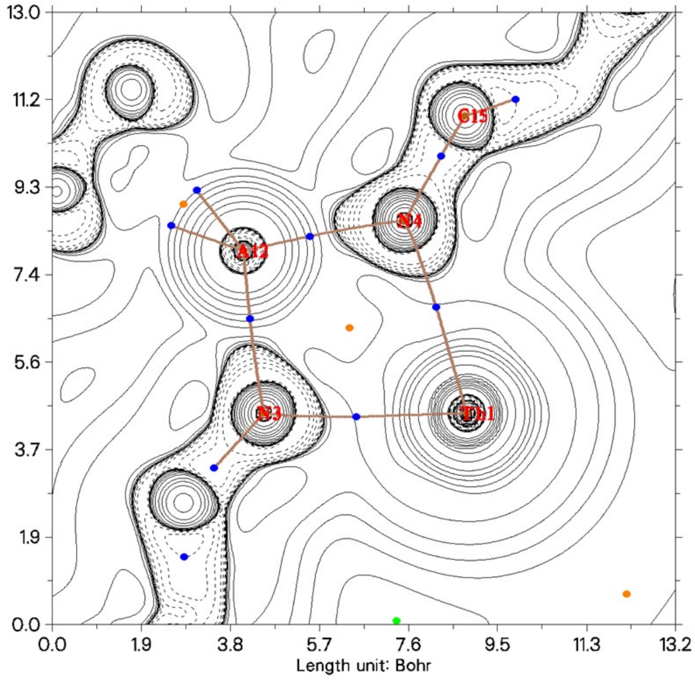

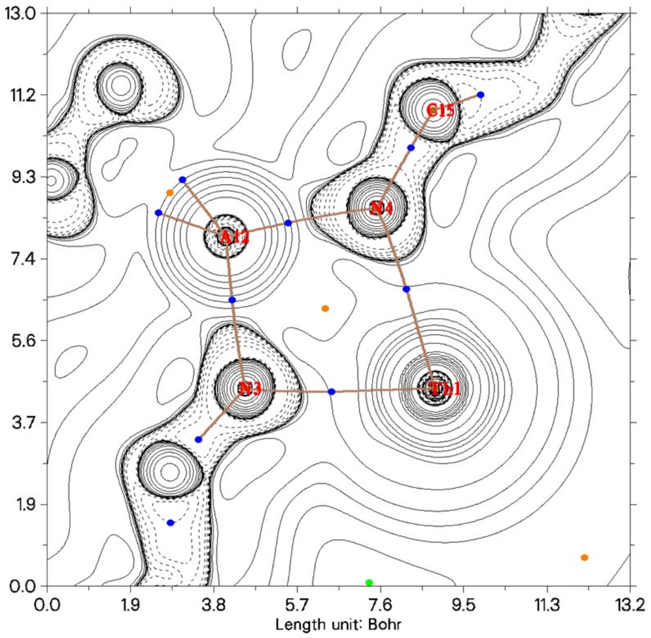

Cordinates

Compound **1** (Th-P-Al)

Multiplicity = 1

Th	-1.735524000	8.446450000	11.320903000
P	0.249797000	9.849622000	12.652293000
P	-1.325880000	10.712631000	9.590004000
Al	0.077617000	11.683054000	11.261661000
C	-3.787793000	9.972282000	12.415640000
C	-3.837229000	8.735097000	13.113735000
C	-4.340068000	7.746797000	12.220702000
C	-4.568480000	8.369650000	10.964911000
C	-4.250072000	9.750540000	11.091845000
C	-3.408500000	11.301987000	12.978627000
H	-2.796124000	11.195382000	13.874547000
H	-4.296277000	11.894466000	13.236126000
H	-2.825962000	11.881393000	12.257830000
C	-3.634493000	8.526483000	14.581860000
H	-3.055906000	7.623772000	14.805857000
H	-4.601039000	8.415067000	15.090006000
H	-3.118074000	9.370729000	15.038665000
C	-4.835523000	6.404806000	12.657822000
H	-4.967333000	5.716671000	11.822782000
H	-5.813191000	6.515400000	13.145356000
H	-4.171935000	5.932305000	13.384116000
C	-5.188698000	7.745579000	9.750813000
H	-4.489806000	7.643508000	8.910363000
H	-6.022355000	8.355251000	9.386342000
H	-5.581165000	6.749961000	9.969761000
C	-4.616721000	10.813294000	10.106994000
H	-3.961186000	11.681424000	10.183762000
H	-5.649752000	11.142483000	10.286567000
H	-4.552070000	10.456553000	9.078034000
C	-1.705323000	5.734368000	10.651963000
C	-1.124674000	5.773091000	11.949361000
C	0.134387000	6.424297000	11.852957000
C	0.348664000	6.764822000	10.489341000
C	-0.795953000	6.365692000	9.752473000
C	-2.898830000	4.948383000	10.205058000
H	-3.421730000	4.494738000	11.047925000
H	-2.581973000	4.134142000	9.541862000
H	-3.622775000	5.546840000	9.645275000
C	-1.623111000	5.124506000	13.203299000
H	-1.802326000	5.840890000	14.015314000
H	-0.880792000	4.411953000	13.581116000
H	-2.550200000	4.574404000	13.035222000
C	1.121091000	6.546538000	12.971510000
H	1.884439000	7.293887000	12.752192000
H	1.625380000	5.587505000	13.151490000
H	0.648632000	6.849543000	13.910345000
C	1.623679000	7.278590000	9.911057000
H	1.460621000	7.834413000	8.986212000
H	2.296264000	6.442534000	9.679391000
H	2.139016000	7.932620000	10.615281000
C	-0.967034000	6.435739000	8.266891000
H	-1.995276000	6.680362000	7.979175000
H	-0.730901000	5.468733000	7.803510000
H	-0.318163000	7.187461000	7.815678000
C	1.540638000	13.435805000	11.134701000
C	1.155356000	13.346427000	12.508941000
C	-0.244200000	13.565052000	12.592680000
C	-0.736663000	13.814906000	11.271575000
C	0.364698000	13.743811000	10.370138000
C	2.953495000	13.467461000	10.640033000
H	3.579155000	12.710693000	11.120681000
H	3.403134000	14.444726000	10.852935000
H	3.016328000	13.311345000	9.560749000
C	2.057206000	13.142874000	13.681760000
H	1.611987000	12.475270000	14.423278000
H	2.265955000	14.100477000	14.176313000
H	3.015582000	12.708782000	13.390750000
C	-1.007830000	13.546514000	13.876684000

H	-2.075306000	13.711694000	13.720185000
H	-0.646014000	14.326696000	14.555969000
H	-0.885349000	12.583538000	14.386016000
C	-2.133952000	14.143842000	10.852604000
H	-2.472446000	13.464678000	10.061547000
H	-2.189672000	15.165927000	10.459707000
H	-2.836640000	14.073916000	11.685862000
C	0.262665000	13.951271000	8.893974000
H	1.207660000	13.741560000	8.386993000
H	-0.013462000	14.986536000	8.660980000
H	-0.500457000	13.293259000	8.461144000
C	1.428625000	9.801727000	14.061494000
C	2.819186000	9.892445000	13.824831000
C	3.704715000	9.861959000	14.902349000
H	4.772239000	9.939695000	14.704761000
C	3.264892000	9.733261000	16.220018000
C	1.892487000	9.617972000	16.437913000
H	1.523086000	9.502713000	17.455217000
C	0.973587000	9.645896000	15.387713000
C	3.370626000	10.025544000	12.436352000
H	2.883679000	10.841073000	11.894374000
H	3.186838000	9.121783000	11.845987000
H	4.449056000	10.206811000	12.455011000
C	4.236595000	9.743342000	17.367573000
H	4.424286000	10.766072000	17.717648000
H	5.201905000	9.317244000	17.078132000
H	3.855222000	9.173076000	18.219767000
C	-0.487753000	9.501862000	15.684765000
H	-0.898852000	8.612161000	15.197260000
H	-1.051946000	10.351531000	15.285769000
H	-0.673665000	9.424511000	16.759647000
C	-0.388907000	10.065217000	8.123714000
C	-1.166451000	9.599272000	7.032960000
C	-0.543810000	9.115674000	5.881818000
H	-1.166022000	8.755514000	5.064394000
C	0.844005000	9.069438000	5.752168000
C	1.603186000	9.536658000	6.824541000
H	2.689522000	9.510958000	6.757819000
C	1.017171000	10.033114000	7.990874000
C	-2.665650000	9.600191000	7.097040000
H	-3.030693000	8.980306000	7.925241000
H	-3.102023000	9.220256000	6.168861000
H	-3.050145000	10.608240000	7.288178000
C	1.496580000	8.562826000	4.495784000
H	1.667338000	9.376138000	3.779251000
H	0.873944000	7.815080000	3.995275000
H	2.469051000	8.107251000	4.706561000
C	1.904743000	10.527519000	9.088174000
H	1.629011000	10.074657000	10.048951000
H	1.812680000	11.612890000	9.188047000
H	2.957745000	10.301997000	8.897563000

Compound **2** (Th-As-Al)

Multiplicity = 1

Th	10.732367000	9.374492000	3.818260000
As	10.365243000	11.742041000	2.035484000
As	8.693174000	10.802359000	5.216951000
Al	8.999464000	12.758812000	3.867296000
C	8.561971000	7.769007000	3.054091000
C	12.789192000	10.910238000	4.898368000
C	9.233564000	11.081952000	0.568738000
C	12.834460000	9.667559000	5.587077000
C	7.823162000	11.079293000	0.536612000
C	7.555769000	10.868072000	6.797136000
C	7.989272000	7.416450000	5.573816000
H	8.549427000	7.596442000	6.496525000
H	7.441390000	6.473356000	5.703581000
H	7.258495000	8.222175000	5.483642000
C	7.243231000	10.995170000	9.198908000
H	7.674907000	11.019072000	10.197874000
C	7.997536000	14.421863000	5.153068000

C	7.150861000	10.578809000	-0.581421000
H	6.062152000	10.571190000	-0.575073000
C	8.883627000	7.348516000	4.374776000
C	13.544718000	9.312693000	3.428730000
C	8.095781000	10.881199000	8.098798000
C	7.016033000	11.614602000	1.677767000
H	7.330149000	11.169178000	2.629872000
H	5.947629000	11.420801000	1.547175000
H	7.149407000	12.697044000	1.757661000
C	9.929159000	10.598569000	-0.565651000
C	10.137832000	6.681432000	4.329849000
C	9.399678000	14.642601000	5.165951000
C	9.823971000	14.891757000	3.820215000
C	7.543888000	14.507784000	3.800194000
C	6.155897000	10.942810000	6.633514000
C	5.338265000	11.052154000	7.760094000
H	4.261056000	11.122022000	7.620700000
C	10.729449000	5.947808000	5.492649000
H	11.687274000	5.491030000	5.241386000
H	10.055741000	5.145698000	5.816983000
H	10.885084000	6.593171000	6.366132000
C	8.677992000	14.822213000	2.976600000
C	7.261718000	8.360277000	2.621441000
H	6.904627000	9.092520000	3.347947000
H	6.500616000	7.575982000	2.521026000
H	7.351572000	8.862734000	1.657119000
C	10.611257000	6.717982000	2.989386000
C	13.323761000	8.682612000	4.682595000
C	7.827879000	10.091929000	-1.699622000
C	5.859383000	11.082971000	9.053193000
C	7.148495000	14.202252000	6.361311000
H	6.200915000	13.723007000	6.110535000
H	6.917114000	15.158078000	6.849090000
H	7.643993000	13.564433000	7.096814000
C	9.221118000	10.119729000	-1.669687000
H	9.778531000	9.742931000	-2.525470000
C	9.634670000	7.399854000	2.203522000
C	13.243159000	10.694976000	3.569839000
C	12.579527000	9.435333000	7.044458000
H	12.178454000	10.328665000	7.523155000
H	13.508723000	9.168270000	7.562777000
H	11.869281000	8.620203000	7.228117000
C	11.768407000	5.970116000	2.403639000
H	12.362639000	6.582669000	1.719259000
H	11.408035000	5.110122000	1.825083000
H	12.437106000	5.582800000	3.173638000
C	12.420998000	12.240704000	5.467997000
H	11.845345000	12.828772000	4.747503000
H	13.313617000	12.822784000	5.732214000
H	11.802583000	12.135753000	6.359701000
C	11.430811000	10.553908000	-0.601553000
H	11.864806000	11.545546000	-0.431822000
H	11.791447000	10.167613000	-1.559223000
H	11.826320000	9.911703000	0.196423000
C	5.514023000	10.936064000	5.274997000
H	5.963503000	11.688789000	4.620344000
H	4.439713000	11.128295000	5.347507000
H	5.651899000	9.973887000	4.770165000
C	9.678687000	7.537812000	0.714603000
H	8.961689000	8.274853000	0.352735000
H	9.447847000	6.578566000	0.233151000
H	10.666036000	7.843273000	0.353337000
C	8.705012000	15.031693000	1.497411000
H	9.422228000	14.352801000	1.020205000
H	9.000561000	16.058727000	1.253223000
H	7.727948000	14.853187000	1.042122000
C	13.633000000	11.758760000	2.594692000
H	13.496379000	11.441299000	1.559361000
H	14.693860000	12.010372000	2.733838000
H	13.048868000	12.670311000	2.728579000

C	6.106197000	14.493282000	3.381069000
H	5.999503000	14.469971000	2.294714000
H	5.599230000	15.396872000	3.739379000
H	5.562363000	13.634309000	3.784657000
C	9.573376000	10.775564000	8.329792000
H	9.927209000	9.761244000	8.117145000
H	9.836312000	11.016319000	9.363765000
H	10.119976000	11.438339000	7.651908000
C	11.203378000	15.212825000	3.339601000
H	11.948692000	15.077718000	4.127077000
H	11.267231000	16.254116000	3.001726000
H	11.482241000	14.571211000	2.496099000
C	14.159241000	8.698491000	2.208758000
H	14.280595000	7.619039000	2.317847000
H	15.152028000	9.123462000	2.017258000
H	13.563834000	8.880082000	1.305797000
C	13.809838000	7.329377000	5.093524000
H	13.243985000	6.930735000	5.936777000
H	14.858550000	7.394683000	5.411990000
H	13.767719000	6.601644000	4.282028000
C	10.229492000	14.678049000	6.408769000
H	10.055805000	13.793226000	7.029934000
H	9.977085000	15.555653000	7.015624000
H	11.296911000	14.728198000	6.186398000
C	4.957676000	11.179804000	10.252872000
H	4.697802000	10.184195000	10.633724000
H	4.020934000	11.689748000	10.009019000
H	5.438400000	11.724739000	11.071124000
C	7.080342000	9.583798000	-2.901487000
H	6.146419000	9.091666000	-2.612460000
H	7.679056000	8.866324000	-3.470474000
H	6.817555000	10.402485000	-3.583097000

Compound **3** (U-P-Al)

Multiplicity = 3

U	4.624278347	4.763037898	3.490018214
P	3.388116959	2.998259342	1.700634938
P	3.455962446	3.071332529	5.228689439
Al	2.850487876	1.586720010	3.522407550
C	6.820472163	4.328082843	1.953010596
C	7.340316630	4.883155249	4.126089596
C	4.560043849	7.393810311	4.025320298
C	7.235173344	5.394254279	2.802025221
C	1.982466698	3.534936758	-0.657957761
C	7.013510202	3.500431057	4.088528653
C	6.700028986	3.158800930	2.746853820
C	2.835991113	3.108768549	6.960678574
C	1.848873980	3.322212066	0.735609204
C	2.652631091	6.588577379	3.000545420
C	1.468310690	2.916071729	7.265977175
C	3.908257067	7.225462148	2.770287990
C	2.536029868	6.352902681	4.395109646
C	1.588260424	6.442226531	1.960188198
H	1.956720171	5.985847932	1.038269639
H	1.174557260	7.426557440	1.702329660
H	0.762126366	5.823522070	2.308579401
C	3.727643967	6.805963551	5.019865678
C	0.847547856	3.756316102	-1.438578755
H	0.972519371	3.913258792	-2.508621958
C	-0.436822413	3.777402022	-0.893240406
C	4.346646011	7.728029507	1.427933138
H	5.240297869	8.350962412	1.501751337
H	3.558739389	8.335521973	0.968249323
H	4.562258371	6.917077809	0.718749035
C	1.899666783	3.350205119	9.631263374
C	3.645005303	-0.513612707	3.891071158
C	1.032979406	3.032713830	8.587648737
H	-0.022824978	2.879213470	8.803155378
C	5.713011286	8.296763126	4.332477941
H	6.417349803	7.860441561	5.044452777
H	5.338312729	9.224371102	4.783847941

H	6.267185281	8.574694267	3.436350190
C	6.678547618	4.410363286	0.465675910
H	6.007590555	3.633619208	0.092907861
H	7.650338085	4.294326902	-0.032177543
H	6.274005529	5.377232658	0.147551389
C	2.419782639	-0.336783401	4.603598512
C	-0.556768794	3.580279581	0.480768940
H	-1.544630739	3.608000166	0.937545602
C	1.966661824	-0.240420663	2.333319649
C	3.727951101	3.419360256	8.015427470
C	3.245282823	3.543240740	9.317945568
H	3.946159536	3.788848242	10.113588592
C	3.350296222	-0.488831276	2.489635936
C	3.331782406	3.535004853	-1.318645695
H	3.954618021	4.357496531	-0.949367872
H	3.238936495	3.638992389	-2.403507867
H	3.879903713	2.613238353	-1.093257378
C	6.464306505	1.766378285	2.267253679
H	5.696706435	1.259440260	2.858058138
H	7.385097831	1.173117650	2.347431716
H	6.131099149	1.751263786	1.230116712
C	1.376581264	-0.147760744	3.639312563
C	0.554708919	3.363878419	1.297598914
C	0.450666831	2.613153018	6.207031745
H	0.140385756	3.520718178	5.681249750
H	-0.442892881	2.154879953	6.640738962
H	0.859906738	1.944127552	5.449229286
C	3.983353521	6.819161443	6.495308777
H	3.479359791	5.989765537	6.998303765
H	3.622211528	7.750336709	6.952004989
H	5.051504026	6.748218619	6.724279434
C	7.201838882	2.506778998	5.190848377
H	7.452859613	2.998197244	6.132311123
H	8.023955849	1.822191001	4.945076851
H	6.300417030	1.911661342	5.355736525
C	1.309211190	5.933538073	5.134866789
H	0.618177925	5.384066135	4.495654678
H	0.777252654	6.820277849	5.504427289
H	1.550881592	5.307341678	5.995664646
C	4.955488794	-0.791384105	4.557060681
H	5.793234821	-0.718789882	3.860651504
H	4.968904808	-1.799909299	4.987966529
H	5.137241806	-0.083719280	5.372522081
C	4.302647420	-0.648028115	1.350926195
H	4.338768811	0.267819475	0.748774986
H	3.987209075	-1.469076891	0.697897472
H	5.315466507	-0.867678515	1.694608483
C	0.348467628	3.205312340	2.769277563
H	0.424510429	2.154812441	3.076420478
H	-0.642572107	3.548310554	3.082194839
H	1.115968856	3.745828127	3.331741288
C	7.850121494	5.602638935	5.337810646
H	8.197755597	6.607452231	5.090047292
H	8.697626957	5.065689914	5.778698481
H	7.096176261	5.699781114	6.128388280
C	1.410007449	3.450313632	11.049295309
H	0.341485933	3.680975529	11.087660818
H	1.945492755	4.227381037	11.603400034
H	1.560550169	2.506263108	11.587622350
C	-1.641272559	4.040641922	-1.754543241
H	-1.759200858	5.112479681	-1.956880909
H	-2.560445503	3.695973873	-1.272033747
H	-1.558393348	3.537056251	-2.723062746
C	7.754097943	6.700532446	2.287861587
H	7.040408387	7.216615362	1.640953217
H	8.657205383	6.528127027	1.689112421
H	8.026218664	7.379878397	3.095855111
C	-0.094740490	-0.167874176	3.915444305
H	-0.348619347	0.281389719	4.877411464
H	-0.452291757	-1.204555610	3.931879095

H	-0.663135882	0.354190719	3.141073656
C	1.260464615	-0.095727871	1.023598020
H	0.344817130	0.492914354	1.116316557
H	0.988880235	-1.073563513	0.606115446
H	1.892162219	0.419070773	0.294589856
C	5.198720571	3.583477182	7.772489604
H	5.660769850	2.615000312	7.556141994
H	5.698579194	4.015448179	8.643905465
H	5.394729004	4.214974138	6.901553018
C	2.291000206	-0.392614831	6.090865750
H	2.944232811	0.339786200	6.577777823
H	2.565316130	-1.385501436	6.466527356
H	1.270104901	-0.185399850	6.416797924

U-Compound **4** (U-As-Al)
Multiplicity = 3

U	4.578089000	4.752068000	3.251487000
As	3.469335000	2.615086000	1.463015000
As	3.390793000	3.085548000	5.122510000
Al	2.928659000	1.326535000	3.509827000
C	6.791127000	4.178512000	1.746911000
C	7.242890000	4.918034000	3.881562000
C	4.488294000	7.387175000	4.047611000
C	7.166601000	5.315346000	2.514241000
C	1.933910000	4.012061000	-0.538625000
C	6.934671000	3.535054000	3.950165000
C	6.656478000	3.078101000	2.633933000
C	2.722190000	3.123464000	6.954592000
C	1.811895000	3.376669000	0.721160000
C	2.782817000	6.694220000	2.662112000
C	1.355556000	2.902432000	7.231596000
C	4.053011000	7.338665000	2.691762000
C	2.445261000	6.329383000	3.990562000
C	1.889913000	6.642201000	1.464341000
H	2.437244000	6.417414000	0.546179000
H	1.405110000	7.616997000	1.318208000
H	1.104552000	5.892098000	1.560872000
C	3.512383000	6.730149000	4.842969000
C	0.812437000	4.568888000	-1.154630000
H	0.935627000	5.060075000	-2.118460000
C	-0.452176000	4.531187000	-0.568265000
C	4.651615000	8.041137000	1.511576000
H	5.625323000	8.472291000	1.745603000
H	3.998095000	8.859104000	1.183716000
H	4.778433000	7.380459000	0.645142000
C	1.749168000	3.285702000	9.612042000
C	3.862112000	-0.673520000	4.135319000
C	0.899774000	2.982457000	8.549971000
H	-0.156157000	2.808145000	8.748417000
C	5.592955000	8.230750000	4.598531000
H	6.021117000	7.808907000	5.509532000
H	5.202025000	9.223017000	4.858833000
H	6.403489000	8.386772000	3.884521000
C	6.687430000	4.125020000	0.255199000
H	6.004141000	3.334107000	-0.062944000
H	7.668282000	3.936686000	-0.200263000
H	6.317694000	5.066878000	-0.162911000
C	2.658835000	-0.486020000	4.878717000
C	-0.565368000	3.905124000	0.671781000
H	-1.537729000	3.864894000	1.159933000
C	2.109904000	-0.655595000	2.635849000
C	3.596424000	3.420604000	8.023350000
C	3.096386000	3.503786000	9.323185000
H	3.782602000	3.739461000	10.134565000
C	3.513965000	-0.806353000	2.752059000
C	3.267044000	4.149633000	-1.216583000
H	3.955755000	4.752836000	-0.611637000
H	3.166072000	4.628920000	-2.194575000
H	3.753715000	3.176839000	-1.347295000
C	6.488736000	1.642844000	2.265648000
H	5.838956000	1.128809000	2.977968000
H	7.459002000	1.128442000	2.279954000

H	6.051758000	1.522846000	1.273833000
C	1.569981000	-0.452990000	3.952843000
C	0.533771000	3.329720000	1.314958000
C	0.362031000	2.589564000	6.150898000
H	0.100004000	3.481276000	5.573917000
H	-0.561104000	2.181226000	6.572015000
H	0.769457000	1.871848000	5.436003000
C	3.525418000	6.641145000	6.336790000
H	2.967099000	5.774848000	6.697220000
H	3.079326000	7.537252000	6.787681000
H	4.543773000	6.559842000	6.728795000
C	7.151398000	2.651174000	5.135338000
H	7.178217000	3.226186000	6.061537000
H	8.112845000	2.127697000	5.043285000
H	6.363504000	1.902151000	5.230467000
C	1.113096000	5.826921000	4.440935000
H	0.673203000	5.140570000	3.716246000
H	0.417348000	6.668499000	4.559641000
H	1.188191000	5.314967000	5.401188000
C	5.210285000	-0.844826000	4.760479000
H	6.016296000	-0.767863000	4.027761000
H	5.293407000	-1.828900000	5.237673000
H	5.389647000	-0.092716000	5.535089000
C	4.426684000	-1.049596000	1.595124000
H	4.316319000	-0.260862000	0.841862000
H	4.192984000	-2.007332000	1.115399000
H	5.474036000	-1.080767000	1.902114000
C	0.322319000	2.674928000	2.641203000
H	0.499799000	1.596752000	2.564303000
H	-0.696295000	2.815787000	3.013842000
H	1.019436000	3.069691000	3.391048000
C	7.771964000	5.720555000	5.030114000
H	7.939223000	6.761363000	4.751939000
H	8.731776000	5.313753000	5.371258000
H	7.098436000	5.711427000	5.894762000
C	1.239823000	3.345865000	11.025600000
H	0.163754000	3.539503000	11.054699000
H	1.742038000	4.131690000	11.598336000
H	1.416779000	2.399032000	11.550706000
C	-1.650214000	5.123147000	-1.257914000
H	-1.383230000	6.021810000	-1.822818000
H	-2.430296000	5.393870000	-0.540047000
H	-2.092449000	4.413513000	-1.968417000
C	7.670404000	6.597414000	1.931387000
H	7.157929000	6.858125000	1.002988000
H	8.737768000	6.504374000	1.692821000
H	7.567982000	7.438266000	2.618887000
C	0.114490000	-0.492551000	4.303070000
H	-0.063463000	-0.167828000	5.329460000
H	-0.260587000	-1.518936000	4.213593000
H	-0.497942000	0.133613000	3.648718000
C	1.349297000	-0.692674000	1.349086000
H	0.281095000	-0.525607000	1.508625000
H	1.460044000	-1.664467000	0.853962000
H	1.707992000	0.075520000	0.653220000
C	5.066244000	3.619841000	7.798402000
H	5.540828000	2.678001000	7.504248000
H	5.560162000	3.985136000	8.703116000
H	5.253186000	4.326486000	6.984342000
C	2.595144000	-0.355787000	6.364722000
H	3.298891000	0.400718000	6.726985000
H	2.842494000	-1.306096000	6.852896000
H	1.604052000	-0.052344000	6.706700000

Compound **6** (U-N-Al)

Multiplicity = 3

U	8.036732000	2.676294000	14.835361000
Al	10.183498000	4.007001000	13.137748000
N	9.213134000	4.540201000	14.624024000
N	9.465650000	2.298593000	13.185274000
C	7.795626000	1.797417000	17.478281000

C	8.994607000	2.556254000	17.379213000
C	9.907173000	1.833468000	16.559832000
C	9.261561000	0.635734000	16.143058000
C	7.960304000	0.613054000	16.715900000
C	6.654493000	2.160718000	18.376664000
H	6.197846000	3.122813000	18.117378000
H	6.997803000	2.248005000	19.414902000
H	5.866960000	1.405056000	18.355821000
C	9.253821000	3.817796000	18.143386000
H	10.199644000	4.278742000	17.858403000
H	9.289990000	3.613487000	19.221397000
H	8.479144000	4.576411000	17.984696000
C	11.323907000	2.215515000	16.262237000
H	11.599436000	1.947814000	15.238193000
H	12.025753000	1.710049000	16.937715000
H	11.469934000	3.291539000	16.376438000
C	9.867267000	-0.498606000	15.376633000
H	9.250052000	-0.821317000	14.530690000
H	10.005096000	-1.371744000	16.027853000
H	10.841116000	-0.231574000	14.966252000
C	7.034388000	-0.560367000	16.630186000
H	6.117696000	-0.394687000	17.199261000
H	7.516152000	-1.456956000	17.039364000
H	6.746037000	-0.805851000	15.601366000
C	6.090463000	4.154661000	13.545869000
C	5.519198000	3.795572000	14.797966000
C	5.287267000	2.391914000	14.791017000
C	5.743760000	1.880927000	13.544360000
C	6.227995000	2.970796000	12.770261000
C	6.388130000	5.535429000	13.048441000
H	5.893027000	6.295941000	13.658345000
H	6.029337000	5.660487000	12.020806000
H	7.460577000	5.752828000	13.059731000
C	5.137575000	4.731093000	15.906989000
H	5.932988000	4.881410000	16.650102000

H	4.261031000	4.361085000	16.446108000
H	4.886493000	5.722943000	15.518450000
C	4.441008000	1.643641000	15.773629000
H	4.721031000	0.591772000	15.844923000
H	3.386396000	1.677402000	15.467819000
H	4.498571000	2.068682000	16.776379000
C	5.642730000	0.448607000	13.110437000
H	5.545621000	0.369671000	12.023421000
H	4.766364000	-0.033818000	13.552743000
H	6.517191000	-0.155294000	13.388685000
C	6.686574000	2.936434000	11.345029000
H	7.776310000	2.990486000	11.260341000
H	6.263032000	3.777449000	10.785346000
H	6.364882000	2.019209000	10.843725000
C	10.202380000	5.229092000	11.252554000
C	11.011350000	4.071123000	11.024032000
C	12.097735000	4.111875000	11.944309000
C	11.975816000	5.316076000	12.725110000
C	10.814140000	6.012038000	12.282254000
C	9.017892000	5.642744000	10.436679000
H	8.475091000	4.782049000	10.040563000
H	9.338399000	6.250302000	9.581344000
H	8.314642000	6.242604000	11.017885000
C	10.749812000	3.029956000	9.983310000
H	11.325256000	2.121269000	10.166001000
H	11.007556000	3.408213000	8.986722000
H	9.696889000	2.735433000	9.963343000
C	13.303712000	3.225931000	11.958768000
H	13.608335000	2.952189000	12.973974000
H	14.153954000	3.744646000	11.498509000
H	13.132261000	2.304225000	11.401699000
C	13.040460000	5.821508000	13.647930000
H	12.697707000	6.679452000	14.227708000
H	13.918289000	6.135909000	13.069753000
H	13.379531000	5.055203000	14.352668000

C	10.312334000	7.334107000	12.768032000
H	9.239826000	7.309210000	12.982082000
H	10.482564000	8.110669000	12.012705000
H	10.807701000	7.637707000	13.690859000
C	9.385288000	5.664116000	15.416312000
C	10.650231000	6.016068000	15.929952000
H	11.494448000	5.382969000	15.681672000
C	10.821612000	7.121119000	16.755548000
H	11.813304000	7.355630000	17.134759000
C	9.731690000	7.918022000	17.108959000
H	9.863509000	8.780597000	17.754715000
C	8.469190000	7.579831000	16.623646000
H	7.605420000	8.183898000	16.890324000
C	8.297057000	6.476269000	15.792979000
H	7.314886000	6.230276000	15.409978000
C	9.886651000	1.162139000	12.511639000
C	8.985219000	0.350115000	11.795181000
H	7.948790000	0.656706000	11.738985000
C	9.406860000	-0.810431000	11.152429000
H	8.681531000	-1.408790000	10.606625000
C	10.742994000	-1.205640000	11.196801000
H	11.070199000	-2.109583000	10.692537000
C	11.650673000	-0.421219000	11.910066000
H	12.694743000	-0.718012000	11.973502000
C	11.229482000	0.734893000	12.556476000
H	11.932731000	1.322885000	13.135550000

Compound **5** (Th-N-Al)

Multiplicity = 1

Th	1.551939000	2.644520000	3.334500000
Al	-0.642460000	4.017515000	5.060548000
N	0.338721000	4.556781000	3.589591000
N	0.066777000	2.310160000	5.032007000
C	-1.463064000	4.087060000	7.176850000
C	3.906662000	1.845530000	4.657789000
C	3.558703000	4.120322000	4.658296000
C	3.242777000	5.500033000	5.149803000
H	3.747911000	6.262717000	4.550874000
H	3.576790000	5.626313000	6.185404000
H	2.169467000	5.714061000	5.114195000
C	4.148821000	3.764892000	3.413627000
C	3.410440000	2.932550000	5.428941000
C	1.564516000	0.560001000	1.408205000
C	-0.348349000	1.152561000	5.668038000
C	0.194867000	5.654809000	2.760076000
C	-1.249372000	6.026254000	5.919926000
C	1.736034000	1.745430000	0.645578000
C	-2.419163000	5.342631000	5.479436000
C	-2.551744000	4.139618000	6.260347000
C	-2.095939000	-0.467424000	6.222640000
H	-3.140199000	-0.764898000	6.163960000
C	-0.380516000	1.785229000	1.552505000
C	-1.691430000	0.721997000	5.628138000
H	-2.406893000	1.335631000	5.091791000
C	0.536201000	2.505214000	0.737545000
C	-1.057813000	6.030609000	2.230431000
H	-1.924981000	5.441766000	2.507940000
C	-3.762066000	3.259636000	6.242660000
H	-4.067031000	2.990670000	5.226134000
H	-4.610978000	3.779252000	6.704332000
H	-3.594879000	2.334817000	6.796167000
C	-1.199213000	3.034673000	8.205415000
H	-1.790937000	2.135867000	8.025647000
H	-1.432967000	3.407515000	9.209872000
H	-0.150369000	2.724559000	8.202788000
C	0.567368000	0.309044000	6.331305000
H	1.603566000	0.619752000	6.392185000
C	5.168758000	1.600269000	2.402244000
H	4.838030000	0.564160000	2.309021000
H	6.229737000	1.576587000	2.685064000
H	5.110130000	2.055122000	1.411855000

C	0.259318000	0.586988000	1.973535000
C	2.920593000	2.888850000	6.844304000
H	1.828137000	2.933679000	6.906377000
H	3.325015000	3.730461000	7.416774000
H	3.238276000	1.971095000	7.347330000
C	1.311606000	6.410476000	2.344711000
H	2.285153000	6.151520000	2.744318000
C	4.538615000	4.699185000	2.306646000
H	3.781721000	4.781851000	1.513951000
H	5.466643000	4.373687000	1.827613000
H	4.702913000	5.712481000	2.684232000
C	-3.474545000	5.856308000	4.550434000
H	-3.118802000	6.707157000	3.967742000
H	-4.351566000	6.184718000	5.121994000
H	-3.819359000	5.090614000	3.847638000
C	-1.172779000	-1.285719000	6.876123000
H	-1.487807000	-2.216414000	7.337579000
C	-0.069325000	7.834088000	0.954183000
H	-0.170202000	8.666323000	0.264576000
C	4.372081000	2.360607000	3.417223000
C	-0.642836000	5.236278000	6.946664000
C	0.561287000	5.624871000	7.745960000
H	1.101081000	4.751833000	8.119263000
H	0.267052000	6.224519000	8.616179000
H	1.260344000	6.223766000	7.158433000
C	-0.345486000	-0.532027000	2.764245000
H	0.277058000	-0.843676000	3.610927000
H	-0.492754000	-1.415976000	2.130272000
H	-1.314371000	-0.253449000	3.179247000
C	0.162231000	-0.886332000	6.918818000
H	0.899358000	-1.508206000	7.420983000
C	3.983984000	0.408262000	5.080193000
H	4.067178000	0.318822000	6.167414000
H	4.857330000	-0.083158000	4.641969000
H	3.103551000	-0.181815000	4.788235000
C	-1.187940000	7.099129000	1.350894000
H	-2.170876000	7.352383000	0.960832000
C	1.180366000	7.474801000	1.457298000
H	2.065750000	8.031933000	1.160600000
C	-0.727165000	7.335348000	5.421145000
H	0.345422000	7.290335000	5.210363000
H	-0.887112000	8.124400000	6.165642000
H	-1.215020000	7.634752000	4.492898000
C	2.499492000	-0.604994000	1.519049000
H	3.415824000	-0.442586000	0.947888000
H	2.027677000	-1.515331000	1.129965000
H	2.790650000	-0.825568000	2.553594000
C	0.289518000	3.783277000	-0.003880000
H	-0.649712000	4.250215000	0.293506000
H	0.248291000	3.600551000	-1.085331000
H	1.072765000	4.530610000	0.167169000
C	2.894633000	2.116039000	-0.228056000
H	3.333114000	3.086280000	0.035734000
H	2.579429000	2.192288000	-1.275805000
H	3.691815000	1.371683000	-0.178999000
C	-1.782812000	2.193212000	1.884582000
H	-2.042635000	1.913495000	2.909987000
H	-2.509924000	1.714756000	1.216577000
H	-1.908946000	3.274492000	1.792788000

mes-ThNAl-S1-disp

Th	1.600782000	2.729809000	3.373308000
Al	-0.607297000	3.964255000	5.123730000
N	0.181943000	4.526279000	3.517516000
N	-0.006020000	2.182654000	4.928647000
C	-1.199236000	4.069220000	7.293520000
C	4.175266000	1.894481000	4.642344000
C	3.680175000	4.141584000	4.709287000
C	3.368539000	5.490067000	5.280657000
H	3.846408000	6.284582000	4.702984000

H	3.755826000	5.567767000	6.303441000
H	2.297207000	5.707124000	5.311315000
C	4.346316000	3.871355000	3.479191000
C	3.572722000	2.915803000	5.429436000
C	1.880905000	0.634540000	1.572370000
C	-0.620264000	1.093410000	5.558717000
C	-0.328658000	5.497578000	2.650778000
C	-0.623309000	6.054937000	6.255368000
C	2.273048000	1.763888000	0.802927000
C	-1.947844000	5.743008000	5.873386000
C	-2.312198000	4.498003000	6.499430000
C	-2.655621000	-0.105454000	6.221745000
H	-3.737245000	-0.206210000	6.145839000
C	0.003706000	1.892329000	1.151867000
C	-2.029052000	0.893135000	5.480160000
C	1.120814000	2.563614000	0.586020000
C	-1.696038000	5.534319000	2.268597000
C	-3.700981000	3.948691000	6.605077000
H	-4.277452000	4.085899000	5.685921000
H	-4.240987000	4.462765000	7.409637000
H	-3.694478000	2.881767000	6.838653000
C	-1.158136000	2.886502000	8.209027000
H	-1.890844000	2.129465000	7.925149000
H	-1.360335000	3.190681000	9.243762000
H	-0.181475000	2.395996000	8.187922000
C	0.107611000	0.092199000	6.248237000
C	5.680776000	1.851065000	2.563333000
H	5.440667000	0.817995000	2.299549000
H	6.645892000	1.820983000	3.088869000
H	5.837404000	2.407740000	1.638965000
C	0.473699000	0.715560000	1.789849000
C	3.113074000	2.794519000	6.850116000
H	2.023299000	2.753371000	6.946979000
H	3.466681000	3.646694000	7.438223000

H	3.514314000	1.891397000	7.315379000
C	0.532028000	6.434450000	2.022944000
C	4.905645000	4.874317000	2.514786000
H	4.588238000	4.703927000	1.479471000
H	6.001945000	4.836553000	2.518491000
H	4.615469000	5.893819000	2.773744000
C	-2.850728000	6.695341000	5.153113000
H	-2.444711000	7.010991000	4.188414000
H	-2.989846000	7.595573000	5.764429000
H	-3.842421000	6.270223000	4.983149000
C	-1.941133000	-0.995055000	7.026292000
C	-1.323758000	7.471815000	0.823042000
C	4.654408000	2.486827000	3.447705000
C	-0.159635000	5.035368000	7.139803000
C	1.052266000	5.124619000	8.010799000
H	1.283455000	4.166550000	8.476490000
H	0.860993000	5.840824000	8.819608000
H	1.939234000	5.464678000	7.477798000
C	-0.346666000	-0.340043000	2.464921000
H	0.071728000	-0.646238000	3.429250000
H	-0.420517000	-1.240403000	1.841864000
H	-1.360473000	0.007880000	2.666352000
C	-0.554284000	-0.896632000	6.980229000
H	0.048066000	-1.623329000	7.524053000
C	4.548553000	0.508937000	5.076077000
H	4.094516000	-0.275799000	4.462768000
H	4.265491000	0.317501000	6.112154000
H	5.635103000	0.375479000	5.005588000
C	-2.160734000	6.509819000	1.383454000
H	-3.216430000	6.502800000	1.116396000
C	0.031289000	7.395454000	1.148458000
H	0.728380000	8.098328000	0.695100000
C	0.116877000	7.273706000	5.806998000
H	1.111785000	7.327121000	6.253628000

H	-0.422232000	8.184069000	6.095193000
H	0.228650000	7.292899000	4.717528000
C	2.723800000	-0.533676000	1.980307000
H	3.787748000	-0.291683000	1.980291000
H	2.578292000	-1.383027000	1.300377000
H	2.464518000	-0.891190000	2.983276000
C	1.061834000	3.803482000	-0.253068000
H	0.320676000	4.515377000	0.119826000
H	0.798183000	3.566328000	-1.292359000
H	2.026776000	4.318736000	-0.277214000
C	3.586053000	2.004745000	0.128260000
H	4.033473000	2.967865000	0.393160000
H	3.451409000	2.004876000	-0.960117000
H	4.309841000	1.226149000	0.366653000
C	-1.412230000	2.240900000	0.833600000
H	-2.120471000	1.859938000	1.570579000
H	-1.686565000	1.796463000	-0.132368000
H	-1.553284000	3.319494000	0.744026000
C	1.596412000	0.009959000	6.150845000
H	2.079072000	-0.046517000	7.131795000
H	1.897815000	-0.884725000	5.593236000
H	2.004802000	0.878287000	5.637278000
C	-2.844266000	1.690578000	4.516961000
H	-2.472210000	1.547917000	3.498846000
H	-3.897436000	1.399180000	4.544350000
H	-2.800955000	2.763501000	4.708123000
C	-2.636640000	-2.024519000	7.872551000
H	-3.550588000	-2.387665000	7.391462000
H	-1.991056000	-2.887105000	8.063710000
H	-2.926743000	-1.613137000	8.847786000
C	2.000768000	6.347757000	2.267833000
H	2.241534000	6.502110000	3.319668000
H	2.384611000	5.354515000	2.002967000
H	2.554247000	7.074582000	1.667582000

C	-1.851550000	8.540023000	-0.094231000
H	-1.133922000	8.779106000	-0.885615000
H	-2.787017000	8.230869000	-0.570267000
H	-2.055152000	9.471534000	0.449179000
C	-2.646907000	4.495655000	2.769946000
H	-3.447619000	4.303690000	2.050110000
H	-2.110896000	3.562935000	2.932431000
H	-3.125027000	4.776327000	3.711901000

mes-UNAl-S3-disp

U	7.991337000	2.706057000	14.802773000
Al	10.078635000	3.931692000	13.035913000
N	9.275929000	4.513058000	14.637029000
N	9.494812000	2.140320000	13.248066000
C	7.266854000	1.884924000	17.360912000
C	8.415005000	2.701052000	17.529427000
C	9.542411000	1.989239000	17.032783000
C	9.075556000	0.773672000	16.464399000
C	7.666891000	0.708931000	16.669613000
C	5.984123000	2.135306000	18.090269000
H	5.475175000	3.050095000	17.778390000
H	6.191574000	2.236940000	19.162613000
H	5.284147000	1.308666000	17.971712000
C	8.451938000	3.981458000	18.306733000
H	9.293493000	4.613542000	18.014728000
H	8.534422000	3.789955000	19.385403000
H	7.540619000	4.569791000	18.156586000
C	10.940002000	2.284646000	17.478439000
H	11.708952000	1.872135000	16.823674000
H	11.091008000	1.836836000	18.469979000
H	11.115376000	3.356424000	17.581802000
C	9.908196000	-0.336664000	15.896730000
H	9.824014000	-0.433473000	14.808309000

H	9.619730000	-1.300656000	16.331646000	C	11.567555000	5.602387000	12.324648000
H	10.966003000	-0.186388000	16.122240000	C	10.276140000	6.041667000	11.955208000
C	6.819482000	-0.475549000	16.318739000	C	8.503180000	5.322141000	10.211804000
H	5.964189000	-0.563539000	16.994333000	H	8.225172000	4.431238000	9.647991000
H	7.397401000	-1.401305000	16.401439000	H	8.716715000	6.114935000	9.484629000
H	6.420145000	-0.443006000	15.298226000	H	7.636266000	5.638084000	10.793146000
C	5.940347000	4.178914000	13.558017000	C	10.542429000	2.886466000	9.924797000
C	5.349128000	3.800842000	14.799176000	H	11.163729000	2.041857000	10.226736000
C	5.091842000	2.403740000	14.749129000	H	10.850354000	3.193072000	8.917082000
C	5.545662000	1.913948000	13.495741000	H	9.515157000	2.519881000	9.860212000
C	6.056297000	3.014023000	12.753438000	C	13.128110000	3.628539000	11.599006000
C	6.152991000	5.566621000	13.039116000	H	13.739158000	3.788905000	12.491491000
H	5.658101000	6.309500000	13.667403000	H	13.698839000	4.008234000	10.742655000
H	5.725050000	5.661219000	12.034304000	H	13.005414000	2.550860000	11.467353000
H	7.210024000	5.838725000	12.983543000	C	12.565749000	6.460341000	13.036746000
C	4.859455000	4.735307000	15.866570000	H	12.166897000	6.887733000	13.960192000
H	5.561696000	4.878986000	16.696010000	H	12.854693000	7.292794000	12.383217000
H	3.922579000	4.370994000	16.298666000	H	13.480588000	5.916688000	13.282169000
H	4.660233000	5.728999000	15.455350000	C	9.646582000	7.319588000	12.406736000
C	4.118819000	1.649479000	15.602723000	H	8.583983000	7.350684000	12.156518000
H	4.484025000	0.672378000	15.923425000	H	10.122634000	8.182855000	11.925029000
H	3.200810000	1.469813000	15.026415000	H	9.742854000	7.449112000	13.488937000
H	3.831299000	2.206749000	16.493518000	C	9.811672000	5.521297000	15.457175000
C	5.222411000	0.544169000	12.976164000	C	11.170003000	5.474921000	15.875448000
H	5.230849000	0.521959000	11.883744000	C	11.695044000	6.445766000	16.727175000
H	4.221098000	0.242680000	13.302242000	H	12.738805000	6.362014000	17.025347000
H	5.912930000	-0.239145000	13.309506000	C	10.926757000	7.502243000	17.213655000
C	6.400666000	3.033217000	11.299659000	C	9.589705000	7.534182000	16.826673000
H	7.477931000	3.033413000	11.121330000	H	8.946913000	8.327623000	17.205123000
H	5.984819000	3.926362000	10.823545000	C	9.025624000	6.579407000	15.978403000
H	5.983437000	2.166434000	10.782461000	C	10.141239000	1.064828000	12.605308000
C	9.718138000	5.087951000	11.052279000	C	9.491434000	0.208465000	11.683954000
C	10.663775000	4.032512000	10.877119000	C	10.198471000	-0.792338000	11.012628000
C	11.809127000	4.332042000	11.686876000	H	9.656761000	-1.410194000	10.297749000

C	11.553236000	-1.024254000	11.220704000
C	12.175756000	-0.244848000	12.195847000
H	13.218540000	-0.434367000	12.445228000
C	11.501715000	0.756998000	12.891168000
C	11.516543000	8.559414000	18.105376000
H	10.763847000	8.979535000	18.779460000
H	12.330664000	8.157934000	18.716711000
H	11.929796000	9.391486000	17.521225000
C	12.045651000	4.356305000	15.423299000
H	12.887352000	4.194334000	16.101420000
H	11.463153000	3.441954000	15.368231000
H	12.464924000	4.535304000	14.430724000
C	7.575886000	6.703879000	15.641773000
H	7.419692000	7.379353000	14.793413000
H	7.163321000	5.739301000	15.356863000
H	7.004689000	7.097905000	16.487235000
C	12.217672000	1.471594000	13.986386000
H	11.579660000	1.525858000	14.867925000
H	13.149258000	0.963526000	14.250059000
H	12.471925000	2.497284000	13.715910000
C	12.308771000	-2.069108000	10.447813000
H	13.052224000	-2.571892000	11.074663000
H	11.635967000	-2.831735000	10.044530000
H	12.847313000	-1.627955000	9.599527000
C	8.037755000	0.350955000	11.383943000
H	7.868349000	0.836203000	10.416324000
H	7.547130000	-0.626281000	11.346598000
H	7.555860000	0.959820000	12.143684000

5. References

1 J. K. Pagano, J. M. Dorhout, R. Waterman, K. R. Czerwinski and J. L. Kiplinger, *Chem. Commun.*, 2015, **51**, 17379–17381.

2 S. Kurz, H. Oesen, J. Sieler and E. Hey-hawkins, *Phosphorus Sulfur Silicon Relat Elem*, 1996, **117**, 189–196.

3 D. S. J. Arney, C. J. Burns and D. C. Smith, *J. Am. Chem. Soc.*, 1992, **114**, 10068–10069.

4 C. J. Burns, W. H. Smith, J. C. Huffman and A. P. Sattelberger, *J. Am. Chem. Soc.*, 1990, **112**, 3237–3239.

5 M. Scheer, *Dalton Trans.*, 2008, 4372.

6. M. L. Tarlton, O. J. Fajen, S. P. Kelley, A. Kerridge, T. Malcomson, T. L. Morrison, M. P. Shores, X. Xhani and J. R. Walensky, *Inorg. Chem.*, 2021, **60**, 10614–10630.

7. S. Schulz, H. W. Roesky, H. J. Koch, G. M. Sheldrick, D. Stalke and A. Kuhn, *Angew. Chem. Inter. Ed.*, 1993., 1729–1731.

8. Apex3, AXScale, and SAINT, version 2017. 3-0, Bruker AXS, Inc., Madison, WI, 2017.

9. Apex4, AXScale, and SAINT, version 2022.1, Bruker AXS, Inc., Madison, WI, 2022.

10 G. M. Sheldrick, *Acta Crystallogr.* C, 2015, **71**, 3-8.

11. O. V. Dolomanov, L. J. Bourhis, R. J. Gildea, J. A. K. Howard, H. Puschmann, *J. Appl. Crystallogr.*, 2009, **42**, 339-341.

12. A. Thorn, B. Dittrich, G. M. Sheldrick, *Acta Cryst. Sect. A. Found. Adv.* 2012, **68**, 448-451.

13. K. Brandenburg, Diamond Version 4.6.0, Crystal Impact GbR, Bonn, 2019.
